# Optimization of the Macrocyclic Tetrapeptide [D-Trp]CJ-15,208 to Prevent Stress-Induced Relapse of Cocaine-Seeking Behavior

**DOI:** 10.3390/molecules30193993

**Published:** 2025-10-05

**Authors:** Jane V. Aldrich, Dmitry Y. Yakovlev, Jeremy S. Coleman, Sanjeewa N. Senadheera, Heather M. Stacy, Shainnel O. Eans, Brian I. Knapp, Jean M. Bidlack, Jay P. McLaughlin

**Affiliations:** 1 Department of Medicinal Chemistry, College of Pharmacy, University of Florida, Gainesville, FL 32610, USA; dmitry.yakov@gmail.com (D.Y.Y.); jeremy.coleman@uga.edu (J.S.C.); 2 Department of Medicinal Chemistry, The University of Kansas, Lawrence, KS 66045, USA; nilendrasns@yahoo.com; 3 Department of Cellular and Systems Pharmacology, College of Pharmacy, University of Florida, Gainesville, FL 32610, USA; heather.stacy12@gmail.com (H.M.S.); shaieans@ufl.edu (S.O.E.); 4 Department of Pharmacology and Physiology, College of Medicine, University of Rochester, Rochester, NY 14642, USA; brian_knapp@urmc.rochester.edu

**Keywords:** [D-Trp]CJ-15,208, kappa opioid receptor antagonist, brain–penetrant peptides, orally active peptides, cocaine abuse, stress-induced reinstatement

## Abstract

Kappa opioid receptor (KOR) antagonists may have therapeutic potential to prevent stress-induced relapse in abstinent individuals with cocaine use disorder (CUD). The macrocyclic peptide [D-Trp]CJ-15,208 (*cyclo*[Phe-D-Pro-Phe-D-Trp]) is an orally bioavailable, brain–penetrant selective KOR antagonist that prevents stress-induced reinstatement of cocaine-seeking behavior in a mouse model of CUD. We synthesized and evaluated analogs of this lead compound with substitutions for the D-Trp residue to identify analogs that exhibit more potent central KOR antagonism following oral administration. The peptides were synthesized by a combination of solid phase and solution peptide synthetic methodologies, and their pharmacological activity was evaluated both in vitro (for KOR affinity, selectivity and antagonism) and in vivo (for antinociception and KOR antagonism), with promising analogs evaluated for their ability to prevent stress-induced reinstatement of cocaine-seeking behavior in the mouse conditioned place preference (CPP) assay. A variety of substituted D-Phe or modified D-Trp derivatives were tolerated by KOR with retention of significant KOR antagonism in vivo after oral administration. Macrocyclic peptide pretreatment, per os, significantly prevented stress-induced reinstatement of cocaine CPP at doses of 10 and 30 mg/kg of [D-Phe^4^]CJ-15,208, **4**, and 30 mg/kg of [D-Trp(formamide)]CJ-15,208, **3**, which are 6-fold and 2-fold lower, respectively, than that needed for {D-Trp]CJ-15,208.

## 1. Introduction

Cocaine use disorder (CUD) is a chronic relapsing condition that affects millions of people worldwide but for which there is currently no approved pharmacotherapy. Cocaine use produces serious medical complications, especially in the cardiovascular system as a result of excessive sympathetic nervous system stimulation and prothrombic effects. Currently there are no approved pharmacological treatments for CUD. One of the challenges in treating CUD and other substance use disorders is the high rate of relapse of abstinent individuals to drug-seeking behavior; relapse rates for psychostimulants are ~50% within one year, increasing to 75% over 5 years without sustained support [1]. Thus, there is a clear need for the development of effective pharmacological treatments for CUD, in particular medications that can prevent relapse. A major trigger of relapse is stress [2], which increases cocaine craving [3]. Elevated stress-induced craving is associated with a shorter time to relapse in cocaine-dependent individuals [4].

Kappa opioid receptors (KORs) remain a promising target for potential therapeutics for CUD, particularly as treatments to prevent relapse. Exposure to stress increases levels of dynorphin peptides, the endogenous agonists for KORs, which in turn activate the receptors. A positron emission tomography study using a KOR-selective agonist before and after a cocaine binge in human subjects with CUD found greater KOR availability was associated with more choices for cocaine [5], supporting the potential application of KOR ligands to treat CUD. KOR antagonists, including several peptide KOR antagonists developed by our labs [6,7,8,9,10,11], prevent stress-induced reinstatement of extinguished cocaine-seeking behavior in animal models.

The macrocyclic peptide natural product CJ-15,208, *cyclo*[Phe-D-Pro-Phe-Trp], was isolated from a fungal fermentation broth [12]. While it posesses structural similarities to endomorphin 1 (Tyr-Pro-Trp-PheNH_2_), it was reported to exhibit KOR antagonism in vitro [12]. Since the stereochemistry of the Trp residue in the natural product CJ-15,208 was not determined, we synthesized both isomers and found that they both bound to KORs with similar affinity [13]. In vivo the D-Trp isomer exhibits selective KOR antagonism after both central (intracerebroventricular, i.c.v.) [8] and systemic, including oral [9], administration and prevents stress-induced reinstatement of cocaine-seeking behavior in mice. However, a high oral dose (60 mg/kg, p.o. (per os)) of [D-Trp]CJ-15,208 was required to prevent reinstatement, spurring an exploration of the structure–activity relationships (SAR) of this lead macrocyclic tetrapeptide to identify analogs that exhibit more potent central KOR antagonism following oral administration.

Previously, we performed an alanine scan of [D-Trp]CJ-15,208 and found that substitution of D-Trp by D-Ala resulted in the largest decrease (79-fold) in KOR affinity, indicating the importance of this residue for KOR affinity [10]. Incorporation of other bicyclic aromatic amino acids (D-benzothienylalanine (D-Bta), D-1’-naphthylalanine or D-2’-naphthylalanine) in this position were tolerated by KOR (K_i_ = 4.3–21.4 nM vs. 21.8 nM for [D-Trp]CJ-15,208), indicating the indole NH was not necessary for KOR interaction. However, only the D-Bta derivative, in which the indole NH is replaced by sulfur, exhibited significant KOR antagonist activity in vivo (at 30 nmol, i.c.v.) [14]. In contrast, the D-2’-naphthylalanine derivative exhibited weak antinociceptive activity (ED_50_ (and 95% confidence interval (CI)) = 31.2 nmol, i.c.v. (22.5–41.9 nmol)) mediated by mu opioid receptors (MORs) while the D-1’-naphthylalanine analog did not exhibit either appreciable antinociception (agonist activity) or significant antagonist activity in vivo.

**Figure 1:**
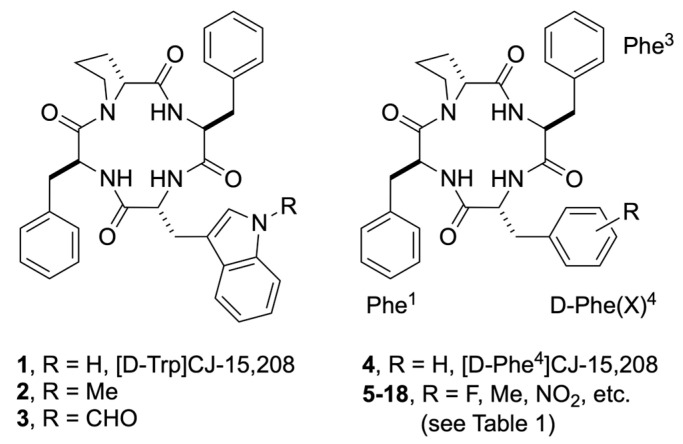
Structures of [D-Trp]CJ-15,208 and the analogs with D-Trp or D-Phe derivatives substituted for D-Trp. The residue numbering used in the analogs is shown for [D-Phe(X)^4^]CJ-15,208.

Therefore for the present analogs we focused on incorporating D-Phe and its analogs plus D-Trp derivatives in place of the D-Trp residue (Figure 1). These studies resulted in the identification of analogs with potent central KOR antagonism in vivo after oral administration, including two that prevented stress-induced reinstatement of cocaine-seeking behavior in a mouse model of CUD.

## 2. Results

### 2.1. Design and Synthesis

Derivatives of D-Phe containing a wide variety of substitutions on the phenyl ring are readily available, facilitating the preparation of a series of [D-Phe^4^]CJ-15,208 derivatives (Figure 1) to explore the SAR of this residue. Fluorine substitution at different positions on the phenyl ring of D-Phe^4^ (analogs **5**–**12**) was explored to minimize the oxidative metabolism of this residue; other substituted D-Phe derivatives were incorporated to assess the tolerance by the KOR for hydrophilic vs. hydrophobic substitutions on this residue Two derivatives of D-Trp with a modification on the indole nitrogen, either a methyl group (**2**) or the formamide derivative (**3**), were also prepared to decrease the number of hydrogen bond donors in the molecule which could potentially facilitate intestinal and blood–brain barrier penetration.

**Scheme 1:**
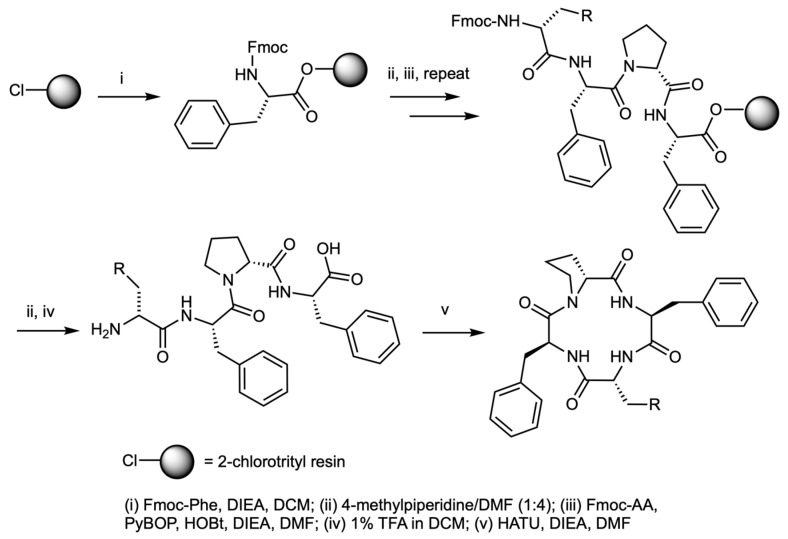
Synthesis of analogs of [D-Trp]- and [D-Phe^4^]CJ-15,208 analogs. The linear peptides D-aa^4^-Phe-D-Pro-Phe (where aa^4^ = D-Trp(R) or D-Phe(R)) were assembled on the 2-chlorotrityl resin, followed by cleavage of the peptide from the resin and cyclization in solution.

The analogs were synthesized as previously described [10,13] by a combination of solid phase synthesis of the linear precursors followed by cyclization in solution (Scheme 1). The cyclic peptides were purified by either silica gel flash chromatography or by reversed phase HPLC, depending upon their hydrophobicity.

### 2.2. Opioid Receptor Affinity and KOR Antagonism In Vitro

#### 2.2.1. Opioid Receptor Affinity

Table 1 reports the K_i_ values for the peptides inhibition of [^3^H]U69,593 binding to the human KOR and [^3^H][D-Ala^2^,*N*-MePhe^4^,glyol]enkephalin ([^3^H]DAMGO) binding to the human MOR. Aticaprant and navacaprant, which underwent clinical trials for major depression (see refs. [15,16] and https://clinicaltrials.gov, accessed on 21 July 2025), were also evaluated for comparison. [D-Phe^4^]CJ-15,208 (**4**) exhibits 3.5-fold lower KOR affinity than [D-Trp]CJ-15,208 (**1**) but comparable affinity to navacaparant. Most of the modifications to D-Trp or D-Phe examined were well tolerated by the KOR. While methylation of the indole nitrogen of D-Trp in **2** resulted in a slight decrease in KOR affinity, the formamide group on this nitrogen in **3** increased KOR affinity relative to the parent peptide **1**, resulting in the peptide with the highest KOR affinity (K_i_ = 2.2 nM) among the analogs. Aticaprant had only slightly higher affinity for the KOR with a K_i_ value of 0.77 nM. Incorporating one or more fluorines in the phenyl ring of D-Phe^4^ (analogs **5**–**12**) was well tolerated, except for the pentafluoro substitution (analog **12**) which caused a 13-fold decrease in KOR affinity. The 3,4-difluoro analog **8** exhibited high KOR affinity (K_i_ = 6.6 nM), comparable to that of [D-Trp]CJ-15,208, and the second highest KOR affinity among the analogs tested. *m*- and *p*-Methyl substitution (in analogs **14** and **15**, respectively), but not ortho substitution (in **13**), were also tolerated, as was *p*-nitro substitution in analog **16** (K_i_ = 7.0 nM). The aniline derivative **17** exhibited negligible KOR affinity, while the Tyr(Me) derivative **18** also exhibited low KOR affinity.

Analogs retaining KOR affinity were evaluated for their MOR affinity to determine their selectivity for the KOR over the MOR. The analogs’ affinities for the MOR varied over a 10-fold range (K_i_ = 36–350 nM), resulting in a range of selectivities (from 2 to 40-fold) for the KOR over the MOR. The D-Trp derivative **3** had the greatest selectivity among the peptides, with a 40-fold preference for the KOR, comparable to that of navacaprant, which had lower affinity for the KOR (K_i_ = 21 nM). This contrasts with the D-Trp(Me) analog **2** which exhibited only a 2-fold difference in the affinities for the KOR and the MOR. For the [D-Phe^4^]CJ-15,208 analogs with fluorine substitution on the phenyl ring, MOR affinity varied over a 7.6-fold range, with analogs containing a *p*-fluorine exhibiting the highest MOR affinities (except for the 2,4,5-trifluoro derivative **10**). Except for the 3,4,5-trifluoro derivative **11**, the analogs with multiple fluorines retained the modest selectivity for the KOR over the MOR observed for the parent peptide [D-Phe^4^]CJ-15,208. The *p*-methyl and *p*-nitro analogs (**15** and **16**, respectively) also exhibited comparable KOR selectivity to the parent peptide, while the other analogs exhibited lower KOR selectivity (≤5-fold preference for the KOR).

#### 2.2.2. KOR Antagonism

Promising analogs were evaluated for both KOR agonist and antagonist activity in the cAMP functional activity. All of the peptides exhibited minimal agonist activity; at a concentration of 10 μM they inhibited forskolin-stimulated cAMP mediated by the KOR by less than 20% that of the reference full agonist U50,488. The antagonist potency varied over a 10-fold range (IC_50_ = 49–460 nM). Peptide **3** had the highest potency (IC_50_ = 49 nM) for inhibiting the U50,488-stimulated decrease in cAMP. [D-Phe^4^]CJ-15,208, **4**, exhibited the lowest potency among the peptides (IC_50_ = 460 nM) while the analogs tested had comparable to ~3-fold higher potency compared to this peptide. Aticaprant was an inverse agonist in this assay, as shown by its stimulation of cAMP levels. It had the lowest IC_50_ value (8 nM) for the inhibition of U50,488-stimulted decrease in cAMP, which is expected given that it is an inverse agonist. Navacaprant was an antagonist with an IC_50_ value of 69 nM for blocking the U50,488 inhibition of cAMP levels, comparable to that of [D-Trp]CJ-15,208.

### 2.3. Metabolic Stability

In contrast to linear peptides, the macrocyclic peptides are stable to proteolytic metabolism in blood but undergo oxidative metabolism in mouse liver microsomes [17]. Therefore we examined the metabolic stability of promising [D-Trp]CJ-15,208 analogs in both human and mouse liver microsomes (Appendix A Table A1). Structural modification, notably substitution with fluorines, is one approach to enhance metabolic stability. However, similar to the lead peptide [D-Trp]CJ-15,208, the analogs were rapidly metabolized by both human and mouse liver microsomes, with the peptides more rapidly metabolized by mouse liver microsomes than human liver microsomes. Only the D-Phe(*p*-NO_2_)^4^ analog **16** exhibited greater metabolic stability in liver microsomes from both species (t_1/2_ = 6.7 and 4.4 min in human and mouse liver microsomes, respectively) than [D-Trp]CJ-15,208.While [D-Trp(Me)]CJ-15,208, **3**, was slightly more stable (t_1/2_ = 3.3 min) than [D-Trp]CJ-15,208 in mouse liver microsomes it was less stable (t_1/2_ =2.4 min) in human liver microsomes.

### 2.4. In Vivo Pharmacological Evaluation

Promising analogs were evaluated in vivo using the 55 °C warm-water tail-withdrawal (WWTW) assay to characterize their KOR antagonism, their ability to cross the blood–brain barrier after oral administration to antagonize a centrally administered KOR agonist, their KOR selectivity and the duration of KOR antagonism. The 55 °C WWTW assay is well established for evaluating compounds antagonizing opioid agonists [18,19,20]. The results from this assay were used to select analogs and doses for evaluation in a mouse CUD model to prevent stress-induced reinstatement of cocaine use disorder.

#### 2.4.1. Evaluation in the 55 °C Warm-Water Tail-Withdrawal Assay for Antinociception and KOR Antagonism

##### Screening of the [D-Trp]- and [D-Phe^4^]CJ-15,208 Analogs

**Figure 2:**
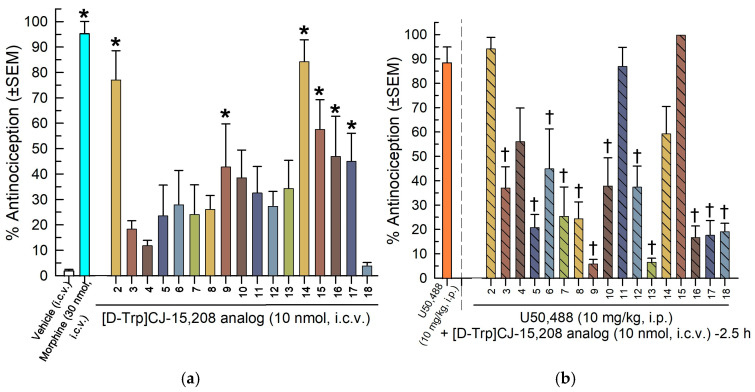
Screening of the [D-Trp]- and [D-Phe^4^]CJ-15,208 analogs **2–18** for antinociception and KOR antagonism following i.c.v. administration. (**a**) The [D-Trp]- and [D-Phe^4^]CJ-15,208 analogs (10 nmol) were administered, and the mice were tested 20 min later for antinociception in the 55 °C WWTW assay. Vehicle alone (50% DMSO/50% saline; left-most bar) was tested as a negative control, and morphine (30 nmol, i.c.v.) was tested as a positive control. (**b**) Two and a half hours later the KOR agonist U50,488 (10 mg/kg, i.p.) was administered, and the mice were tested again in the 55 °C WWTW assay to assess [D-Trp]CJ-15,208 analog-mediated antagonism of U50,488-mediated antinociception. Points represent average % antinociception ± SEM from eight mice for each bar (*n* = 12 for U50,488). * Significantly different from vehicle treatment group; † significantly different from U50,488 treatment group (*p* < 0.05, one-way ANOVA with Dunnett’s multiple comparisons post hoc test).

The [D-Trp]- and [D-Phe^4^]CJ-15,208 analogs **2**–**18** were first tested in the 55 °C WWTW assay following i.c.v. administration (10 nmol). Twenty minutes after treatment, 6 of the 17 analogs tested produced significant antinociception compared to the vehicle (F_(18,132)_ = 7.44, *p* < 0.0001, one-way ANOVA with Dunnett’s post hoc; Figure 2a). The positive control morphine (30 nmol, i.c.v.) also produced significant antinociception compared to the vehicle (*p* < 0.0001). Mice were then tested 2.5 h later to ensure the completion of antinociception induced by [D-Trp]CJ-15,208 analog treatment. After baseline responses were confirmed, potential KOR antagonism by the [D-Trp]CJ-15,208 analogs was evaluated by administering mice the KOR agonist U50,488 (10 mg/kg, i.p.). The majority (12) of the analogs induced significant antagonism of U50,488-mediated antinociception in the 55 °C WWTW assay (F_(17,129)_ = 13.3, *p* < 0.0001, one-way ANOVA with Dunnett’s post hoc test; Figure 2b).

**Figure 3:**
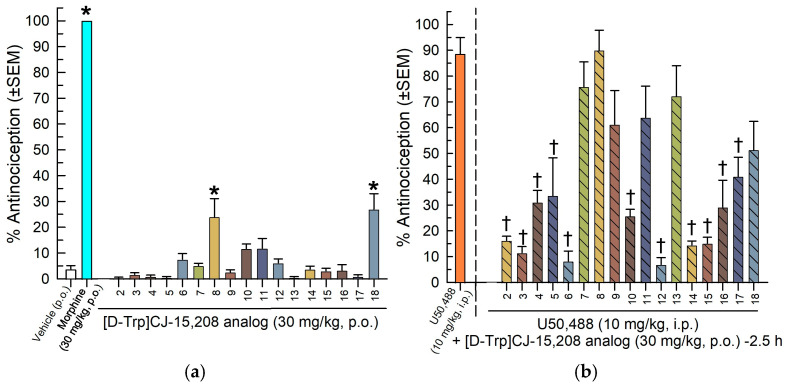
Screening of [D-Trp]- and [D-Phe^4^]CJ-15,208 analogs **2**–**18** for antinociception and KOR antagonism following oral administration (30 mg/kg, p.o.). (**a**) [D-Trp]- and [D-Phe^4^]CJ-15,208 analogs were administered, and the mice were tested 30 min later for antinociception in the 55 °C WWTW assay. Vehicle alone (10% DMSO/10% Solutol/80% saline, p.o.; left-most bar) was tested as a negative control, and morphine (30 mg/kg, p.o.) was tested as a positive control. (**b**) Two and a half hours later the KOR agonist U50,488 (10 mg/kg, i.p.) was administered, and mice were tested again in the 55 °C WWTW assay to evaluate [D-Trp]- and [D-Phe^4^]CJ-15,208 analog-mediated antagonism of U50,488-mediated antinociception. Points represent average % antinociception ± SEM from eight mice for each bar (*n* = 12 for U50,488). * Significantly different from vehicle treatment group; † significantly different from U50,488 treatment group (*p* < 0.05, one-way ANOVA with Dunnett’s multiple comparisons post hoc test).

Given our goal to identify compounds active after oral administration, we screened the 17 [D-Trp]- and [D-Phe^4^]CJ-15,208 analogs for antinociceptive and KOR antagonist effects following oral administration (30 mg/kg, p.o.). Thirty minutes after oral administration only morphine (30 mg/kg, p.o.) and two analogs (**8** and **18**) displayed significant antinociception compared to the vehicle (F_(18,133)_ = 81.6, *p* < 0.05, one-way ANOVA with Dunnett’s post hoc test; Figure 3a). Consistent with the initial i.c.v. screening results, the majority (11) of the analogs exhibited significant KOR antagonism by reducing KOR agonist-induced antinociception after oral administration (F_(17,130)_ = 11.0, *p* < 0.0001, one-way ANOVA with Dunnett’s post hoc test; Figure 3b). Analog **9** demonstrated robust KOR antagonist activity after i.c.v. administration (Figure 2b), and analogs **3**, **4**, **6** and **14** demonstrated robust KOR antagonist activity when screened after oral administration (Figure 3b), so these five compounds were selected for further characterization.

##### Characterization of the [D-Trp]- and [D-Phe^4^]CJ-15,208 Analogs **3**, **4**, **6**, **9** and **14**

**Figure 4:**
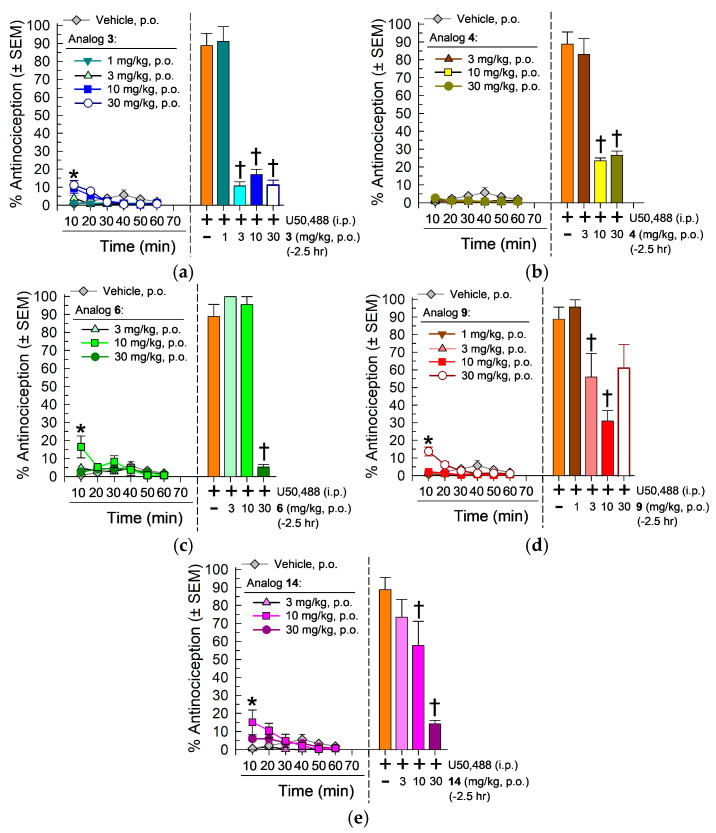
Antinociception and KOR antagonism in the 55 °C WWTW assay following p.o. administration of [D-Trp]CJ-15,208 analogs (**a**) **3**, (**b**) **4**, (**c**) **6**, (**d**) **9** or (**e**) **14**. Methods and antinociception are as described in Section 4.6. Points and bars represent average % antinociception ± SEM from 7 to 8 mice (U50,488 alone, orange bar = 16 mice). Data right of the dashed vertical line denotes testing of KOR antagonism against U50,488 antinociception in the same (color matched) mice as KOR antagonism evaluation, 2.5 h after treatment with the listed macrocyclic tetrapeptide. * Significantly different from vehicle treatment group, ANOVA with Tukey’s post hoc test; ^†^ significantly different from U50,488, (10 mg/kg, i.p., *p* < 0.05; one-way ANOVA Dunnett’s multiple comparisons post hoc test).

Analogs [D-Trp(formamide)]- (**3**), [D-Phe^4^]- (**4**), [D-Phe(*m*-F)^4^]- (**6**), [D-Phe(3,5-F_2_)^4^]- (**9**) and [D-Phe(*m*-Me)^4^]CJ-15,208 (**14**) were further characterized following oral (p.o.) administration. Each analog produced very modest (<20%) antinociception, significantly different from the vehicle only at the first 10 min after treatment with high doses (Figure 4). In contrast, 2.5 h pretreatment produced a dose-dependent antagonism by each analog against peripherally administered U50,488 (10 mg/kg, i.p.). Analog **3** significantly antagonized U50,488-mediated antinociception at doses of 3, 10 or 30 mg/kg, p.o. (F_(4,43)_ = 50.5, *p* < 0.0001, one-way ANOVA with Dunnett’s post hoc; Figure 4a, right panel) with an antagonist AD_50_ (and 95% CI) of 2.34 (1.13–3.78) mg/kg, p.o.), whereas analog **4** significantly antagonized U50,488 at doses of 10 and 30 mg/kg, p.o. (F_(3,35)_ = 27.6, *p* < 0.0001, one-way RM ANOVA with Dunnett’s post hoc test; Figure 4b, right panel), with an AD_50_ (and 95% C.I.) of 7.67 (5.03–9.62) mg/kg, p.o. In contrast, analog **6** produced significant antagonism of U50,488 only at a dose of 30 mg/kg, p.o. (F_(3,36)_ = 13.6, *p* < 0.0001, one-way RM ANOVA with Dunnett’s post hoc test; Figure 4c). A 2.5 h pretreatment with select doses of analog **14** (10 and 30 mg/kg, p.o.) significantly antagonized U50,488 antinociception (F_(3,36)_ = 14.3, *p* < 0.0001, one-way ANOVA with Dunnett’s post hoc; Figure 4e). Of interest, oral administration of analog **9** displayed significant dose-dependent antagonism of U50,488 antinociception after doses of 3 and 10, but not 30, mg/kg, p.o. (F_(4,43)_ = 8.38, *p* < 0.0001, one-way ANOVA with Dunnett’s post hoc; Figure 4d right panel). The reason for the decreased antagonism at the higher dose is unclear.

**Figure 5:**
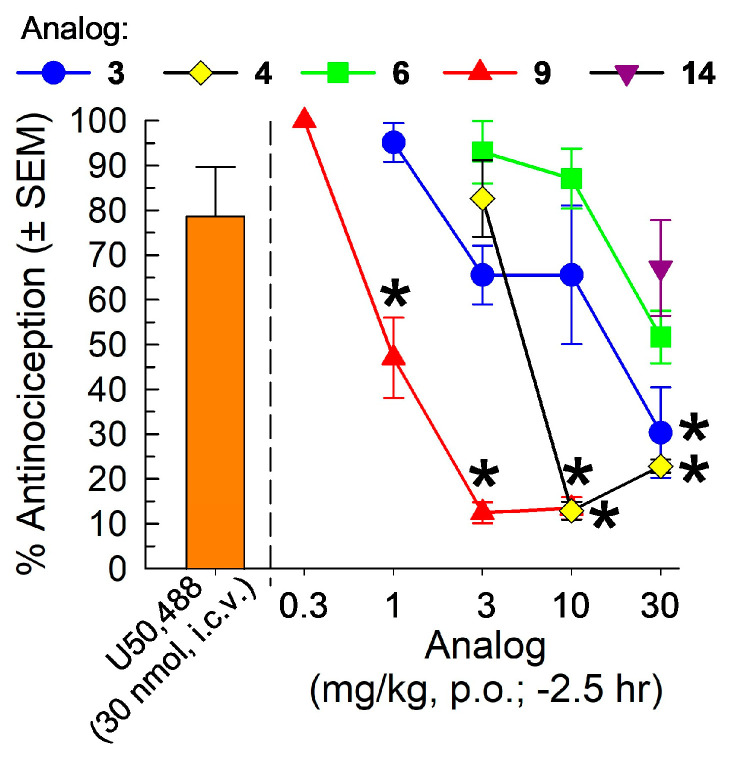
Orally administered analogs of [D-Trp]- and [D-Phe^4^]CJ-15,208 **3**, **4**, **6**, **9** and **14** cross the blood–brain barrier to differentially antagonize U50,488-induced antinociception in the mouse 55 °C WWTW test. Antinociception induced by centrally administered U50,488 (30 nmol, i.c.v.) was antagonized in mice first peripherally pretreated for 2.5 h (0.3-30 mg/kg, p.o.) with analogs **3** (blue line), **4** (yellow line) and **9** (red line), but not **6** (green line) or **14** (purple). Tail-withdrawal latencies were measured 30 min after injection of U50,488. Mean % antinociception ± SEM from seven to eight mice for each group is presented. * Significantly different from U50,488-induced antinociception alone; one-way ANOVA Dunnett’s multiple comparisons post hoc test.

The ability of the five [D-Trp]- and [D-Phe^4^]CJ-15,208 analogs to antagonize the antinociceptive effect of U50,488 administered centrally (30 nmol, i.c.v) was assessed following a 2.5 h pretreatment with the analogs (Figure 5); four of the analogs were tested at 30 mg/kg, p.o., while **9** was tested at 10 mg/kg, p.o. The compounds produced differing degrees of antagonism of CNS KORs, with significant antagonism observed with analogs **3**, **4** and **9** (F_(5,42)_ = 10.8, *p* = 0.0004, one-way ANOVA with Dunnett’s post hoc; Figure 5). In contrast, analogs **6** and **14** proved ineffective at 30 mg/kg, p.o. (*p* = 0.08 and 0.74, respectively). Furthermore, antagonism of CNS KORs by the three effective analogs was dose-dependent, with significant antagonism only after pretreatment with 30 mg/kg, p.o. of **3** (F_(4,34)_ = 5.44, *p* = 0.002; blue line, Figure 5) and 10 or 30 mg/kg, p.o. of **4** (F_(3,28)_ = 27.2, *p* < 0.0001, one-way ANOVA with Dunnett’s multiple comparisons post hoc test; yellow line, Figure 5); doses of 1–10 mg/kg, p.o. of **3** and 3 mg/kg, p.o. of **4** did not produce significant antagonism. Oral treatment with analog **4** antagonized i.c.v. U50,488 with an antagonist AD_50_ (and 95% C.I.) of 6.60 (3.81–9.62) mg/kg, p.o. Surprisingly, analog **9** proved the most efficacious antagonist of CNS KORs, with significant effects after pretreatment with doses of 1, 3 and 10 mg/kg, p.o. (F_(4,35)_ = 36.3, *p* < 0.0001, one-way ANOVA with Dunnett’s multiple comparisons post hoc test; Figure 5), and an antagonist AD_50_ (and 95% CI) of 1.33 (0.98–1.75) mg/kg, p.o.

**Figure 6:**
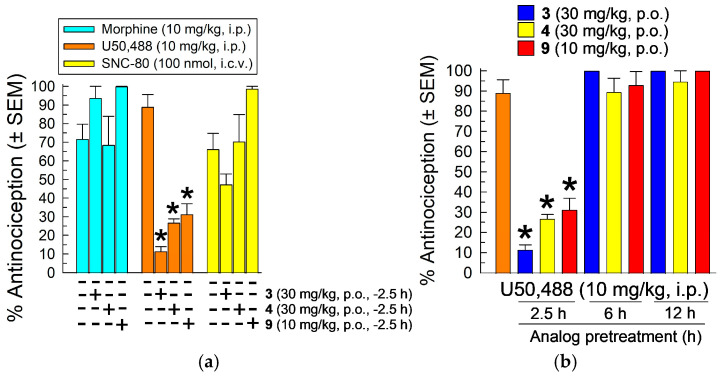
Characterization of analogs **3**, **4** and **9** opioid antagonist activity in the 55 °C warm-water tail-withdrawal assay. (**a**) A 2.5 h pretreatment with analog **3** or **4** (30 mg/kg, p.o.) or **9** (10 mg/kg, p.o.) prevented U50,488-induced antinociception (10 mg/kg, i.p.), but not that of morphine (10 mg/kg, i.p.) or SNC-80 (100 nmol, i.c.v.). (**b**) Time-dependent KOR antagonism by analogs **3**, **4** and **9**. The antinociceptive effect of U50,488 (10 mg/kg, i.p.; orange bar) was determined in mice pretreated for 2.5, 6 or 12 h with analog **3** or **4** (30 mg/kg, p.o.) or **9** (10 mg/kg, p.o.). Methods and antinociception are as described in Section 4.6. Points represent average % antinociception ± SEM from 7 to 16 mice for each bar (*n* = 5 for **9**-treated mice + SNC-80). * Significantly different from response of agonist alone (*p* < 0.05); (**a**) two-way ANOVA with Sidak’s multiple comparison or (**b**) one-way ANOVA with Dunnett’s post hoc tests.

The KOR selectivity and duration of antagonism was assessed after oral administration of analogs **3**, **4** or **9**. Analogs **3** or **4** (30 mg/kg, p.o.) or **9** (10 mg/kg, p.o.) administered 2.5 h prior to treatment with one of three opioid receptor-selective agonists demonstrated significant antagonism of only the KOR-selective agonist U50,488 (**3**: F_(2,66)_ = 17.8, *p* < 0.0001, **4**: F_(2,65)_ = 6.10 *p* = 0.004 and **9**: F_(2,63)_ = 17.5, *p* < 0.0001, two-way ANOVA with Sidak’s multiple comparison post hoc test; Figure 6a). Pretreatment with the analogs had no significant effect on the antinociceptive effects of the MOR-preferring agonist morphine (*p* = 0.07 or greater) or the delta opioid receptor (DOR)-selective agonist SNC80 (*p* = 0.07 or greater; Figure 6a, left and rightmost bars, respectively). The duration of significant KOR antagonism lasted at least 2.5 h, but less than 6 h, after treatment with a single dose of analog **3** (30 mg/kg, p.o.; F_(3,36)_ = 49.1, *p* < 0.0001), **4** (30 mg/kg, p.o.; F_(3,35)_ = 18.2, *p* < 0.0001) or **9** (10 mg/kg, p.o.; F_(3,36)_ = 20.0, *p* < 0.0001, one-way ANOVA with Dunnett’s post hoc test; Figure 6b).

#### 2.4.2. Evaluation of [D-Trp(formamide)]-, **3**, [D-Phe)^4^]-, **4**, and [D-Phe(3,5-F_2_)^4^]CJ-15,208, **9**, for Prevention of Stress-Induced Reinstatement of Extinguished Cocaine-Seeking Behavior in the Conditioned Place Preference (CPP) Assay

**Figure 7:**
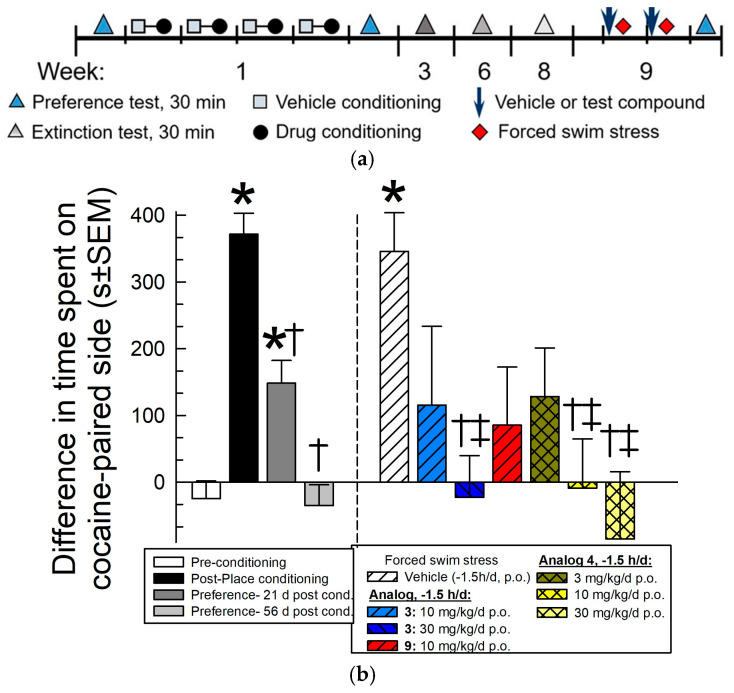
Prevention of stress-induced reinstatement of extinguished cocaine CPP by analogs **3** and **9**. (**a**) Reinstatement paradigm schematic. (**b**) Following 4 days of cocaine administration (10 mg/kg, s.c. daily), mice exhibited significant preference for the cocaine-paired environment, with extinction occurring 9 weeks later. Mice were then exposed to forced swim stress, reinstating preference. Pretreatment with **3** (10 or 30 mg/kg, p.o.), **4** (3, 10 or 30 mg/kg, p.o.) or **9** (30 mg/kg, p.o.) reduced stress-induced reinstatement of place preference when administered 90 min prior to FSS exposure. Bars represent means of *n* = 14–24 mice (with 23 vehicle-treated, FSS-exposed mice); cocaine place conditioning data in the left panel represents the combined responses of all 144 mice. * Significantly different from preconditioning place preference response; † significantly different from post-conditioning place preference response; ‡ significantly different from vehicle pretreatment reinstatement of place preference response (*p* < 0.05), one-way ANOVA followed by Tukey’s multiple comparison post hoc test.

Conditioned place preference is a robust, validated means to rapidly assess potential rewarding or aversive effects of opioids and is a well-established model in mice, where drugs like morphine with abuse liability produce CPP [21]. We have used this assay to demonstrate the ability of several peptides with antagonist activity at the KOR to prevent stress-induced reinstatement of cocaine-seeking behavior [6,7,8,9,10,11]. Analogs **3**, **4** and **9** were tested for their ability to prevent stress-induced reinstatement of cocaine-seeking behavior (Figure 7). Following 4 days of cocaine place conditioning, mice demonstrated a significant preference for the cocaine-paired chamber (F_(3,572)_ = 38.1, *p* < 0.0001, one-way ANOVA with Tukey’s multiple comparisons post hoc test; Figure 7b, black bar). Extinction of this preference was observed after repeated preference testing over 9 weeks after conditioning (*p* = 0.995 vs. pre-conditioning response, and *p* < 0.0001 vs. post-conditioning response, Tukey’s post hoc; Figure 7b, light gray bar). Mice were then pretreated once daily for two days with vehicle (10% DMSO/10% Solutol/80% saline, p.o.), analog **3** (10 or 30 mg/kg, p.o.), **4** (3, 10 or 30 mg/kg, p.o.) or analog **9** (10 mg/kg, p.o.) and exposed to forced swim stress (FSS; see reinstatement schematic, Figure 7a). Mice pretreated with the vehicle displayed significant reinstatement of drug-seeking behavior after exposure to forced swimming (F_(9,566)_ = 15.2, *p* < 0.0001, one-way ANOVA with Tukey’s post hoc; Figure 7b, white bars). When pretreated 1.5 h prior to FSS, analog **3** dose-dependently prevented stress-induced reinstatement with a 30 mg/kg dose (*p* = 0.003, Tukey’s post hoc test), but not a 10 mg/kg dose (*p*=0.52; Tukey’s post hoc test; Figure 7b, blue bars). Likewise, analog **4** dose-dependently prevented stress-induced reinstatement with a 10 or 30 mg/kg dose (*p* = 0.03 and 0.003, respectively, Tukey’s post hoc test; Figure 7b, yellow bars), but not with a 3 mg/kg dose (*p*=0.74; Tukey’s post hoc test; Figure 7b, olive green bar). Pretreatment with analog **9** appeared to reduce stress-induced reinstatement of cocaine CPP, but this was not significant (*p* = 0.53, Tukey’s post hoc test; Figure 7, red bar).

## 3. Discussion

### 3.1. In Vitro Pharmacological Results

The results from the in vitro assays demonstrate that there is significant toleration by the KOR for modification to the D-amino acid in position 4 of [D-Trp]CJ-15,208. Confirming the results we obtained for [D-Bta ^4^]CJ-15,208 [14] (see above), the indole NH of the D-Trp residue is not required for KOR interaction, although the identity of the substitution on this nitrogen influences KOR affinity, selectivity and antagonist potency. While both methyl and formamide substitutions on the D-Trp indole nitrogen were tolerated by the KOR, they had opposite effects on MOR vs. KOR affinities. The formamide derivative **3** exhibited the highest affinity and selectivity for the KOR among the analogs, with a KOR affinity approaching that of aticaprant and KOR selectivity equal to that of navacaprant. The formamide derivative also exhibited the highest KOR antagonist potency (IC_50_ = 49 nM) among the analogs tested. In contrast, the methyl-substituted derivative **2** exhibited decreased KOR affinity, minimal selectivity for the KOR vs. the MOR and 3-fold lower KOR antagonist potency (IC_50_ = 220 nM) compared to the parent [D-Trp]CJ-15,208 (IC_50_ = 66 nM).

[D-Phe^4^]CJ-15,208, **4**, exhibited 3.5-lower KOR affinity than [D-Trp]CJ-15,208 but comparable affinity to that of navacaprant. A variety of substitutions (fluorine in different positions, along with *m*- and *p*-methyl and *p*-nitro) were tolerated on the phenyl ring of D-Phe^4^ by the KOR. 3,4-Difluoro or *p*-nitro substitution on the phenyl ring increased KOR affinity, resulting in analogs with affinity identical to that of [D-Trp]CJ-15,208. Only a limited number of modifications (notably the *p*-aniline (analog **17**), but also the pentafluoro (analog **12**), *p*-methoxy (analog **18**) and *o*-methyl (analog **13**) groups) substantially decreased KOR affinity. The substitutions did not increase the KOR selectivity of the analogs, however (and in some cases decreased selectivity). While the substitutions increased KOR antagonist potency compared to [D-Phe^4^]CJ-15,208 (IC_50_ = 160–340 nM vs. 460 nM) these analogs were less potent antagonists than [D-Trp]CJ-15,208.

Incorporation of fluorine and other substitutions into the phenyl ring of a Phe residue has also been examined in peptides that preferentially bind to the DOR or MOR, with the effects of the substitution varying depending on the peptide. *p*-Fluoro substitution on Phe^4^ in *cyclo*[D-Pen^2^,D-Pen^5^] (DPDPE) was well tolerated by the DOR, increasing affinity, potency in a functional assay (the mouse vas deferens) and selectivity for the DOR [22]. In cyclic tetrapeptide analogs Tyr-*cyclo*[D-Cys-Phe(X)-D-Pen)OH related to JOM-13 *o*-, *m*- or *p*-fluoro substitutions were tolerated by the DOR, although with decreased DOR selectivity [23]. *m*-Fluoro substitution in Phe^3^ of deltorphin I was also tolerated by the DOR, while reports on the effect of *p*-fluoro substitution in this peptide are mixed [24,25]. In cyclic endomorphin 2 analogs, *p*- or 2,4-fluoro substitution was tolerated by the MOR in the phenyl ring of either Phe^3^ or Phe^4^ [26]. Similar to our present findings for the macrocyclic tetrapeptide, pentafluorphenylalanine was not tolerated in deltorphin I by the DOR [25]. *p*-Methyl substitution of Phe^3^ had different effects on DOR peptides; while this substitution increased affinity in the JOM13 cyclic tetrapeptide [23] it was not tolerated in deltorphin 1 [25]. In cyclic endomorphin 2 analogs *o*-, *m*- or *p*-methyl substitution on the phenyl ring of either Phe was tolerated by MOR, although the analogs exhibited decreased MOR selectivity [27]. The effect of *p*-nitro substitution of the phenyl ring of Phe in DOR-selective peptides varied depending on the peptide. In the Phe^3^ residue of the JOM-13 cyclic tetrapeptide, it increased DOR affinity but decreased selectivity [23], while this substitution in the Phe^3^ residue of deltorphin 1 resulted in loss of DOR affinity [24]. In [L-Ala^2^]DPDPE the Phe(*p*-NO_2_)^4^ analog exhibited similar affinity and selectivity in binding assays and increased potency in the mouse vas deferens assay compared to the parent peptide [28]. *p*-Amino substitution in the Phe^3^ residue of deltorphin I resulted in loss of DOR affinity and selectivity [24]; in endomorphin 2 incorporation into Phe^3^ resulted in a larger decrease in MOR affinity (44-fold decrease relative to endomorphin 2) compared to substitution in the Phe^4^ residue (9-fold decrease relative to endomorphin 2) [29].

### 3.2. In Vivo Pharmacological Results

In the initial in vivo screening the majority of the analogs antagonized peripherally administered U50,488 after both central (10 nmol, i.c.v., Figure 2b) and oral administration (30 mg/kg, p.o, Figure 3b), similar to the parent peptide [D-Trp]CJ-15,208 [9]. While some analogs produced significant antinociception after i.c.v. administration (Figure 2a), only two analogs, [D-Phe(3,4-difluoro)^4^]- (**8**) and [D-Tyr(Me)^4^]CJ-15,208 (**18**), exhibited significant antinociception following oral administration (Figure 3a). Five peptides, the D-Trp(formamide)] (**3**), D-Phe (**4**), D-Phe(*m*-F) (**6**), D-Phe(3,5-F_2_) (**9**) and D-Phe(*m*-Me) (**14**) analogs, were investigated further for their in vivo KOR antagonist potency (Figure 4 and Figure 5). As found in vitro, [D-Trp(formamide)]CJ-15,208, **3**, was the most potent analog as an antagonist of peripherally administered U50,488, while [D-Phe(*m*-F)^4^]CJ-15,208, **6**, was the least potent, significantly decreasing antinociception produced by peripherally administered U50,488 only at a dose of 30 mg/kg, p.o. (Figure 4). Interestingly, when U50,488 was administered centrally, [D-Phe(3,5,-difluoro)^4^]CJ-15,208, **9**, was the most potent, while neither the D-Phe(*m*-F)^4^, **6**, nor the D-Phe(*m*-Me)^4^ analog, **14**, significantly antagonized the antinociception (Figure 5). The differences observed in the antagonism of peripherally vs. centrally administered U50,488 are in contrast to other tetrapeptide analogs such as *cyclo*[Pro-Sar-Phe-D-Phe] [11], which retain the same activity profile across various routes of administration, with only diminished effects following peripheral administration. The differences in activity exhibited following the different routes of administration herein may be due to distribution, metabolism and/or bioavailability differences with the present analogs, further highlighting the influence of the blood–brain barrier on determining the potency of compounds with activity in the CNS. Similar to [D-Trp]CJ-15,208 [9] peptides **3, 4** and **9** selectively antagonized the KOR, and the KOR antagonism produced by these analogs was of short duration (Figure 6). Further analysis of the analogs to better understand structure–activity relationships for such properties was beyond the scope of this initial study but is a priority for future examination.

Given their oral activity and our goal to demonstrate the therapeutic potential of analogs that exhibit selective KOR antagonism, analogs **3**, **4** and **9** were assessed for their ability to prevent stress-induced reinstatement of cocaine-seeking behavior in the CPP assay (Figure 7). Analogs **3** and **4** produced dose-dependent reductions in reinstatement, albeit requiring higher doses than observed with their dose-dependent antagonism of centrally administered U50,488 (Figure 5). Differences in drug effects across behavioral assays are common, as we have reported earlier [7,8,9,10,11,14,30,31], especially those involving behavioral responses mediated by different systems such as the antinociceptive vs. reward responses, but may be captured by the dose–response studies as used here. Notably, the doses of **4** and **3** required for significant reductions in stress-induced reinstatement (10 and 30 mg/kg, p.o., respectively) are 6- and 2-fold lower than the 60 mg/kg, p.o. dose required for [D-Trp]CJ-15,208 [9], further showing compound-related differences expected in work of this type. Treatment with a 10 mg/kg, p.o. dose of analog **9**, however, did not cause a significant decrease in stress-induced reinstatement in mice (Figure 7), even though this dose is three times higher than the dose required to produce maximal antagonism of centrally administered U50,488 (Figure 5). This difference could possibly be due to differences in the distribution of the analog in different brain regions involved in antinociception vs. reward, although more detailed studies are needed to assess this beyond these initial studies. On a related note, to limit additional variables during novel compound characterization, only male mice were examined in this initial screening of the analogs. This is a modest limitation of this initial study, but given the well-established sex-related differences observed with dynorphin [32] and kappa opioid receptor activity [33,34,35,36], future investigation of promising analogs in female mice is warranted.

These studies continue our identification and characterization of selective peptide KOR antagonists that prevent stress-induced relapse to cocaine-seeking behavior [6,7,8,9,10,11,14,30], representing an important step in identifying peptide KOR-selective antagonists for advancement as potential treatments for substance abuse. The present testing focused on short-term treatment in one CUD model (cocaine conditioned place preference). We anticipate that future studies on potent next-generation analogs will utilize additional CUD models (e.g., self-administration). It will also be important to examine prolonged treatment with such KOR antagonists that further establish their potential application in the clinic as treatments for substance abuse.

Moreover, the potential application of KOR-selective antagonists as maintenance medications to promote abstinence is not limited to CUD. Stress is an established factor in relapse to the consumption of other addictive substances (e.g., opioids, methamphetamine, nicotine and alcohol) [37,38,39,40]. Notably, we have previously shown that pretreatment with another macrocyclic tetrapeptide KOR antagonist can prevent stress-induced relapse to opioid-seeking behavior, as well as decrease some symptoms of withdrawal in subjects physically dependent on morphine [31]. These data suggest further potential application of our peptide KOR antagonists in promoting abstinence from seeking a variety of reinforcing substances.

## 4. Materials and Methods

### 4.1. Chemicals

The sources of the reagents, amino acids, solid phase resin and solvents for peptide synthesis are the same as previously reported [10,13]. Amino acids are L-configuration unless otherwise specified, and abbreviations for amino acids follow the IUPAC-IUB join commission of biochemical nomenclature [41]. Standard Fmoc-protected amino acids, resin and coupling reagents were obtained from P3Bio (Louisville, KY, USA) and Chem-Impex (Wooddale, IL, USA); Fmoc-protected substituted D-Phe and D-Trp derivatives were obtained from ChemImpex. All other chemicals were obtained from Thermo Fisher Scientific (Waltham, MA, USA). Flash chromatography was performed on Teledyne RediSep Rf 40–60 µm silica gel cartridges.

All drugs used for in vivo testing were obtained from Sigma-Aldrich (Burlington, MA, USA).

### 4.2. Instruments

Electrospray ionization mass spectra were acquired on an Expression CMS-L (Advion, Ithaca, NY, USA). Reversed phase purification of selected peptides was performed on a Shimadzu semipreparative HPLC system (Columbia, MD, USA). Peptide purity was assessed by UHPLC analysis performed on a Shimadzu Nexera X2 UHPLC system, except for analog **4,** which underwent HPLC analysis using an Agilent 1260 HPLC system (Santa Clara, CA, USA).

### 4.3. Peptide Synthesis and Purification

The macrocyclic tetrapeptides were synthesized by a combination of solid phase synthesis of the linear precursors followed by cyclization in solution [13]. The linear peptide precursors D-aa-Phe-D-Pro-Phe (where D-aa = D-Trp(R) or D-Phe(R)) were synthesized on a 2-chlorotrityl chloride resin as previously described and cyclized using the general procedure described previously [10,13,14]. The linear peptide precursor (25 mM, 0.5 equiv) in *N,N*-dimethylformamide (DMF) was added dropwise at a rate of 1 mL/h to a solution of 0.75 equivalents of HATU (2-(1H-7-azabenzotriazol-1-yl)-1,1,3,3-tetramethyluronium hexafluorophosphate) and 4 equiv of *N,N*-diisopropylethylamine (DIEA) in DMF (resulting in a peptide concentration of 0.6 mM). Following addition of the peptide, a second portion of HATU (0.75 equiv) and 4 equivalents of DIEA were added directly to the reaction mixture, and additional linear peptide (25 mM, 0.5 equiv) in DMF was added dropwise at the same rate of 1 mL/h (final peptide concentration 1.2 mM). The reaction was then allowed to stir at room temperature for 6–10 h; if HPLC indicated the reaction was not complete, additional HATU and DIEA were added, and the reaction was allowed to proceed for an additional hour. The DMF was removed under reduced pressure, followed by addition of DCM and removal of the insoluble byproducts to give the crude cyclic peptide. Peptides **2**–**16** were purified by flash chromatography using a linear gradient of 10–80% EtOAc in hexanes over 15 min at a flow rate of 30 mL/min; following evaporation, the fractions were lyophilized from aq MeCN to obtain the pure cyclic peptides. The more hydrophilic peptides **17** and **18** were purified by reversed phase HPLC on a Vydac C18 column (10 μm, 22 × 250 mm) (Grace, Columbia, MD, USA) equipped with a Vydac C18 guard cartridge using a linear gradient of 30–70% MeCN in aqueous 0.1% TFA over 20 min at a flow rate of 20 mL/min, followed by lyophilization to obtain the pure peptides. Except where noted, the purified cyclic peptides were analyzed for purity on a Waters Acquity BEH C18 1.7 µm UHPLC column (2.1 × 50 mm) (Milford, MA, USA) at a flow rate of 0.2 mL/min using two orthogonal analytical HPLC solvent systems (MeCN and MeOH), with detection at 214 nm and the structure verified by ESI-MS; the UHPLC and MS data are shown in Table A2, and the chromatogram and mass spectra are included in the Supplementary Materials.

### 4.4. In Vitro Pharmacological Evaluation

#### 4.4.1. Opioid Receptor Binding Assays

Competition opioid binding experiments were performed as previously described [42]. Macrocyclic tetrapeptides were dissolved in DMSO at a concentration of 1 mM, then diluted to test concentrations in a final vehicle of 0.1% DMSO in 50 mM Tris-HCl, pH 7.4. Membranes from Chinese hamster ovary cells (CHO), stably expressing either the human KOR or MOR, were incubated with 11 different concentrations of peptide and radioligand in a final volume of 1 mL of 50 mM Tris-HCl, pH 7.4. For KOR and MOR binding, 0.4 nM [^3^H]U69,593 and 0.25 nM [^3^H]DAMGO (Revvity, Inc., Waltham, MA, USA) were used, respectively. Nonspecific binding for the KOR and MOR was measured by the inclusion of 10 μM naloxone. Samples were incubated for 60 min at 25 °C before being filtered through Whatman #32 glass fiber filters. Filters were counted in 2 mL of Ecoscint A scintillation fluid (National Diagnostics, Atlanta, GA, USA). For binding to the MOR, [^3^H]DAMGO (Revvity, Inc.) was used at a concentration of 0.25 nM with hMOR-CHO membranes.

The IC_50_ values were determined from a nonlinear regression analysis to the data using SigmaPlot. K_i_ values were calculated as previously described [43]. The K_d_ value for [^3^H]U69,593 binding to hKOR-CHO membranes was 0.409 nM. [^3^H]DAMGO had a K_d_ value of 0.556 nM for binding to hMOR-CHO membranes.

[D-Trp]-, [D-Phe^4^]CJ-15,208 and several analogs were screened at 10 µM for binding to the human DOR using 1 nM [^3^H]DPDPE. Consistent with our previous results [14], the peptides exhibited minimal interaction with the DOR; the maximum inhibition of binding by any of the peptides tested at 10 µM was 54 ± 4%.

#### 4.4.2. KOR cAMP Assay Using GloSensor-22F

##### Plasmid DNA Cloning

A *piggy-Bac (PB)* transposon/transposase system was utilized to allow for the stable integration of the GloSensor-22F cAMP sensor (Promega, Madison, WI, USA) gene into genomic TTAA sites in Chinese hamster ovary cells already stably expressing the hKOR. The pFastBac hyperPB plasmid vector, which codes for the *piggy-Bac* transposon/transposase system, was utilized and was a kind gift from Dr. John Lueck [44]. The multicistronic expression cassette developed for our experiments consisted of a CMV promoter driving constitutive expression of GloSensor-22F cAMP sensor, followed by an internal ribosome entry site (IRES) to drive the expression of codon-optimized HygR-P2A-mScarlet (hygromycin resistance gene and mScarlet separated by a 2A self-cleaving peptide element). To clone the expression cassette onto the pFastBac vector, the CMV promoter and GloSensor-22F were PCR amplified from pGloSensor-22F cAMP Plasmid (Promega), the IRES sequence was PCR amplified from pLVX-CMV-Galpha-oA ONE-GO (a gift from Dr. Mikel Garcia-Marcos, Addgene plasmid #189732), the HygR-P2A-mScarlet sequence was a kind gift from Dr. Lueck, and a bGH-poly(A) termination sequence was PCR amplified from pcDNA3.1(+). The pFastBac vector was linearized via overnight digestion with CspCI at 37 °C, which cuts between two inverted terminal repeat (ITR) sequences, and the corresponding backbone and all PCR fragments were gel purified and assembled via Gibson assembly using the Gibson Assembly MasterMix (New England Biolabs, Ipswich, MA, USA). The resulting pFastBac CMV GloSensor-22F IRES HygR-P2A-mScarlet bGH plasmid was transformed into NEB Stable Competent *E. coli* (New England Biolabs), miniprepped using the E.Z.N.A. Plasmid DNA Mini Kit (Omega Bio-Tek, Norcross, GA, USA), and sequence verified by nanopore whole-plasmid sequencing (Eurofins Genomics, Louisville, KY, USA).

##### Production of GloSensor-22F KOR CHO Cell Stable Lines (KOR-CHO-GLO)

3.0 × 10^6^ hKOR-CHO cells were plated on a 6 cm dish and incubated for four h at 37 °C and 5% CO_2_ in DMEM (Corning Mediatech, Manassas, VA, USA) supplemented with 5% FBS (Corning Mediatech), 1% Penicillin/Streptomycin (Gibco, Waltham, MA, USA). The cells were then transfected with 2.0 μg pFastBac CMV GloSensor-22F IRES HygR-P2A-mScarlet bGH using PEI as a transfection reagent. The following day, transfected cells were reseeded onto a 10 cm dish at a density of 5.0 × 10^5^ cells and 600 μg/mL hygromycin B (Gold Bio, St. Louis, MO, USA) was added to the culture media. Cells were cultured under selection antibiotics for seven days, and the selection process was monitored by the increase in abundance of mScarlet positive cells via epifluorescence microscopy.

##### Performing GloSensor cAMP Assay

Following the selection period, KOR-CHO-GLO cells were maintained at 37 °C and 5% CO_2_ in DMEM supplemented with 5% FBS, 1% Penicillin/Streptomycin and 300 μg/mL hygromycin B to reduce the likelihood of CMV promoter silencing. On the day of the assay, cells were detached with 0.05% trypsin (Gibco) and counted, then centrifuged (1000× *g*, 5 min) and resuspended in assay buffer (1xPBS, 0.1% glucose, 0.5 mM MgCl_2_) containing 4 mM D-luciferin substrate (Gold Bio) at approximately 2 million cells/mL. 25 μL of cells (~50,000/well) were seeded onto poly L-lysine-coated white 96-well flat bottom plates (Greiner Bio-One, Kremsmünster, Austria)) and returned to the incubator for 60 min. Following incubation, cells were allowed to equilibrate to room temperature protected from light (10–15 min). Initial luminescence readings (at least 10 cycles) were collected for each well using a FLUOstar Omega v.6.2 plate reader (BMG Labtech, Ortenberg, Germany), after which the plate was removed. Prepared 4x treatments of drug (25 μL) were added to appropriate wells followed by 50 μL of 20 μM forskolin (AmBeed, Buffalo Grove, IL, USA) before the plate was returned to the reader and luminescence measured until final plateau levels were achieved (20–30 min). Assays were performed in duplicate and repeated at least three times.

##### Data Analysis—Agonist Mode

Initial and final luminescence readings were determined for each well and mean readings calculated for each treatment. Ratios of mean final to mean initial luminescence were calculated. Delta ratios were calculated by subtracting the ratio for 10 μM U50,488, which produced maximum inhibition of forskolin-stimulated cAMP and represented the baseline ratio for the assay. Forskolin (10 μM) represented the maximum cAMP stimulation. Results are expressed as percent inhibition of forskolin-stimulated cAMP.

##### Data Analysis—Antagonist Mode

Antagonism was measured by including an approximate IC_80_ concentration of U50,488 (10 nM) in each compound treatment. Maximum inhibition and stimulation of cAMP were measured as in agonist mode. Delta ratios and percent of maximal stimulations were determined. Results are shown normalized to 10 nM U50,488 inhibition of cAMP.

### 4.5. Metabolic Stability in Liver Microsomes

Evaluation of the metabolic stability of the macrocyclic tetrapeptides by human and mouse liver microsomes was performed by Curia Global (Buffalo, NY, USA). Test compounds (1 µM) were incubated with liver microsomes (1 mg protein/mL) at 37 °C, and samples taken at 0, 5, 10, 15, 30 and 45 min analyzed by LC-MS/MS for disappearance of the parent peptide.

### 4.6. In Vivo Pharmacological Evaluation

#### 4.6.1. Animals

In vivo studies were performed with adult male C57BL/6J mice obtained from Jackson Labs (Bar Harbor, ME, USA). C57BL/6J mice are established and validated subjects in the tail-withdrawal [45,46] and place-conditioning [47] assays. All mice were used at an age of 7–11 weeks at the initiation of testing and were group housed in ventilated cages (maximum of five animals per cage) in a temperature-controlled, specific pathogen-free room kept on a 12 h light-dark cycle. Upon the completion of testing, all mice were euthanized by inhalation of carbon dioxide, followed by cervical dislocation as a secondary measure, as recommended by the American Veterinary Medical Association.

All animal studies were preapproved by the University of Florida (Gainesville, FL, USA) Institutional Animal Care and Use Committee, in accordance with the 2011 National Institute of Health Guide for the Care and Use of Laboratory Animals. All results of animal testing are reported in accordance with ARRIVE guidelines [48]. Initial sample sizes were approximated by Power analysis, with animals assigned randomly to groups. Drug treatment experiments were conducted in a blinded fashion.

#### 4.6.2. Peptide Solutions for In Vivo Pharmacological Testing

For intracerebroventricular administration, peptides were dissolved in DMSO at a concentration of 20 mM (100 nmol/5 µL), then diluted to test doses in a final vehicle solution of 50%DMSO/50% saline (0.9%). For all other testing (for oral administration), peptides were made fresh each day for testing in a final vehicle solution of 10% DMSO/10% Solutol/80% saline (0.9%).

#### 4.6.3. Injection Techniques

Intracerebroventricular injections were made directly into the lateral ventricle according to the modified steps detailed previously [29,42]. The volume of all i.c.v. injections was 5 μL, using a 10 μL Hamilton microliter syringe. The mouse was lightly anesthetized with isoflurane, an incision was made in the scalp, and the injection was made 2 mm lateral and 2 mm caudal to bregma at a depth of 3 mm.

#### 4.6.4. Antinociceptive Testing

The 55 °C warm-water tail-withdrawal assay was performed as previously described [18]. Warm (55 °C) water in a 1.5 L heated water bath was used as the thermal nociceptive stimulus, with the latency of the mouse to withdraw its tail from the water taken as the endpoint. After determining baseline tail-withdrawal latencies, mice were administered vehicle alone or a single graded dose of established opioids or [D-Trp]CJ-15,208 analogs through the i.c.v., i.p. or p.o. route. To determine antinociceptive activity, the tail-withdrawal latency was determined repeatedly every 10 min following administration of the compounds for 1 h or until latencies returned to baseline values. A cut-off time of 15 s was used in these studies; if the mouse failed to display a tail-withdrawal response during that time, the tail was removed from the water and the animal was assigned a maximal antinociceptive score of 100%. At each time point, antinociception was calculated according to the following formula:

% antinociception = 100 × (test latency − control latency)/(15 − control latency)

Initial screening of the [D-Trp]- and [D-Phe^4^]CJ-15,208 analogs in the 55 °C WWTW assay utilized i.c.v administration (10 nmol). This dose and route of administration was previously demonstrated to produce maximal antinociception for CJ-15,208 and KOR antagonism for both CJ-15,208 and [D-Trp]CJ-15,208 [8]. [D-Trp]- and [D-Phe^4^]CJ-15,208 analogs were further tested following oral administration (30 mg/kg, p.o.) at this dose. [D-Trp]CJ-15,208 demonstrated robust, measurable KOR antagonism [9] without solubility issues. The dose response of analogs **3**, **6**, **9** and **14** were also assessed following administration of one of up to four additional oral (p.o.) doses (1–30 mg/kg). The results of the initial screening of several of the analogs (**2**, **3**, **5–8** and **12–17**) were included in figures in a previous publication [31].

To screen for KOR antagonist activity, 2.5 h after administration of a [D-Trp]- and [D-Phe^4^]CJ-15,208 analog (10 nmol, i.c.v. or 30 mg/kg, p.o.), the KOR-selective agonist U50,488 (10 mg/kg, i.p.) was administered and the compounds were evaluated for their ability to attenuate U50,488-mediated antinociception. Additional testing after administering U50,488 through the i.c.v. route (30 nmol) assessed the ability of analogs to produce CNS KOR antagonism. The dose response of KOR antagonism produced by selected analogs was determined by evaluating antinociception after administration of one of up to three additional p.o. doses. The opioid receptor antagonist selectivity of analogs **3** and **9** were further evaluated after administration of a single p.o. dose of the peptide 2.5 h prior to the MOR-preferring agonist morphine (10 mg/kg, i.p.) or the DOR-selective agonist SNC-80 (100 nmol, i.c.v.). Antinociception was measured 40 min after peripheral (i.p.) administration or central administration (i.c.v.) of the opioid receptor-selective agonist in order to capture optimal activity specific to the route of administration. The duration of KOR antagonism produced by analogs **3** (30 mg/kg, p.o.) or **9** (10 mg/kg, p.o.) was evaluated by pretreating mice 2.5, 6 or 12 h prior to administration of U50,488 (10 mg/kg, i.p.) and antinociception testing.

#### 4.6.5. Place Conditioning to Determine Conditioned Place Preference or Aversion

An automated, balanced three-compartment place conditioning apparatus (San Diego Instruments, San Diego, CA, USA) and a 2- or 4-day counterbalanced place conditioning design was used, similar to methods previously described [9]. The amount of time subjects spent in each of the three compartments was measured over a 30 min testing period. Prior to place conditioning, an initial preference test was performed, in which the animals could freely explore all open compartments.

To perform place conditioning using a counterbalanced design, mice were administered 0.9% saline and consistently confined in a randomly assigned outer compartment for 40 min, with half of each group in the right chamber and half in the left chamber. Mice were place conditioned for 4 days with cocaine (10 mg/kg, i.p.) for use in reinstatement testing, as described previously [8]. Data are plotted as the difference in time spent in the conditioning drug-paired and vehicle-paired compartments. Accordingly, a positive value reflects a conditioned preference for the drug-paired side, while a negative value reflects conditioned place aversion.

#### 4.6.6. Extinction Testing

Preference tests were performed twice weekly with cocaine-conditioned mice until extinction was established. Extinction is defined as a statistically significant decrease in the time spent in the drug-paired compartment during the extinction trial as compared to the post-conditioning response after the initial 4 days of cocaine place conditioning.

#### 4.6.7. Reinstatement Testing

Following extinction, reinstatement of cocaine CPP was examined after exposure to forced swim stress, as described previously [8,30]. Mice were pretreated daily with the vehicle, analog **3** (10 or 30 mg/kg, p.o.) or analog 9 (10 mg/kg, p.o.) 90 min prior to exposure to a forced swim stress for 2d, used to produce stress-induced reinstatement of extinguished CPP. During FSS, mice were placed in a 5 L beaker (40 cm tall × 25 cm in diameter) filled with 3.5 L of approximately 30 °C water to swim without the opportunity to escape. After each trial, mice were removed from the water, towel dried and placed back in their home cages for at least 7 min before additional testing. On the first day, animals were subjected to a single 15 min trial of forced swimming. On the following day, animals were exposed to a series of four swimming sessions that were 6 min long, separated by 7–12 min rest periods spent in their home cage. On the day following the completion of stress exposure, mice were tested for place preference to determine possible reinstatement.

### 4.7. Statistical Analysis

All dose–response lines were analyzed by regression, and AD50 (dose antagonizing agonist-induced antinociception by 50%) values and 95% C.I. were determined using individual data points from graded dose–response curves with Prism 10.0 software (GraphPad, La Jolla, CA, USA). Percent antinociception was used to determine within-group effects and to allow comparison to baseline latency in tail-withdrawal experiments. Significant differences in behavioral data were analyzed by ANOVA (one-way or two-way with repeated measures (RMs), as appropriate). Significant results were further analyzed with Dunnett’s, Tukey’s and Sidak’s multiple comparison post hoc tests, as appropriate. Data for conditioned place preference experiments were analyzed by one-way RM ANOVA, with analyses examining the main effect of conditioned place preference phase (e.g., pre- or post-conditioning) and the interaction of drug pretreatment and/or exposure. All data are presented as mean ± SEM, with significance set at *p* < 0.05.

## 5. Conclusions

A number of modifications to D-Trp or D-Phe^4^ were tolerated by the KOR, including substitution on the D-Trp indole nitrogen and incorporation of one or more fluorines and other substitutions on the phenyl ring in D-Phe. In vivo most of the resulting analogs exhibited KOR antagonist activity after oral administration. [D-Trp(formamide)]-, **3**, [D-Phe^4^]-, **4**, and [D-Phe(3,5-F_2_)^4^]CJ-15,208, **9**, dose-dependently antagonized centrally administered U50,488, and peptides **3** and **4** prevented stress-induced reinstatement of stress-induced cocaine CPP at doses of 10 and 30 mg/kg p.o., respectively, doses that are 2- to 6-fold lower than that required for prevention of stress-induced reinstatement by [D-Trp]CJ-15,208 [9].

These results demonstrate the promise of these macrocyclic peptides as potential treatments for cocaine use disorder. Our exploration of additional structural modifications to these macrocyclic peptides to further enhance their in vivo potency is ongoing, using the SAR results for the D-Trp residue described here in the design of the next generation of analogs.

## 6. Patents

Patent applications have been filed on the peptides.

## Figures and Tables

**Table 1:** In vitro pharmacological evaluation of the macrocyclic tetrapeptides for their affinities for human KOR and MOR, and their KOR efficacies and antagonist potencies in the cAMP assay. Data for the small molecule KOR antagonists aticaprant and navacaprant are included for comparison.

	KOR K_i_	MOR K_i_		KOR	
Analog ^1^	(nM ± S.E.M) ^2^	(nM ± S.E.M) ^2^	Selectivity ^3^	Efficacy (%) ^4^	Antag. IC_50_^5^
[D-Trp]CJ-15,208 and its analogs:					
**1**	6.9 ± 1.1	58 ± 7.5	8	16 ± 2.4	66 ± 11
**2**, D-Trp(Me)	16 ± 0.8	36 ± 2.6	2	14 ± 7.1	220 ± 32
**3**, D-Trp(form-amide)	2.2 ± 0.15	90 ± 14	41	9.2 ± 4.9	49 ± 5.4
[D-Phe(X)^4^]CJ-15,208:					
**4**, X = H	24 ± 0.97	270 ± 23	11	19 ± 8.1	460 ± 100
**5**, X = *o*-F	39 ± 0.57	350 ± 22	9	ND ^6^	ND
**6**, X = *m*-F	32 ± 5.8	160 ± 15	5	11 ± 5.6	340 ± 58
**7**, X = *p*-F	15 ± 1.8	45 ± 1.7	3	5.5 ± 7.9	170 ± 13
**8**, X = 3,4-F_2_	6.6 ± 0.73	57 ± 5.6	9	13 ± 2.7	170 ± 28
**9**, X = 3,5-F_2_	25 ± 4.3	290 ± 27	12	14 ± 2.4	320 ± 49
**10**, X = 2,4,5-F_3_	27 ± 3.3	270 ± 30	10	19 ± 6.8	290 ± 40
**11**, X = 3,4,5-F_3_	12 ± 0.44	46 ± 5.4	4	2.2 ± 6.2	160 ± 19
**12**, X = F_5_	310 ± 9.9	ND	ND	ND	ND
**13**, X = *o*-Me	110 ± 13	280 ± 8	2.5	ND	ND
**14**, X = *m*-Me	26 ± 3.0	130 ± 11	5	0.74 ± 5.7	320 ± 67
**15**, X = *p*-Me	11 ± 0.67	110 ± 15	10	18 ± 1.4	190 ± 17
**16**, X = *p*-NO_2_	7.0 ± 0.99	61 ± 2.7	9	7.3 ± 7.3	210 ± 13
**17**, X = *p*-NH_2_	^7^	ND	ND	ND	ND
**18**, X = *p*-OMe	150 ± 16	ND	ND	ND	ND
Aticaprant	0.77 ± 0.10	15 ± 2.0	19	−48 ± 3.0 ^8^	8.0 ± 1.7
Navacaprant	21 ± 3.1	850 ± 73	40	7.4 ± 7.5	69 ± 17

^1^ Substitution for D-Trp; ^2^ values from three independent experiments; ^3^ MOR K_i_/KOR K_i_; ^4^ mean % inhibition ± SEM at 10 µM relative to the KOR agonist U50,488 in the cAMP assay; data from three independent experiments performed in triplicate; ^5^ IC_50_ value (nM ± SEM) for antagonism of an IC_80_ concentration (10 nM) of U50,488 in the cAMP assay; data from three independent experiments performed in duplicate; ^6^ Not determined; ^7^ 65 ± 2.8% inhibition at 10 μM. ND = not determined; ^8^ aticaprant exhibited inverse agonist activity at the KOR.

## Data Availability

Data will be made available by the corresponding authors upon request.

## Supplementary Materials

The following supporting information can be downloaded at https://www.mdpi.com/article/10.3390/molecules30193993/s1, UHPLC/HPLC chromatograms and mass spectra for analogs **2**–**18**.

## Abbreviations

The following abbreviations are used in this manuscript:

ANOVA	Analysis of variance
cAMP	Cyclic adenosine monophosphate
CHO	Chinese hamster ovary
CI	Confidence interval
CJ-15,208	*cyclo*[Phe-D-Pro-Phe-Trp]
CPP	Conditioned place preference
CUD	Cocaine use disorder
[D-Phe^4^]CJ-15,208	*cyclo*[Phe-D-Pro-Phe-D-Phe]
[D-Trp]CJ-15,208	*cyclo*[Phe-D-Pro-Phe-D-Trp]
DAMGO	[D-Ala^2^,*N*-MPhe^4^,glyol]enkephalin
D-Bta	D-benzothienylalanine
DCM	Dichloromethane
DIEA	*N,N*-Diisopropylethylamine
DMF	*N,N*-Dimethylformamide
DMSO	Dimethyl sulfoxide
DOR	Delta opioid receptor
DPDPE	*cyclo*[D-Pen^2^,D-Pen^5^]enkephalin
FSS	Forced swim stress
HATU	2-(1H-7-Azabenzotriazol-1-yl)-1,1,3,3-tetramethyluronium hexafluorophosphate
HOBt	hydroxybenzotriazole
i.c.v.	intracerebroventricular
i.p.	intraperitoneal
ITR	Inverted terminal repeat
KOR	Kappa opioid receptor
MOR	Mu opioid receptor
ND	Not determined
p.o.	Per os
RM	Repeated measures
PyBOP	Benzotriazole-1yloxy-tris-pyrrolidinophophonium hexafluorphosphate
SAR	Structure–activity relationships
SEM	Standard error of the mean
SNC-80	(+)-4-[(αR)-α-((2S,5R)-4-Allyl-2,5-dimethyl-1-piperazinyl)-3-methoxybenzyl]-*N,N*-diethylbenzamide
TFA	Trifluoroacetic acid
U50,488	(±)-*trans*-3,4-Dichloro-*N*-methyl-*N*-[2-(1-pyrrolidinyl)cyclohexyl]benzeneacetamide
UHPLC	Ultrahigh performance liquid chromatography
WWTW	Warm-water tail withdrawal

## Appendix A

### Appendix A.1

**Table A1:** Stability of [D-Trp]- and [D-Phe^4^]CJ-15,208 and selected analogs in human (HLMs) and mouse liver microsomes (MLMs)^1.^ and kinetic aqueous solubility.

	HLM		MLM		Solubility ^2^
Analog ^3^	t_1/2_ (min)	CL_int_ ^4^	t_1/2_ (min)	CL_int_ ^4^	(µM)
**1**	5.5	127	2.6	269	23
**2,** D-Trp(Me)	2.4	286	3.3	211	12
**3**, D-Trp(COH)	2.8	244	1.1	607	4.6
[D-Phe(X)^4^]CJ-15,208:					
**4**, X = H	4.2	165	1.6	447	24
**5**, X = *o*-F	1.9	373	1.6	430	15
**6**, X = *m*-F	1.8	376	2.1	331	26
**7**, X = *p*-F	2.3	296	3.4	202	25
**8**, X = 3,4-F_2_	2.2	309	1.1	629	9.5
**9**, X = 3,5-F_2_	2.9	237	1.2	600	8.6
**10**, X = 2,4,5-F_3_	1.5	462	1.0	699	7.4
**11**, X = 3,4,5-F_2_	1.6	447	2.9	238	4.8
**14**, X = *m*-Me	4.1	170	1.5	467	19
**15**, X = *p*-Me	5.4	129	2.9	236	24
**16**, X = *p*-NO_2_	6.7	103	4.4	159	6.7

^1^ Metabolism by liver microsomes (1 mg protein/mL); ^2^ kinetic solubility determined in 1% DMSO, pH 7.4; ^3^ substitution for D-Trp; ^4^ µL/min/mg protein.

**Table A2:** Analytical data for the [D-Trp]- and [D-Phe^4^]CJ-15,208 analogs ^1^.

	UHPLC Syst. 1 ^2,3^	UHPLC Syst. 2 ^4^	ESI (*m/z* ^5^)	
Analog ^2^	t_1/2_ (min)	t_1/2_ (min)	Observed	Calc
**2**	8.05 ^2^	16.13	614.9	614.3
**3**	7.33 ^2^	14.94	628.9	628.3
**4**	5.71 ^6^	12.4 ^6^	539.1 ^7^, 561.3	539.1 ^7^, 561.3
**5**	9.89 ^3^	14.61	579.4	579.3
**6**	7.76 ^2^	15.19	579.9	579.3
**7**	7.67 ^2^	15.27	598.0	579.3
**8**	10.12 ^3^	15.49	575.2 ^7^, 597.6	575.2 ^7^, 597.2
**9**	10.25 ^3^	15.71	597.6	597.2
**10**	10.42 ^3^	16.13	593.2 ^7^, 615.6	593.2 ^7^, 615.2
**11**	10.59 ^3^	16.68	593.2 ^7^, 615.6	593.1 ^7^, 615.2
**12**	11.04 ^3^	17.58	629.4 ^7^, 651.3	629.2 ^7^, 651.2
**13**	8.13 ^2^	16.57	576.0	575.3
**14**	8.19 ^2^	16.57	576.0	575.3
**15**	8.14 ^2^	16.53	576.0	575.3
**16**	9.62 ^3^	14.01	606.3	606.2
**17**	5.07 ^3^	6.69	554.3 ^7^, 576.8	554.3 ^7^, 576.3
**18**	9.41 ^3^	13.83	569.5 ^7^, 591.5	569.3 ^7^, 591.3

^1^ UHPLC analysis was performed on a Waters Acquity BEH C18 column, 1.7 µm, 2.1 × 50 mm, at a flow rate of 0.2 mL/min except where otherwise noted; all peptides were >99% pure in both systems. Solvent A was A 0.1% aq TFA; solvent B was 0.1% TFA in MeCN for solvent system 1 and 0.1% TFA in MeOH for solvent system 2. The gradient was started after 1 min at the indicated initial solvent composition. ^2^ 40–90% solvent B over 10 min; ^3^ 30–80% solvent B over 10 min; ^4^ 50–90% solvent B over 20 min; ^5^ M+Na^+^ unless otherwise specified; ^6^ HPLC analysis performed on a Vydac C18 218TP 5 µ column (4.6 × 50 mm) at a flow rate of 1 mL/min. System 1 was 40–70% solvent B (MeCN with 0.1% TFA) over 30 min; System 2 was 50–80% solvent B (MeOH) over 30 min, with the gradient in both systems starting after 1 min; ^7^ M+H^+^.
