# Supplementary material for: Optimization of the Macrocyclic Tetrapeptide [D-Trp]CJ-15,208 to Prevent Stress-Induced Relapse of Cocaine-Seeking Behavior
Source: Molecules. 2025 Oct 5;30(19):3993. doi: 10.3390/molecules30193993 (PMC12526156; doi:10.3390/molecules30193993)

Supplementary Information
Optimization of the Macrocyclic Tetrapeptide [D-Trp]CJ-15,208 to Prevent Stress-Induced Relapse of Cocaine-Seeking Behavior

Jane V. Aldrich, Dmitry Yakolev, Jeremy S. Coleman, Sanjeewa N. Senadheera, Heather M. Stacy, Shainnel O. Eans, Brian I. Knapp, Jean M. Bidlack, and Jay P. McLaughlin

UHPLC/HPLC chromatograms and mass spectra of analogs **2-18**

2, [D-Trp(Me)]CJ-15,208
UHPLC, System 1

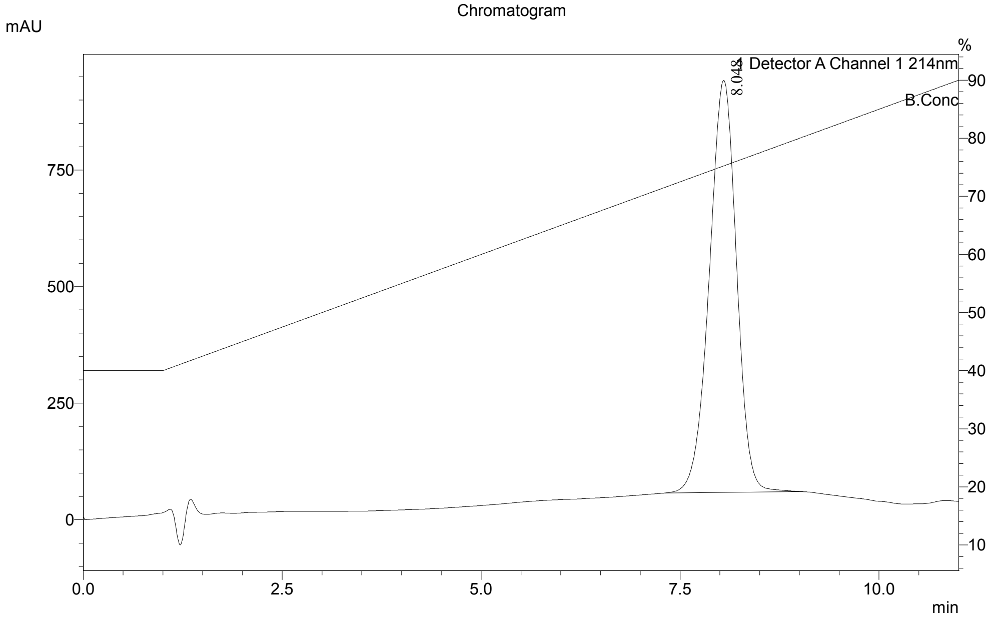

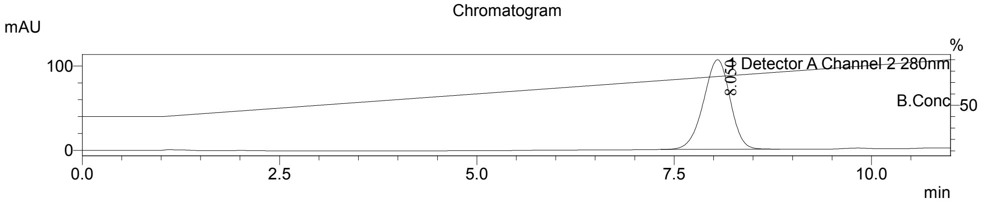

Peak Table
Detector A Channel 1 214nm

Peak#	Ret. Time	Area	Area%
1	8.048	21023087	100.000
Total		21023087	100.000

Peak Table
Detector A Channel 2 280nm

Peak#	Ret. Time	Area	Area%
1	8.050	2497676	100.000
Total		2497676	100.000

2, [D-Trp(Me)]CJ-15,208

UHPLC, System 2

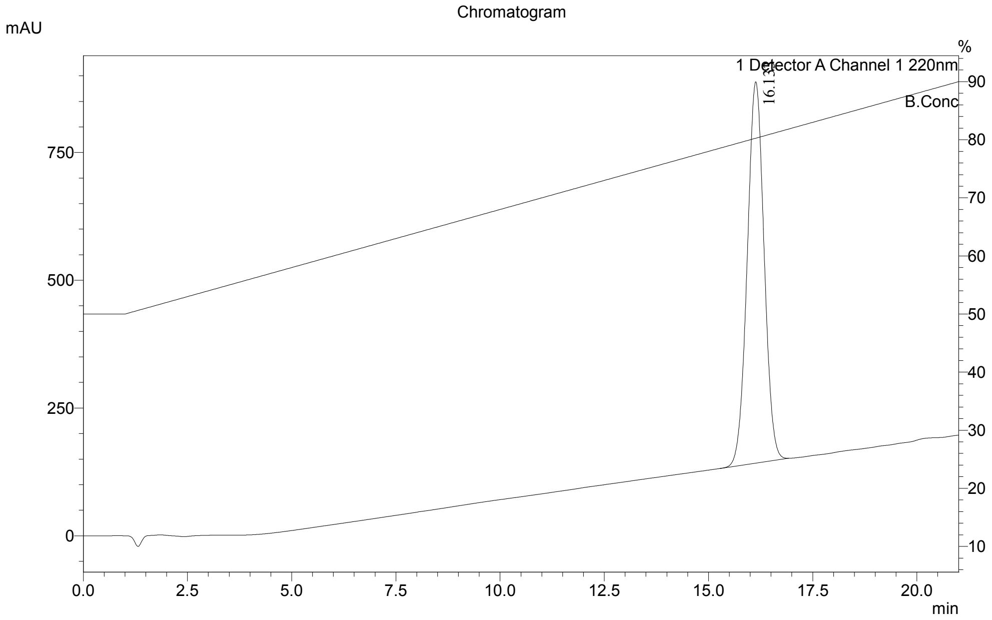

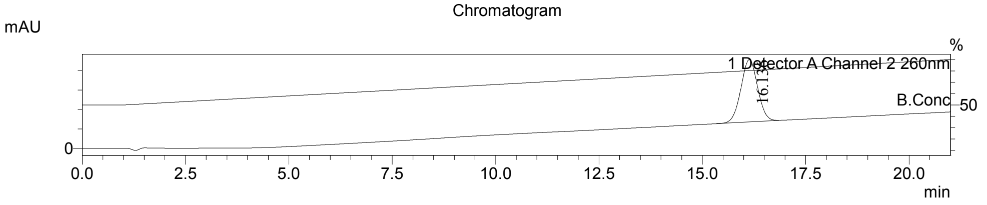

Peak Table

Detector A Channel 1 220nm

Peak#	Ret. Time	Area	Area%
1	16.133	20997790	100.000
Total		20997790	100.000

Peak Table

Detector A Channel 2 260nm

Peak#	Ret. Time	Area	Area%
1	16.136	1805042	100.000
Total		1805042	100.000

2, [D-Trp(Me)]CJ-15,208

Mass spectrum

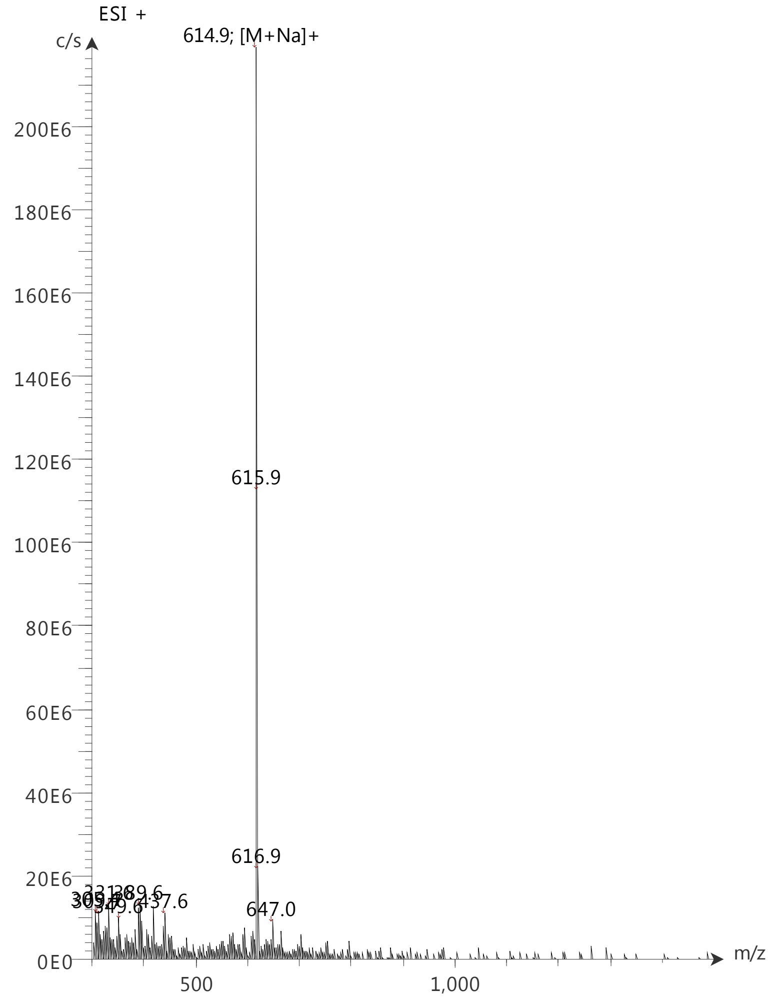

3, [D-Trp(CHO)]CJ-15,208
UHPLC, System 1

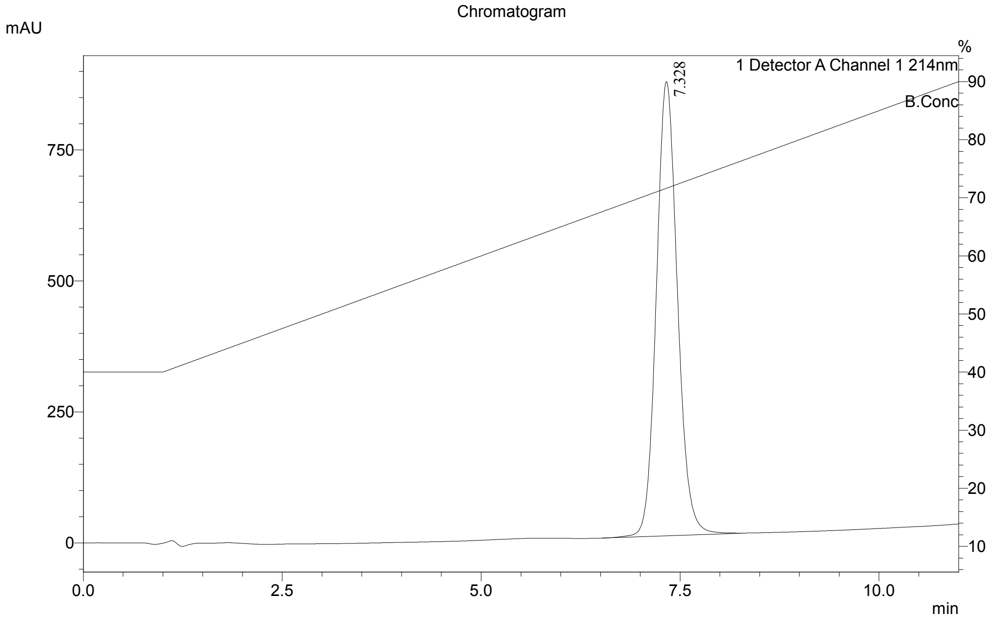

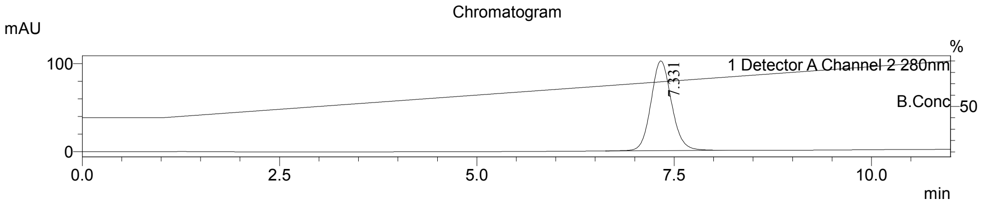

Peak Table
Detector A Channel 1 214nm

Peak#	Ret. Time	Area	Area%
1	7.328	16019228	100.000
Total		16019228	100.000

Peak Table
Detector A Channel 2 280nm

Peak#	Ret. Time	Area	Area%
1	7.331	1856811	100.000
Total		1856811	100.000

3, [D-Trp(CHO)]CJ-15,208
UHPLC, System 2

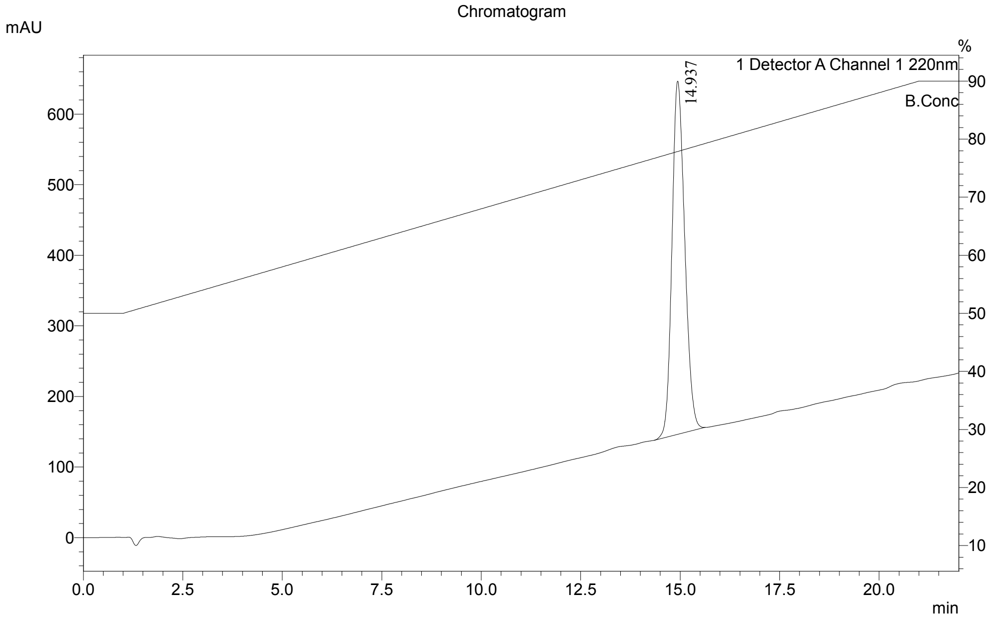

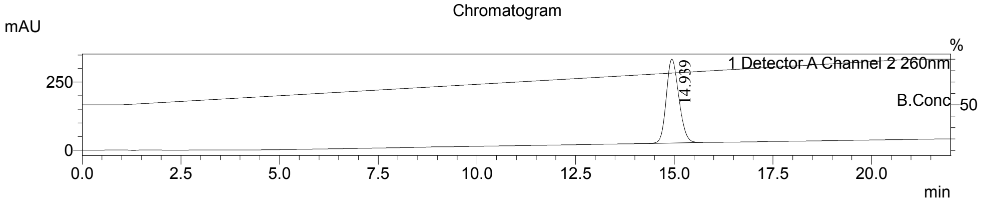

Peak Table
Detector A Channel 1 220nm

Peak#	Ret. Time	Area	Area%
1	14.937	11147829	100.000
Total		11147829	100.000

Peak Table
Detector A Channel 2 260nm

Peak#	Ret. Time	Area	Area%
1	14.939	6823245	100.000
Total		6823245	100.000

3, [D-Trp(CHO)]CJ-15,208

Mass spectrum

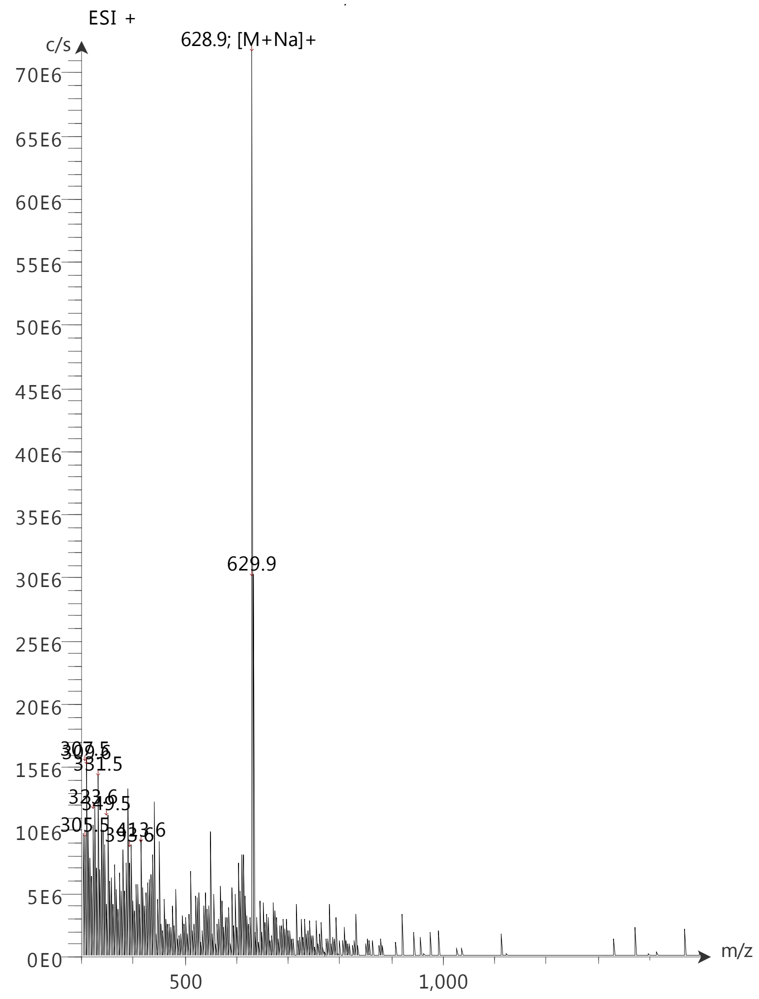

4, [D-Phe[4]]CJ-15,208

HPLC, System 1

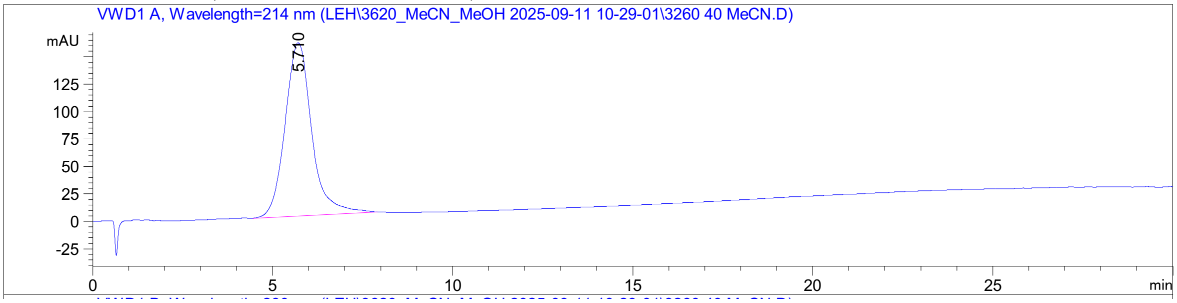

Signal 1: VWD1 A, Wavelength=214 nm

Peak #	RetTime [min]	Type	Width [min]	Area [mAU*s]	Height [mAU]	Area %
1	5.710	BB	0.7864	8121.90186	157.81300	100.0000

Totals : 8121.90186 157.81300

HPLC, System 2

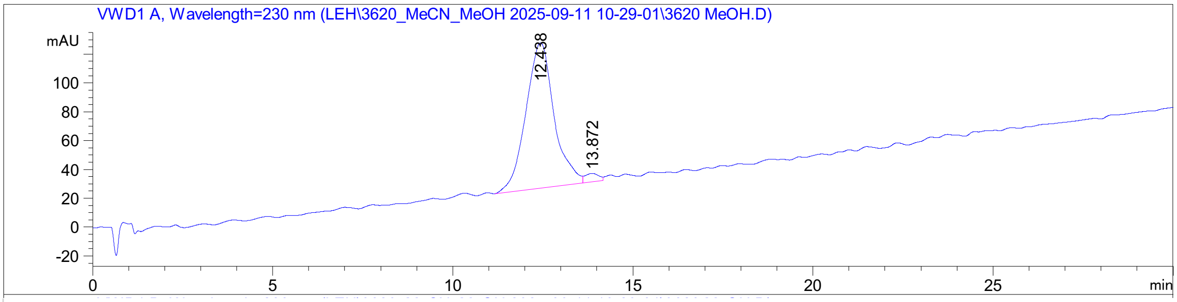

Signal 1: VWD1 A, Wavelength=230 nm

Peak #	RetTime [min]	Type	Width [min]	Area [mAU*s]	Height [mAU]	Area %
1	12.438	BV	0.8240	5492.88232	100.76444	97.1724
2	13.872	VV	0.4038	159.83719	5.90451	2.8276

Totals : 5652.71951 106.66895

4, [D-Phe⁴]CJ-15,208

Mass spectrum

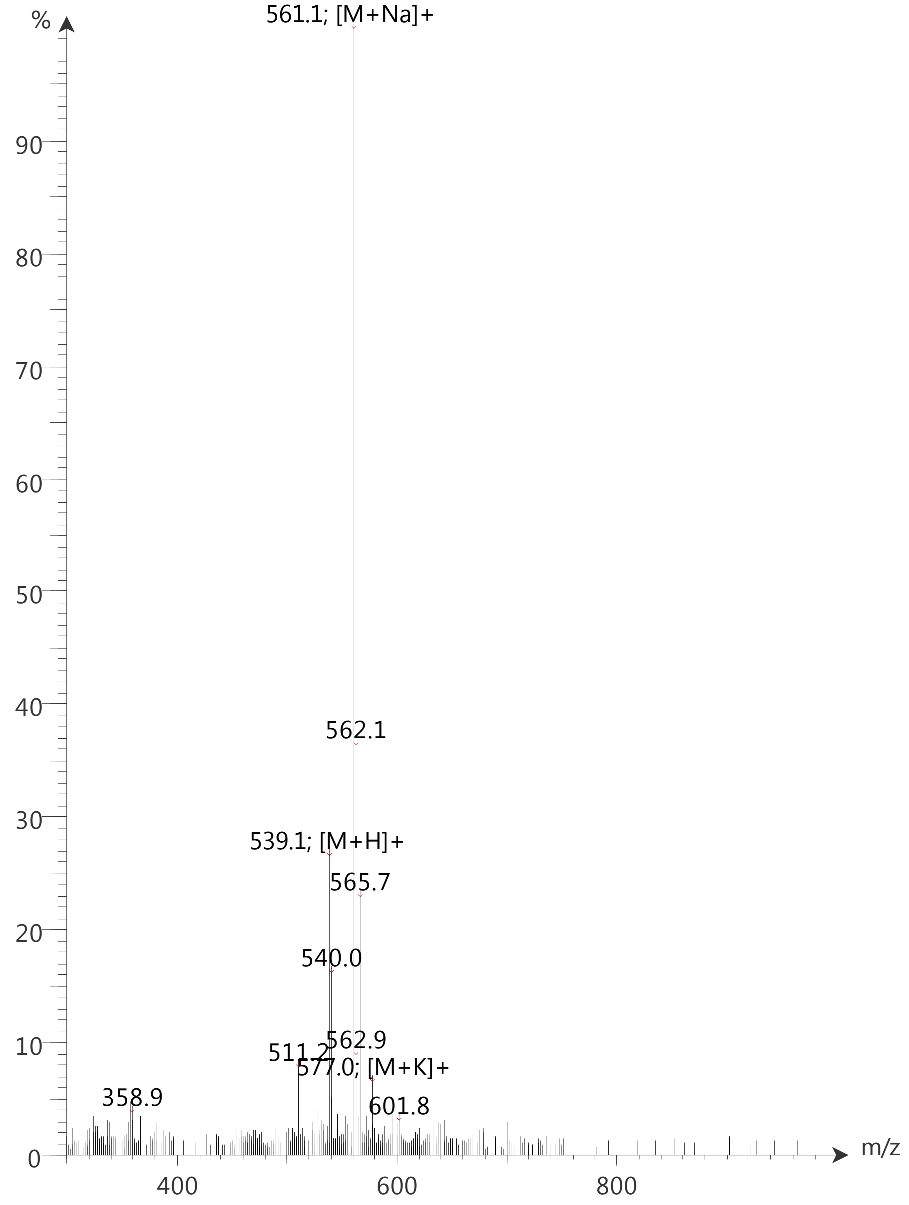

5, [D-Phe(o-F)[4]]CJ-15,208

UHPLC, System 1

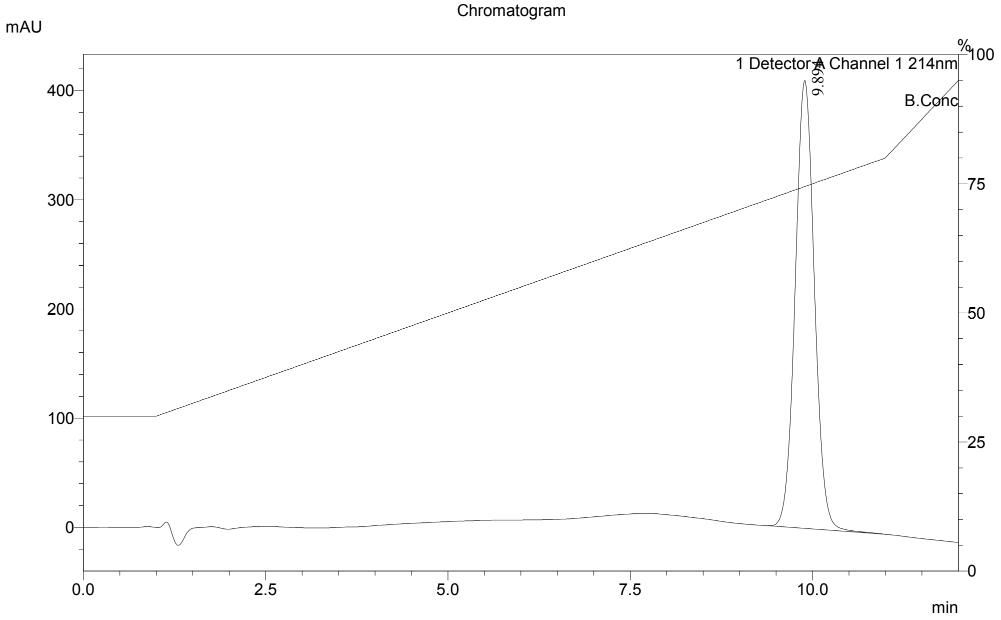

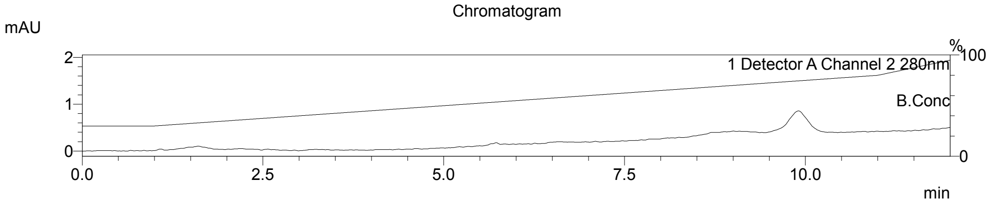

Peak Table

Detector A Channel 1 214nm

Peak#	Ret. Time	Area	Area%
1	9.894	7515306	100.000
Total		7515306	100.000

Peak Table

Detector A Channel 2 280nm

Peak#	Ret. Time	Area	Area%
Total			

5, [D-Phe(o-F)[4]]CJ-15,208

UHPLC, System 2

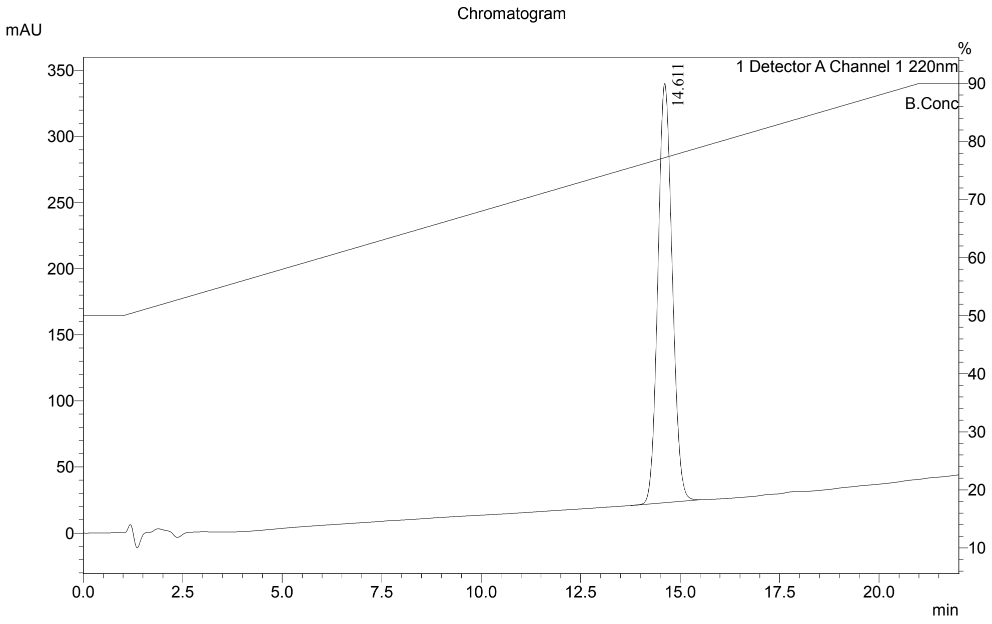

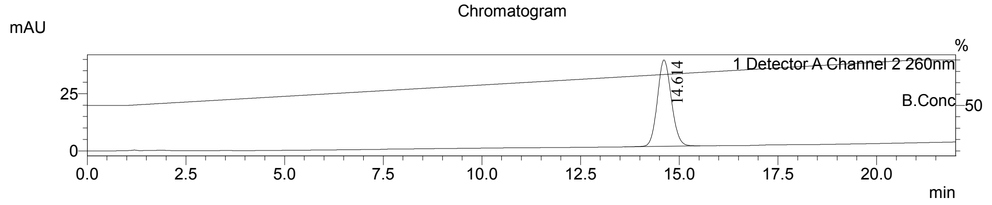

Peak Table

Detector A Channel 1 220nm

Peak#	Ret. Time	Area	Area%
1	14.611	7973377	100.000
Total		7973377	100.000

Peak Table

Detector A Channel 2 260nm

Peak#	Ret. Time	Area	Area%
1	14.614	932814	100.000
Total		932814	100.000

5, [D-Phe(o-F)[4]]CJ-15,208

Mass spectrum

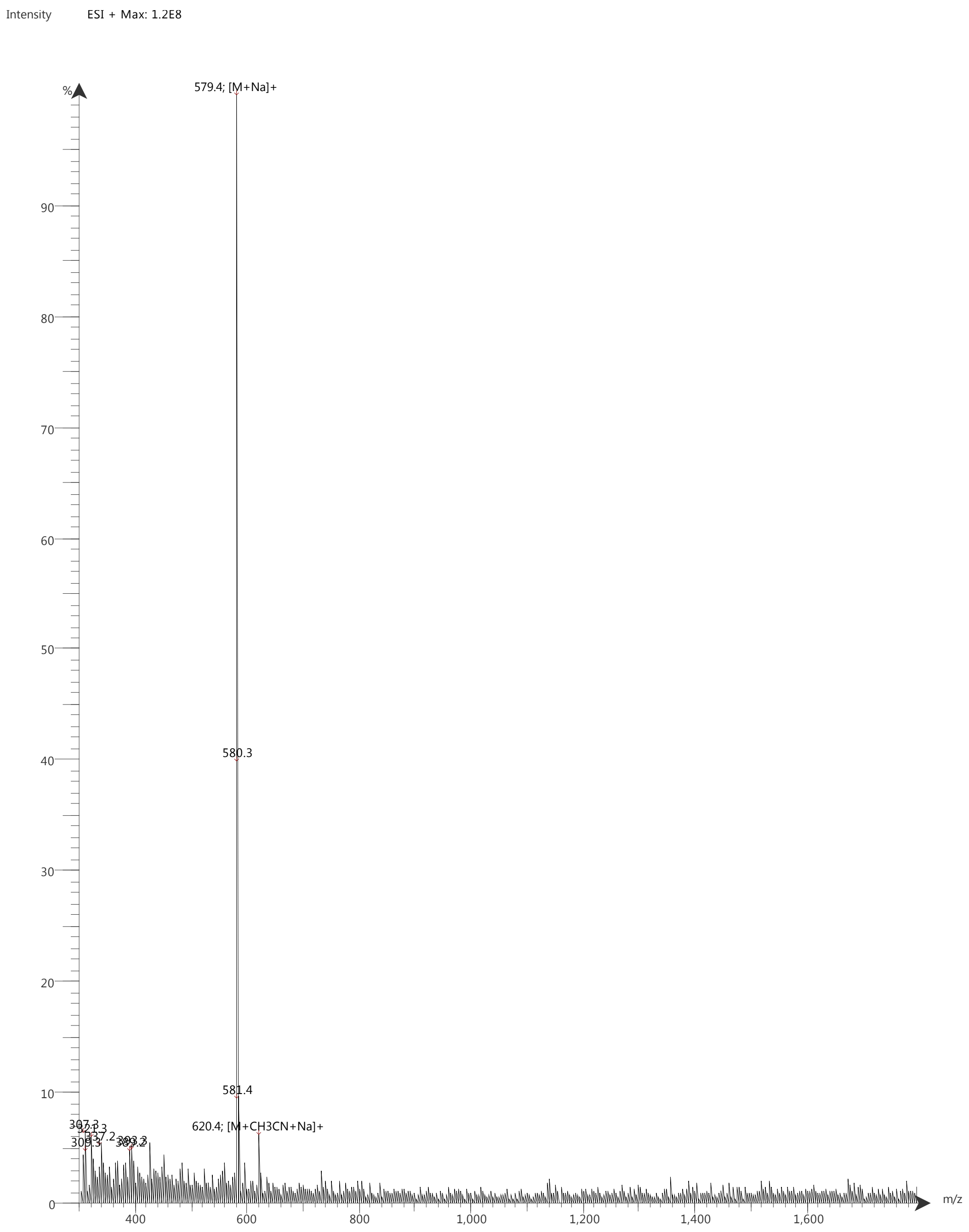

6, [D-Phe(m-F)[4]]CJ-15,208

UHPLC, System 1

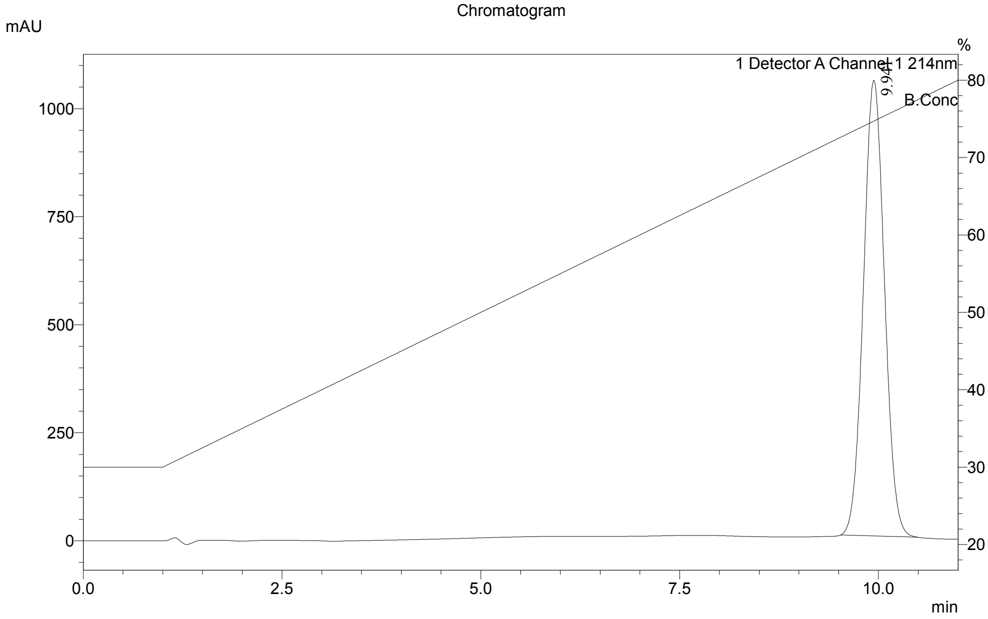

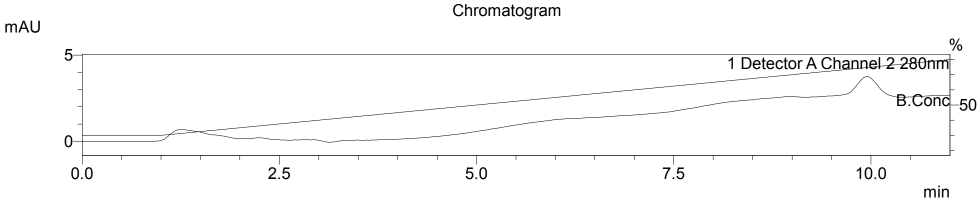

Peak Table

Detector A Channel 1 214nm

Peak#	Ret. Time	Area	Area%
1	9.941	19395664	100.000
Total		19395664	100.000

Peak Table

Detector A Channel 2 280nm

Peak#	Ret. Time	Area	Area%
Total			

6, [D-Phe(m-F)[4]]CJ-15,208

UHPLC, System 2

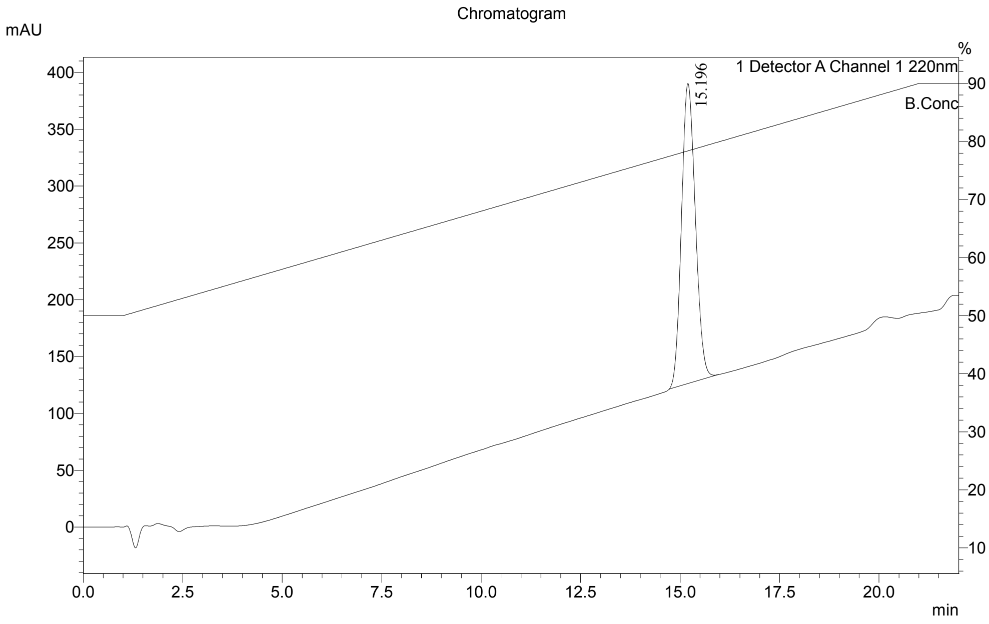

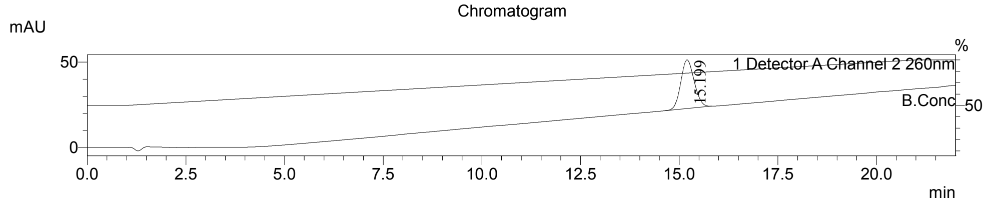

Peak Table

Detector A Channel 1 220nm

Peak#	Ret. Time	Area	Area%
1	15.196	6388227	100.000
Total		6388227	100.000

Peak Table

Detector A Channel 2 260nm

Peak#	Ret. Time	Area	Area%
1	15.199	675490	100.000
Total		675490	100.000

6, [D-Phe(m-F)[4]]CJ-15,208

Mass spectrum

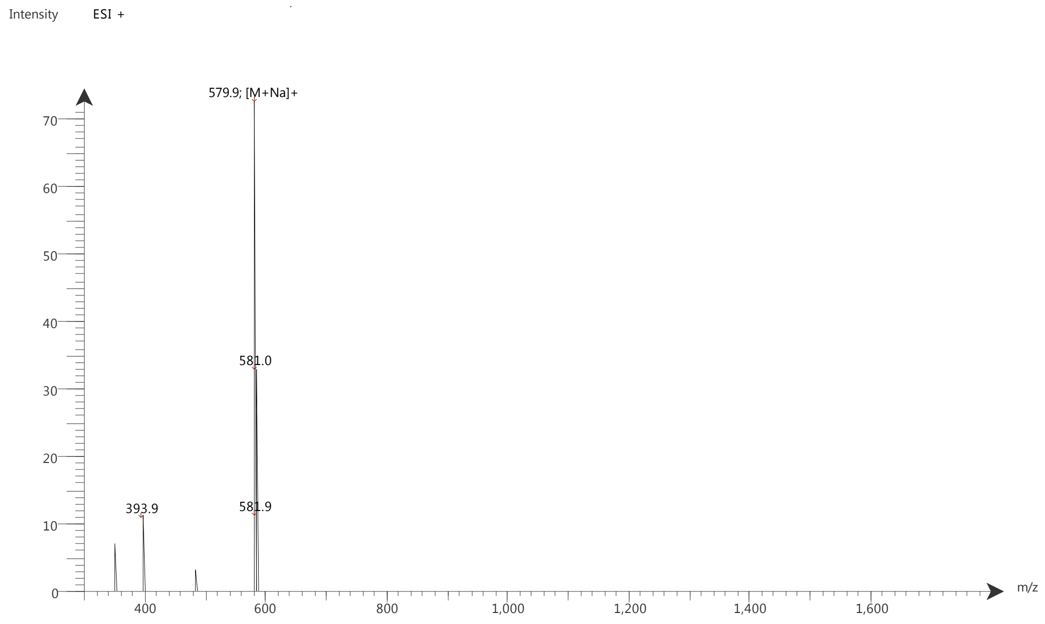

7, [D-Phe(p-F)[4]]CJ-15,208

UHPLC, System 1

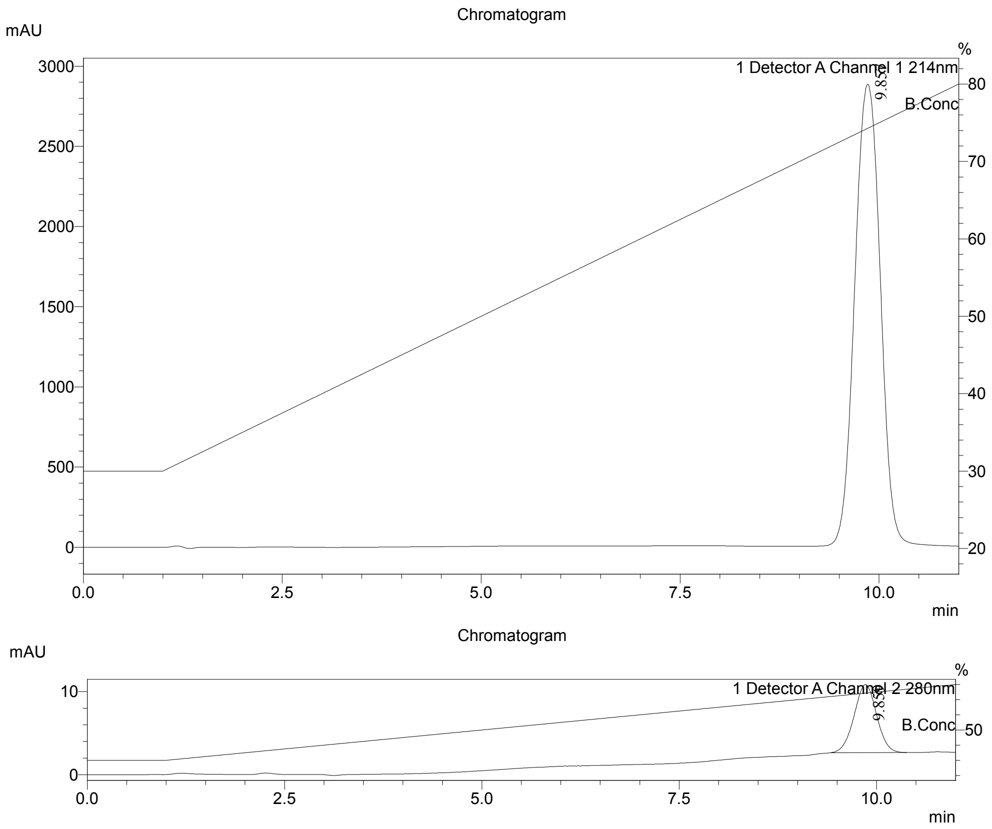

Peak Table
Detector A Channel 1 214nm

Peak#	Ret. Time	Area	Area%
1	9.857	64470530	100.000
Total		64470530	100.000

Peak Table
Detector A Channel 2 280nm

Peak#	Ret. Time	Area	Area%
1	9.859	162811	100.000
Total		162811	100.000

7, [D-Phe(p-F)[4]]CJ-15,208

UHPLC, System 2

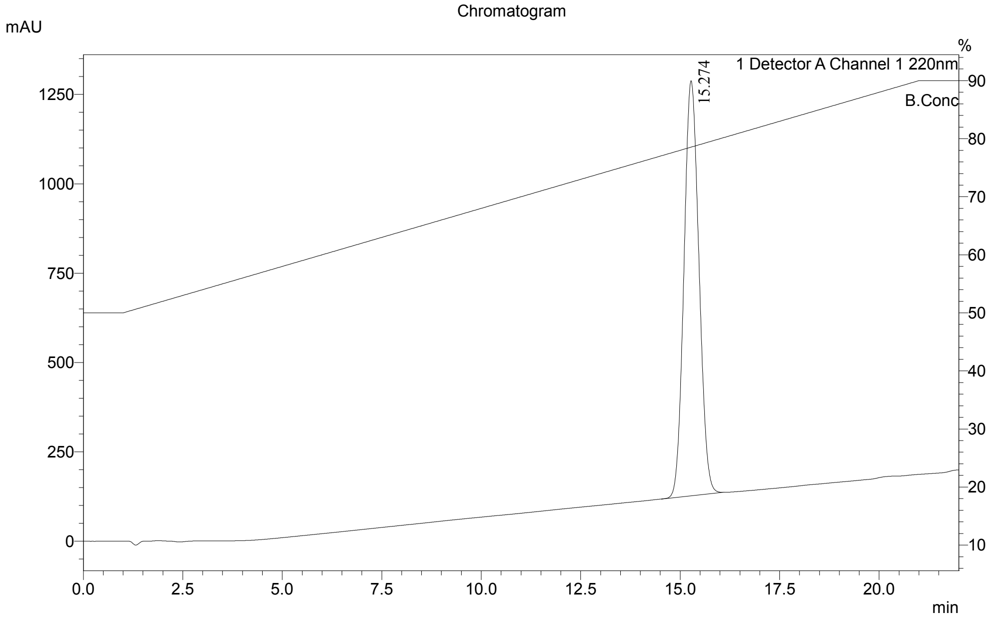

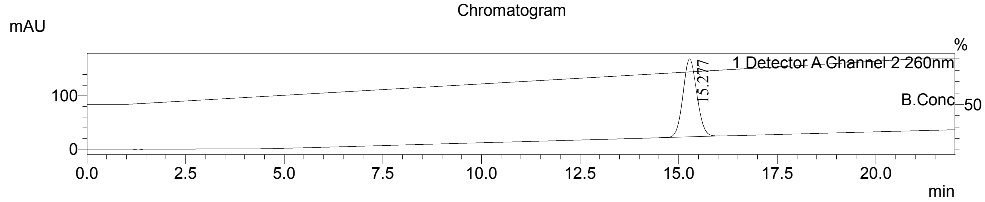

Peak Table
Detector A Channel 1 220nm

Peak#	Ret. Time	Area	Area%
1	15.274	30460837	100.000
Total		30460837	100.000

Peak Table
Detector A Channel 2 260nm

Peak#	Ret. Time	Area	Area%
1	15.277	3584531	100.000
Total		3584531	100.000

7, [D-Phe(p-F)^4]CJ-15,208

Mass spectrum

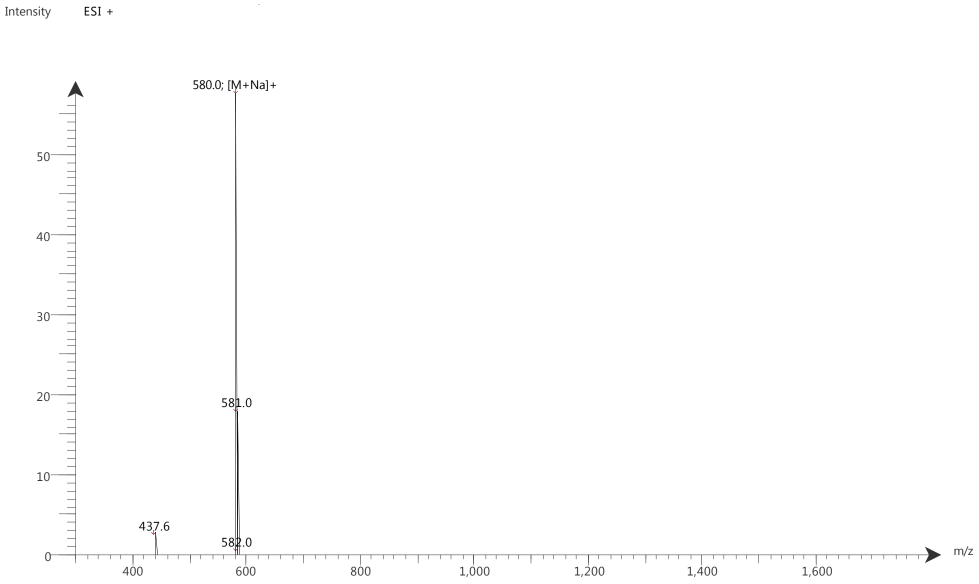

8, [D-Phe(3,4-F_2)[4]]CJ-15,208

UHPLC, System 1

.

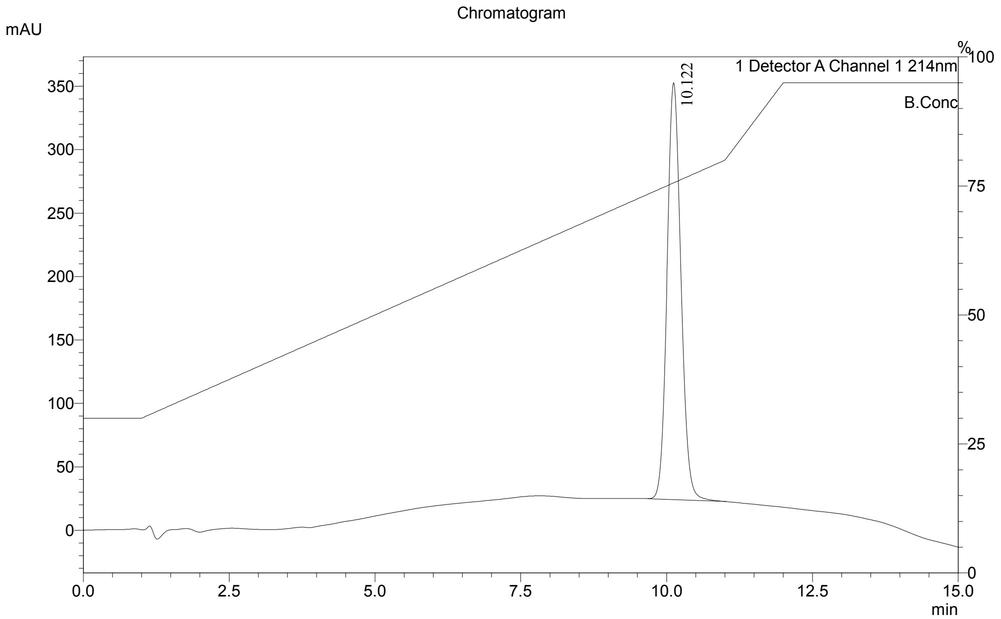

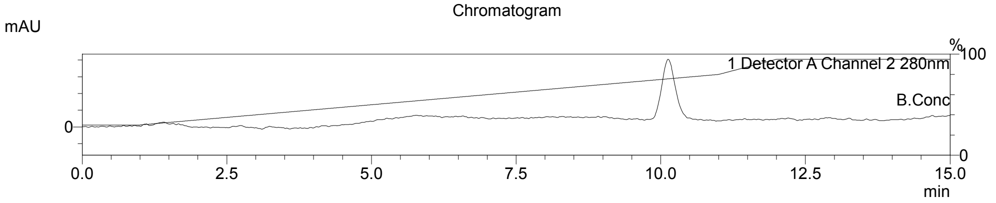

Peak Table

Detector A Channel 1 214nm

Peak#	Ret. Time	Area	Area%
1	10.122	5444548	100.000
Total		5444548	100.000

Peak Table

Detector A Channel 2 280nm

Peak#	Ret. Time	Area	Area%
Total			

8, [D-Phe(3,4-F_2)[4]]CJ-15,208

UHPLC, System 2

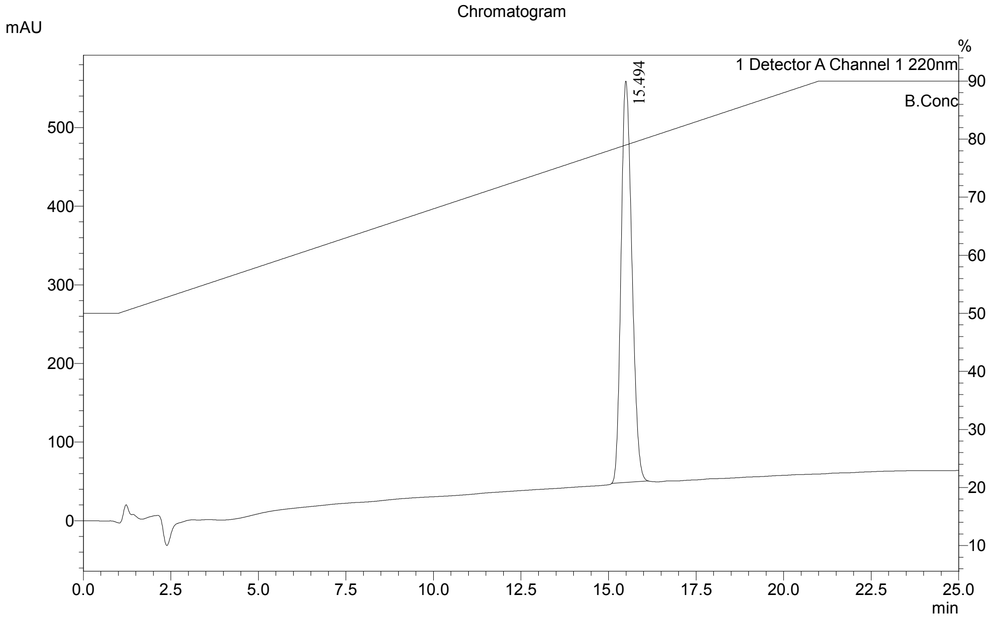

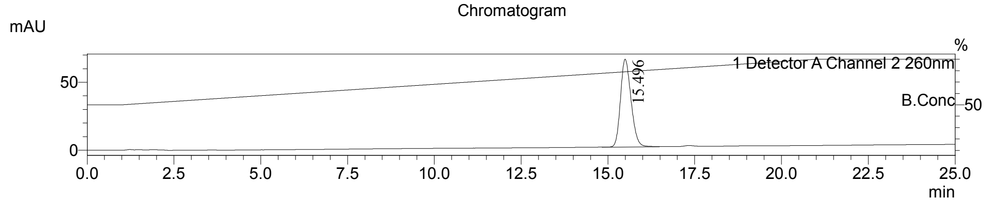

Peak Table

Detector A Channel 1 220nm

Peak#	Ret. Time	Area	Area%
1	15.494	11002162	100.000
Total		11002162	100.000

Peak Table

Detector A Channel 2 260nm

Peak#	Ret. Time	Area	Area%
1	15.496	1372034	100.000
Total		1372034	100.000

8, [D-Phe(3,4-F_2)[4]]CJ-15,208

Mass spectrum

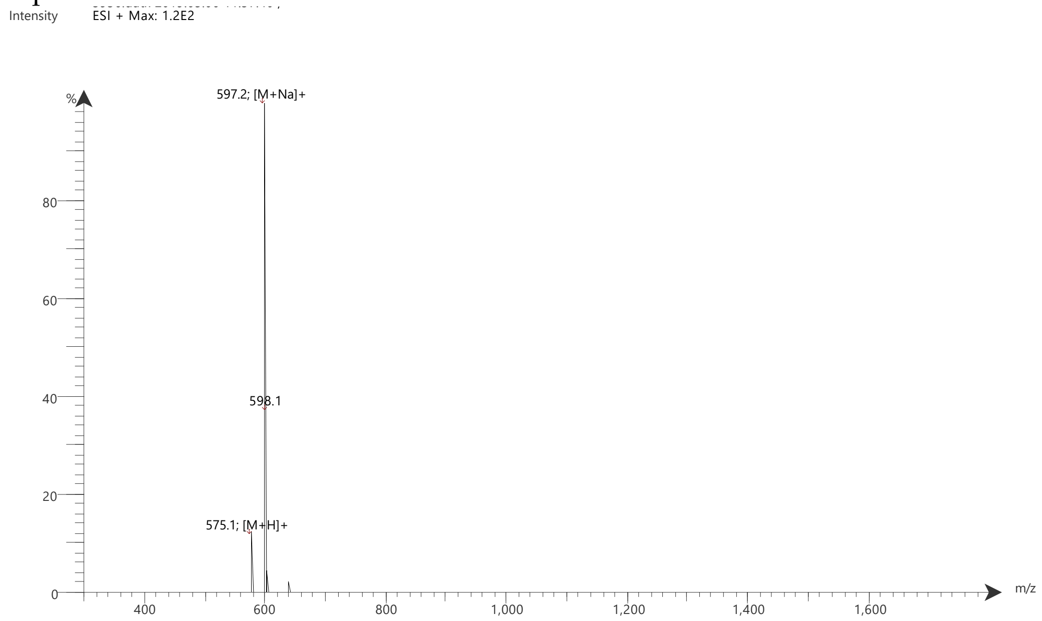

9, [D-Phe(3,5-F_2)^4]CJ-15,208

UHPLC, System 1

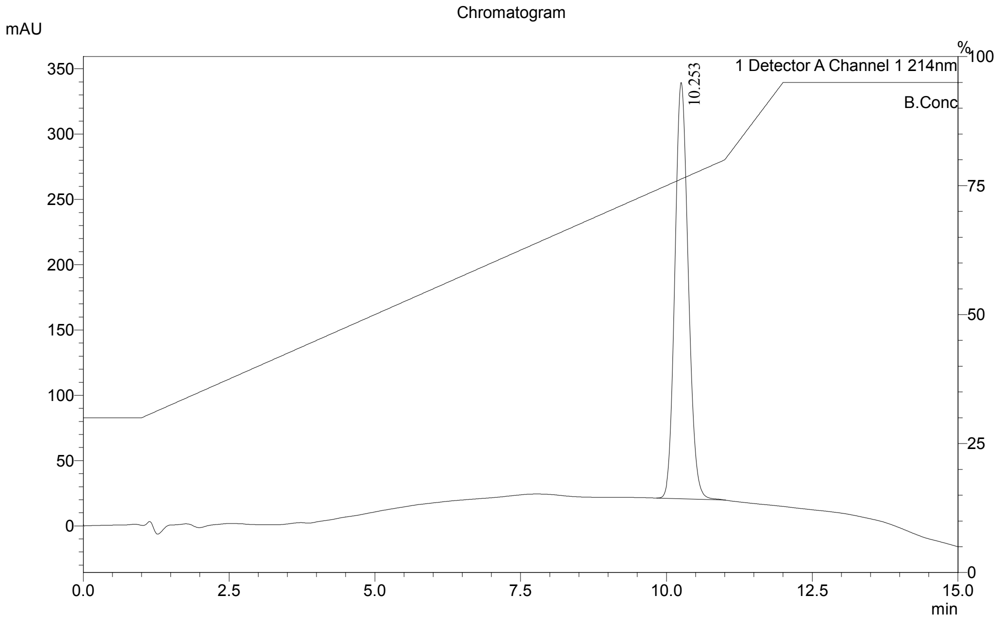

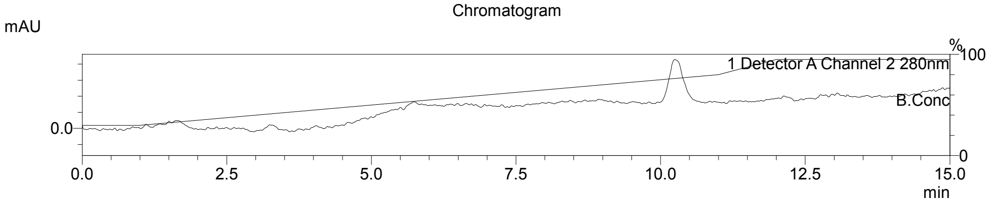

Peak Table

Detector A Channel 1 214nm

Peak#	Ret. Time	Area	Area%
1	10.253	5084271	100.000
Total		5084271	100.000

Peak Table

Detector A Channel 2 280nm

Peak#	Ret. Time	Area	Area%
Total			

9, [D-Phe(3,5-F_2)[4]]CJ-15,208

UHPLC, System 2

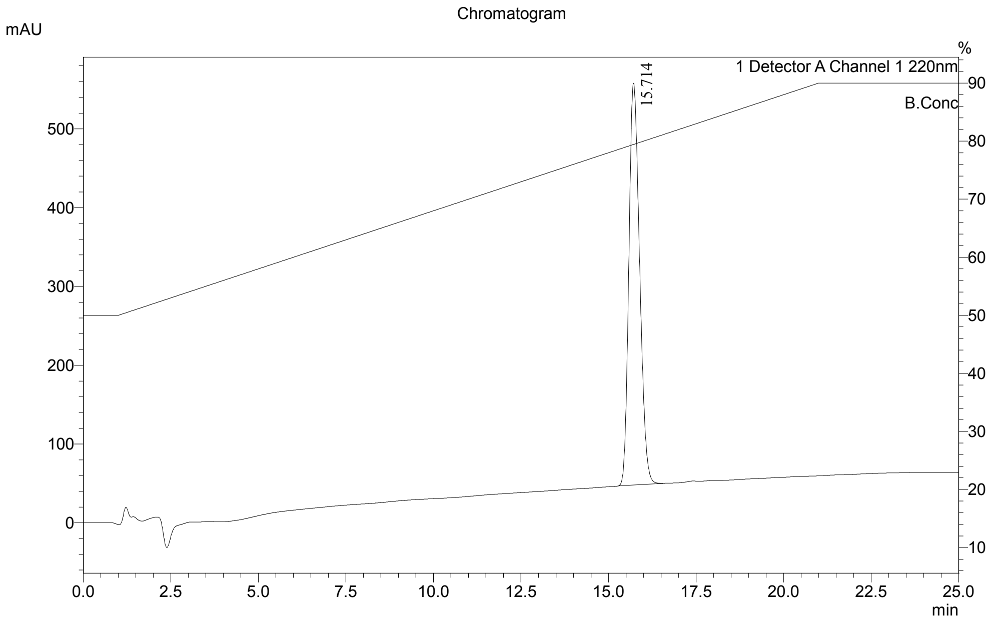

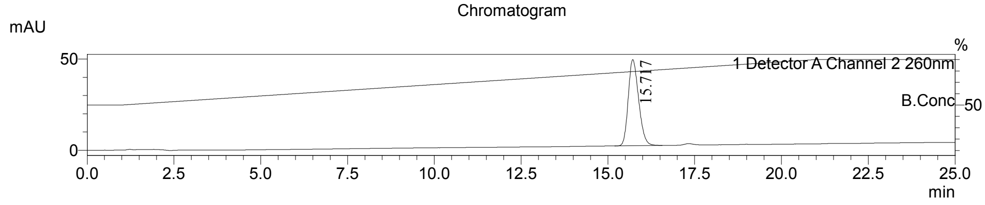

Peak Table

Detector A Channel 1 220nm

Peak#	Ret. Time	Area	Area%
1	15.714	10896879	100.000
Total		10896879	100.000

Peak Table

Detector A Channel 2 260nm

Peak#	Ret. Time	Area	Area%
1	15.717	982929	100.000
Total		982929	100.000

9, [D-Phe(3,5-F_2)^4]CJ-15,208

Mass spectrum

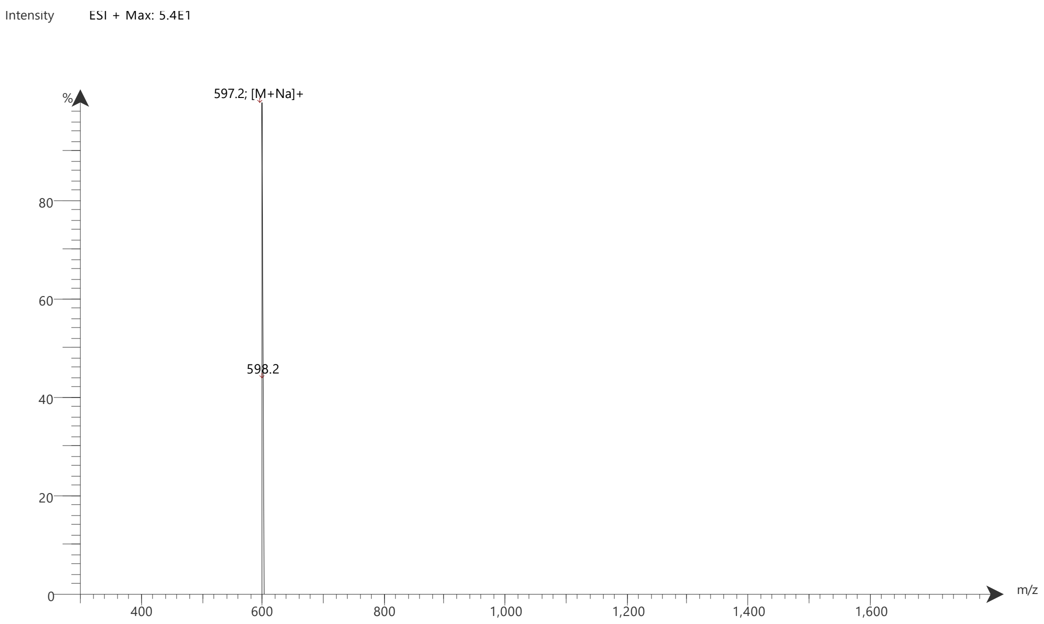

10, [D-Phe(2,4,5-F_3)^4]CJ-15,208

UHPLC, System 1

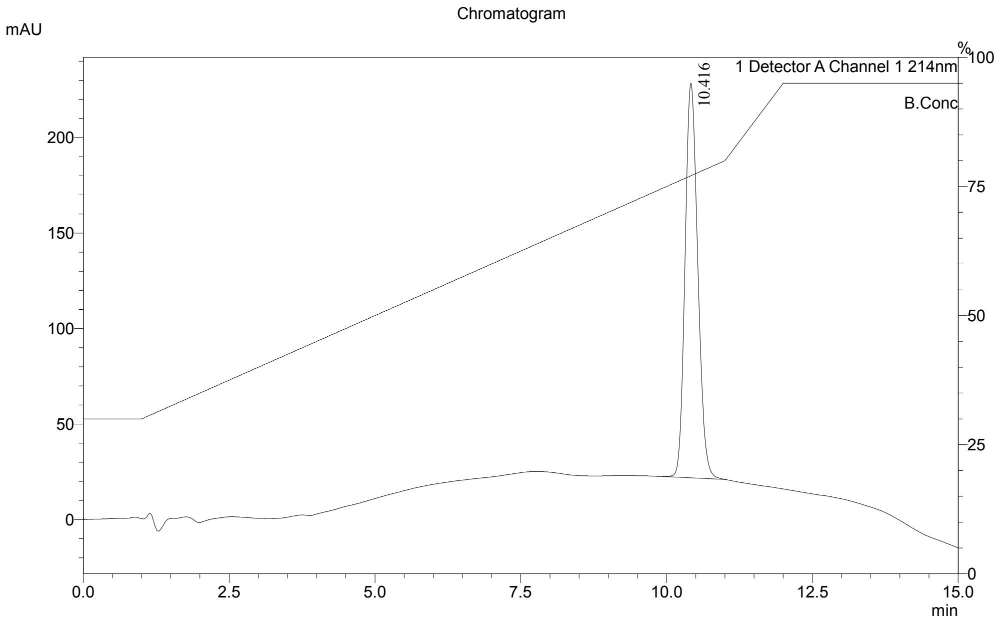

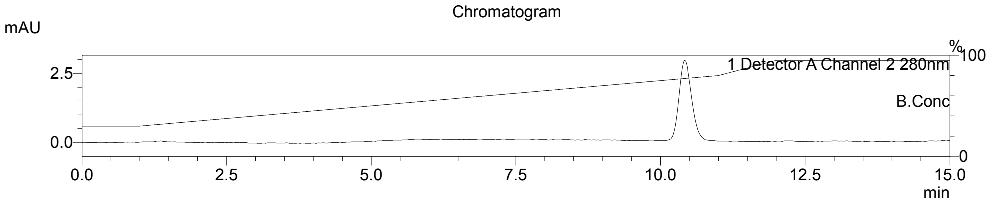

Peak Table

Detector A Channel 1 214nm

Peak#	Ret. Time	Area	Area%
1	10.416	3130238	100.000
Total		3130238	100.000

Peak Table

Detector A Channel 2 280nm

Peak#	Ret. Time	Area	Area%
Total			

10, [D-Phe(2,4,5-F_3)^4]CJ-15,208

UHPLC, System 2

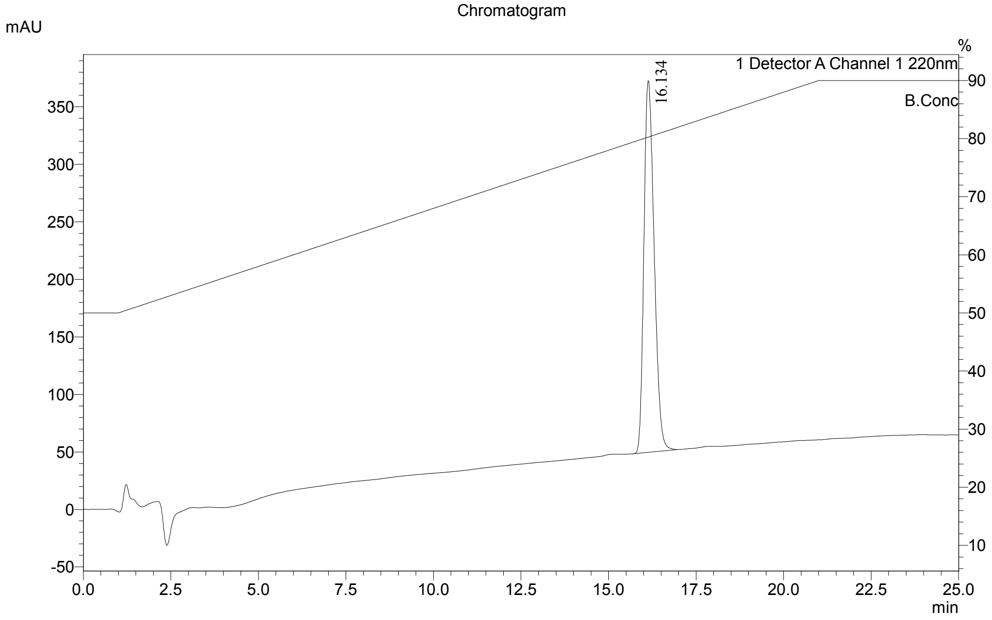

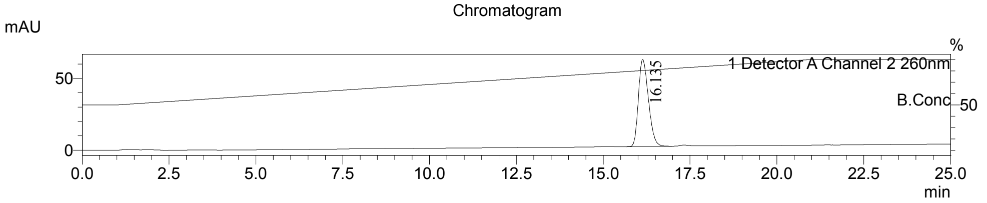

Peak Table

Detector A Channel 1 220nm

Peak#	Ret. Time	Area	Area%
1	16.134	6585584	100.000
Total		6585584	100.000

Peak Table

Detector A Channel 2 260nm

Peak#	Ret. Time	Area	Area%
1	16.135	1214672	100.000
Total		1214672	100.000

10, [D-Phe(2,4,5-F_3)^4]CJ-15,208

Mass spectrum

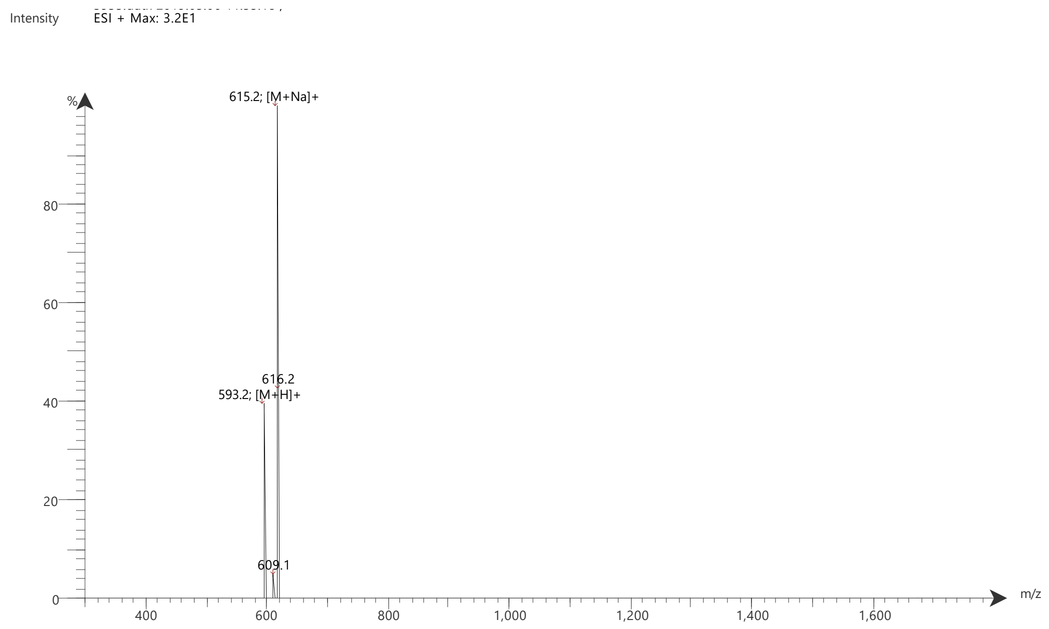

11, [D-Phe(3,4,5-F_3)[4]]CJ-15,208
UHPLC, System 1

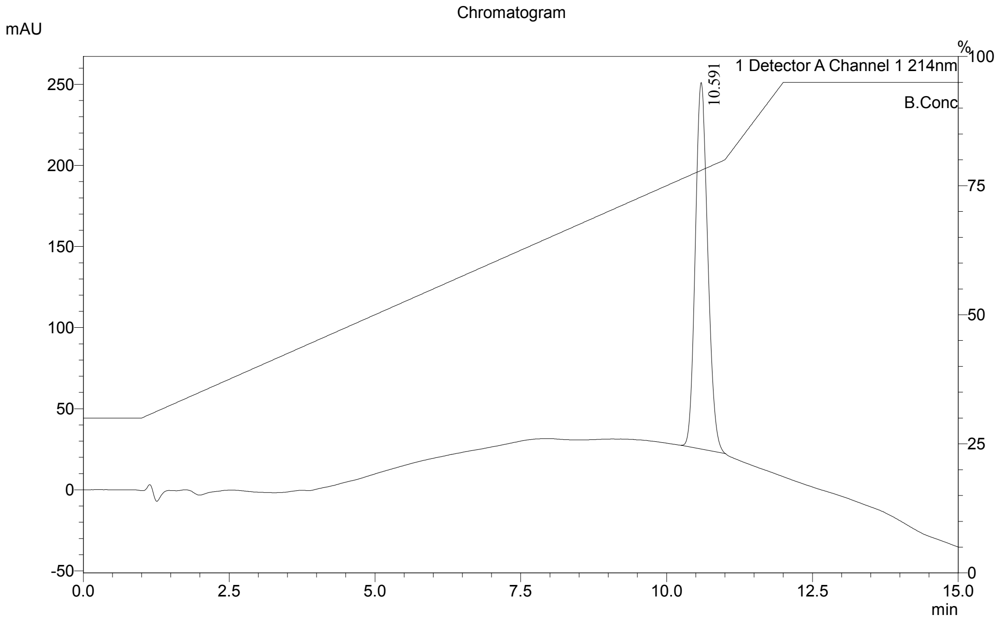

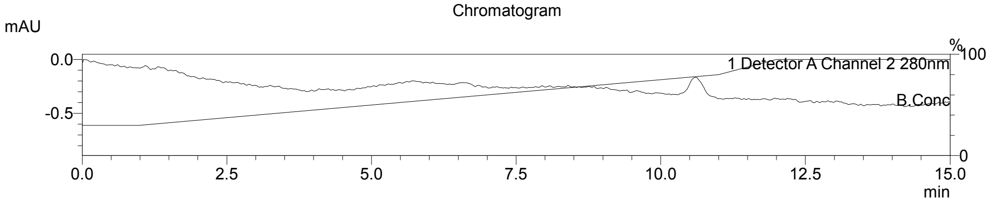

Peak Table
Detector A Channel 1 214nm

Peak#	Ret. Time	Area	Area%
1	10.591	3385552	100.000
Total		3385552	100.000

Peak Table
Detector A Channel 2 280nm

Peak#	Ret. Time	Area	Area%
Total			

11, [D-Phe(3,4,5-F_3)[4]]CJ-15,208
UHPLC, System 2

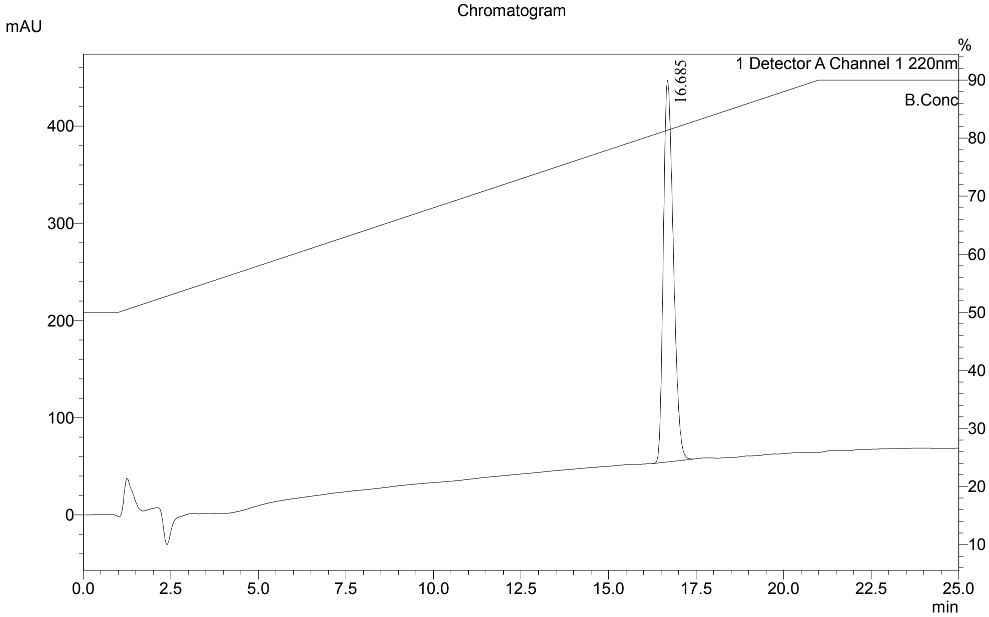

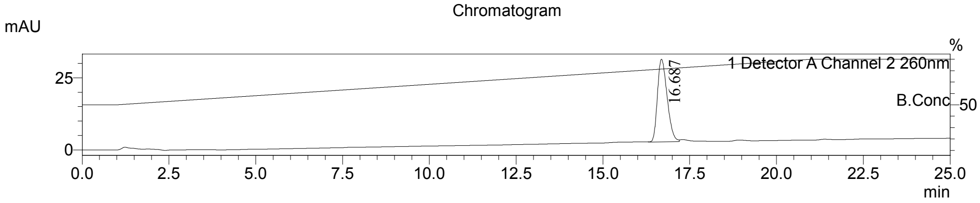

Peak Table
Detector A Channel 1 220nm

Peak#	Ret. Time	Area	Area%
1	16.685	7792065	100.000
Total		7792065	100.000

Peak Table
Detector A Channel 2 260nm

Peak#	Ret. Time	Area	Area%
1	16.687	555878	100.000
Total		555878	100.000

10, [D-Phe(3,4,5-F_3)[4]]CJ-15,208

Mass spectrum

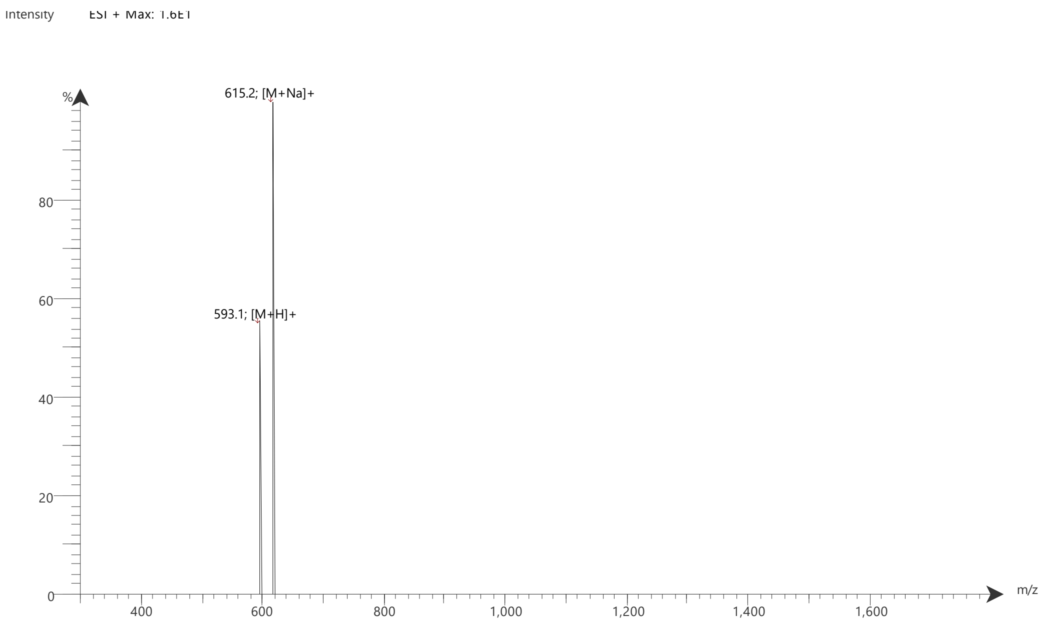

12, [D-Phe(F_5)^4]CJ-15,208
UHPLC, System 1

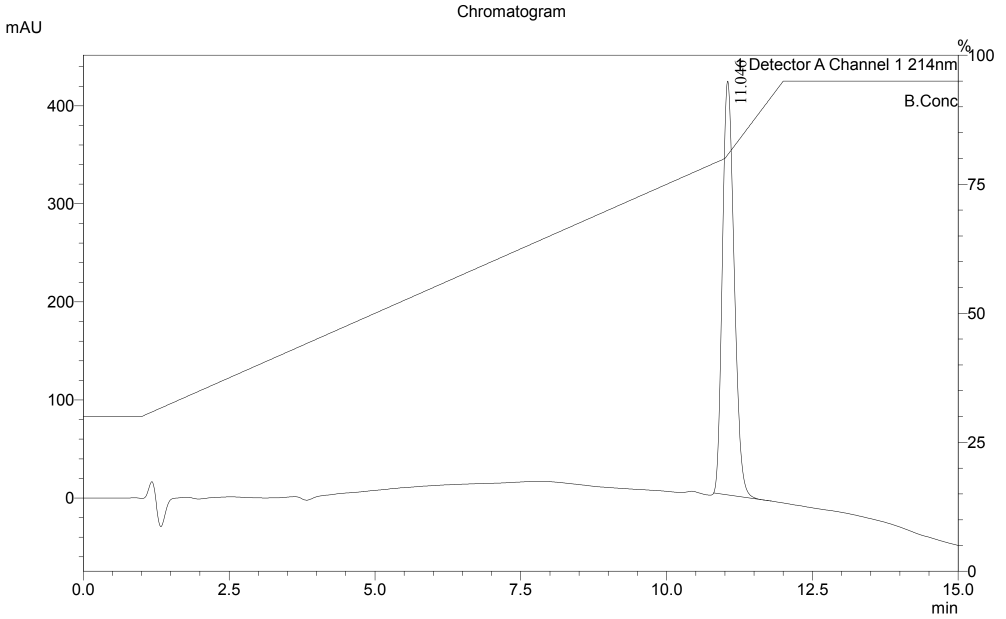

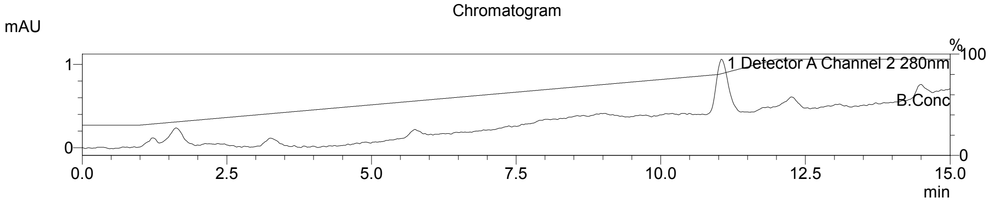

Peak Table
Detector A Channel 1 214nm

Peak#	Ret. Time	Area	Area%
1	11.046	5928534	100.000
Total		5928534	100.000

Peak Table
Detector A Channel 2 280nm

Peak#	Ret. Time	Area	Area%
Total			

12, [D-Phe(F_5)[4]]CJ-15,208

UHPLC, System 2

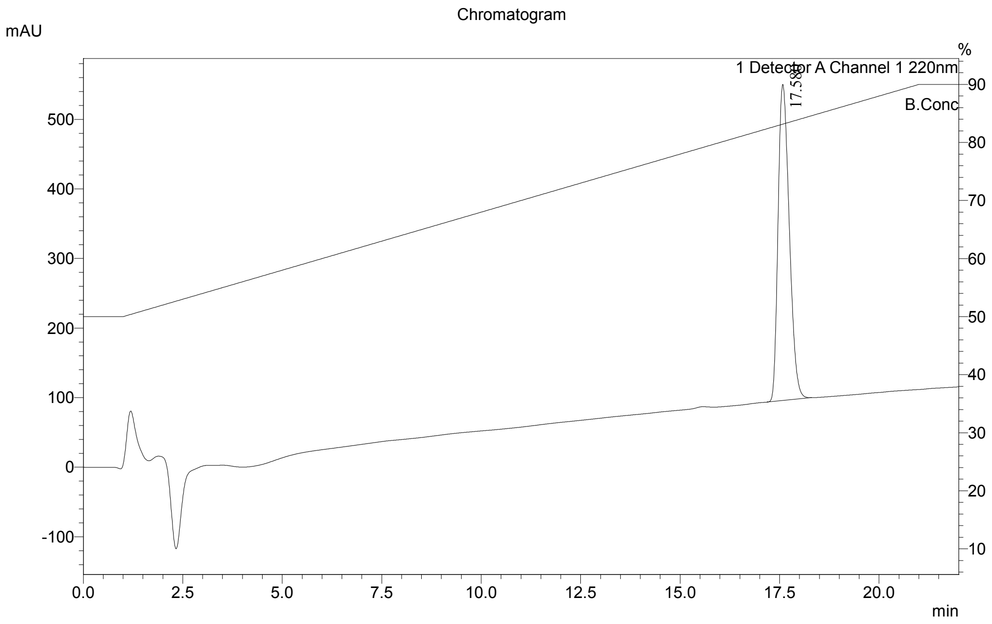

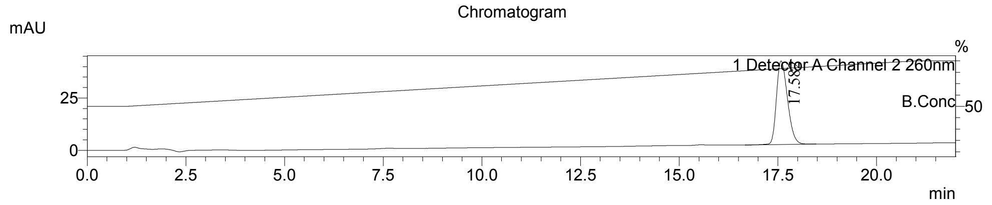

Peak Table

Detector A Channel 1 220nm

Peak#	Ret. Time	Area	Area%
1	17.580	8919662	100.000
Total		8919662	100.000

Peak Table

Detector A Channel 2 260nm

Peak#	Ret. Time	Area	Area%
1	17.582	781904	100.000
Total		781904	100.000

12, [D-Phe(F$_5$)^4]CJ-15,208

Mass spectrum

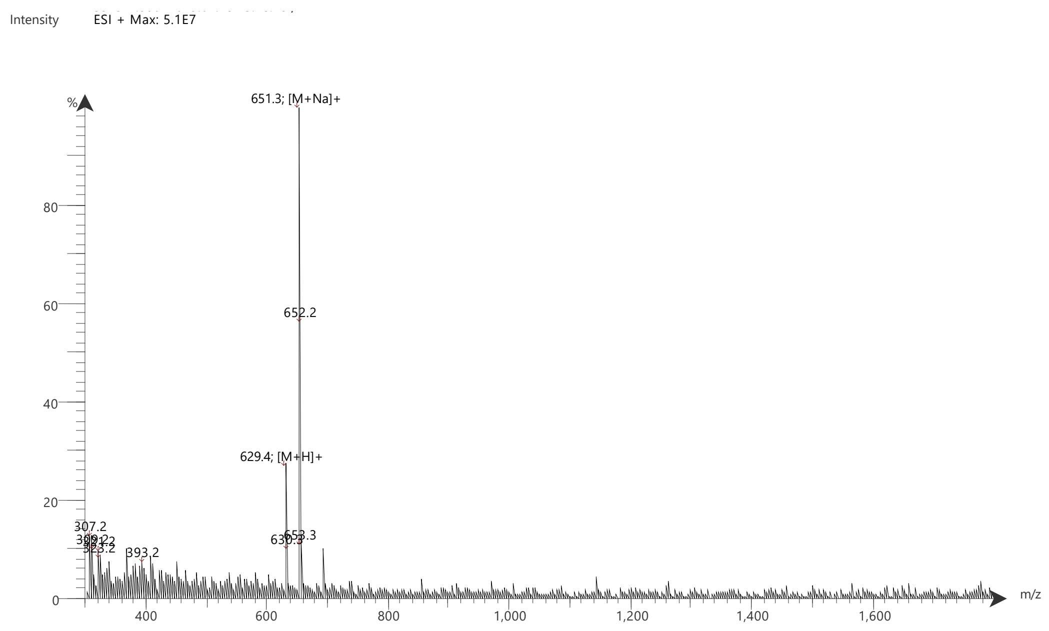

13, [D-Phe(o-Me)[4]]CJ-15,208

UHPLC, System 1

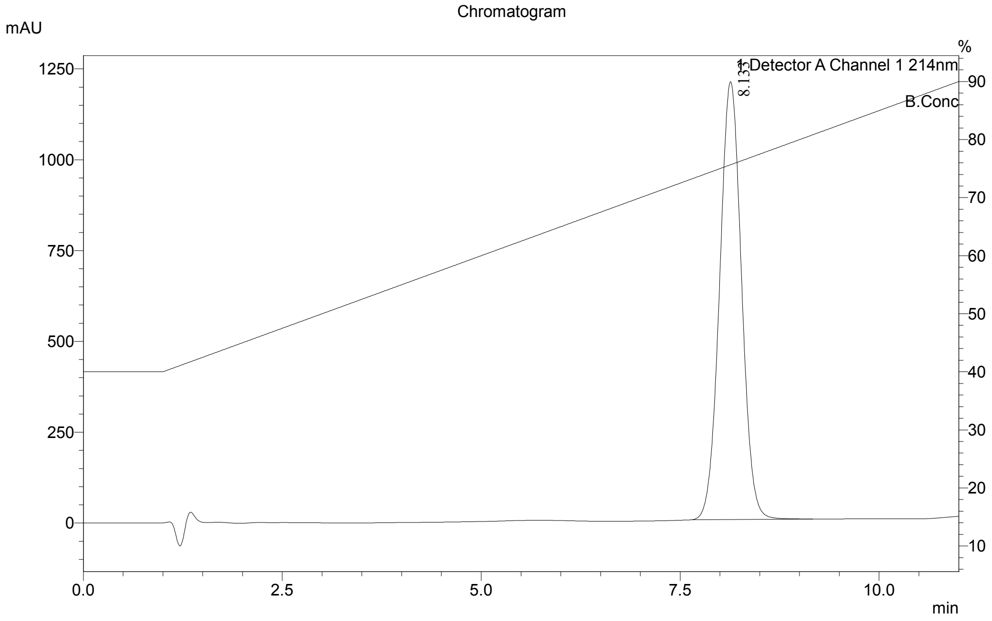

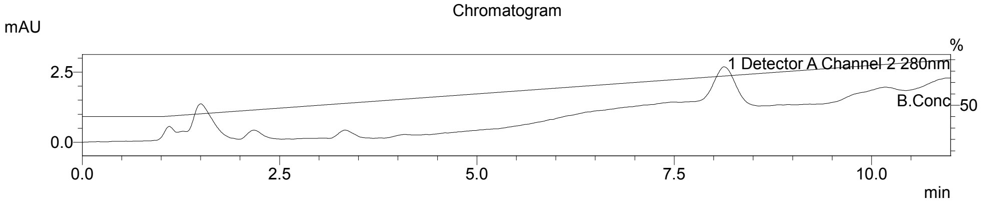

Peak Table

Detector A Channel 1 214nm

Peak#	Ret. Time	Area	Area%
1	8.133	23036123	100.000
Total		23036123	100.000

Peak Table

Detector A Channel 2 280nm

Peak#	Ret. Time	Area	Area%
Total			

13, [D-Phe(o-Me)[4]]CJ-15,208

UHPLC, System 2

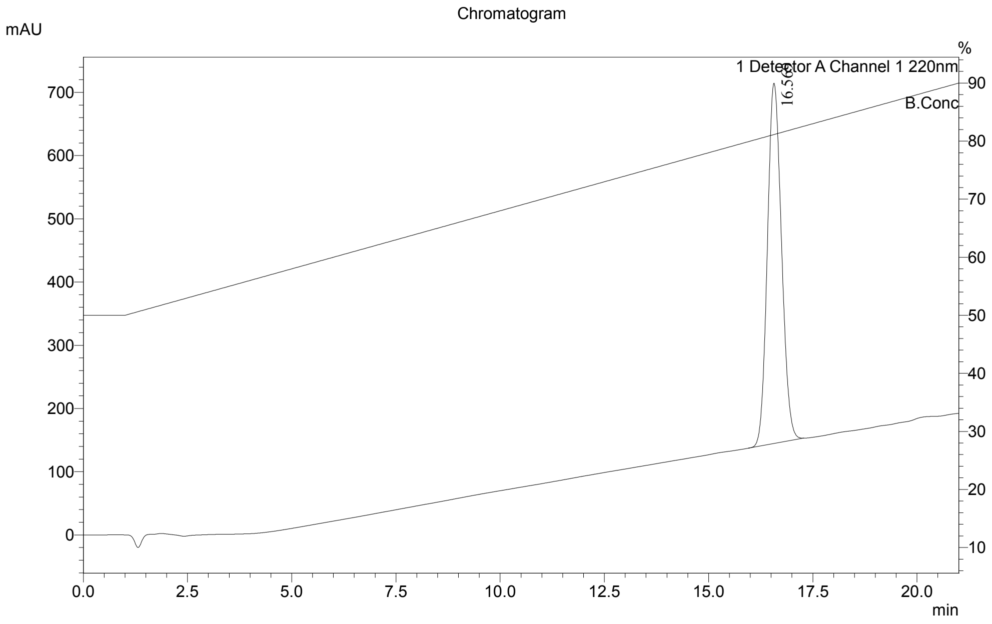

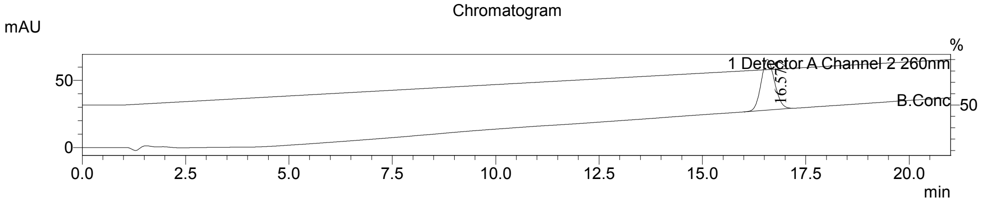

Peak Table

Detector A Channel 1 220nm

Peak#	Ret. Time	Area	Area%
1	16.569	13435507	100.000
Total		13435507	100.000

Peak Table

Detector A Channel 2 260nm

Peak#	Ret. Time	Area	Area%
1	16.572	854658	100.000
Total		854658	100.000

13, [D-Phe(o-Me)[4]]CJ-15,208

Mass spectrum

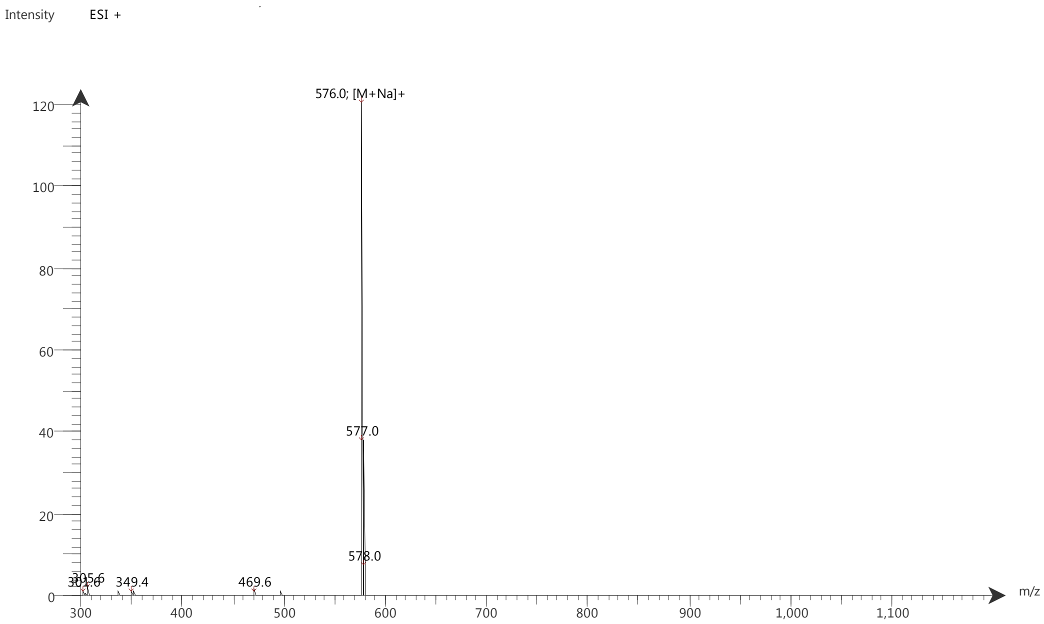

14, [D-Phe(m-Me)[4]]CJ-15,208

UHPLC, System 1

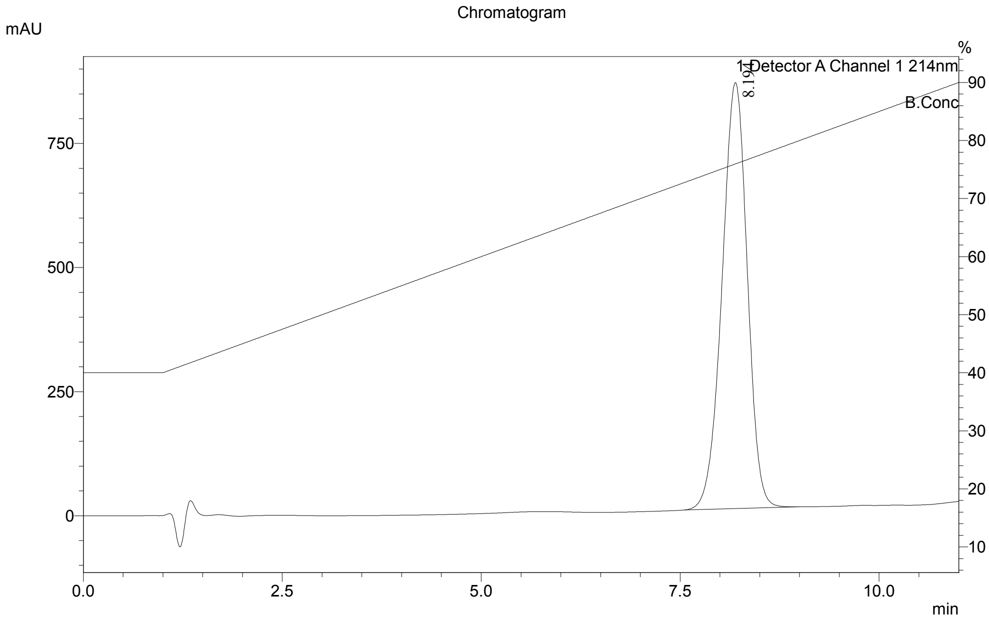

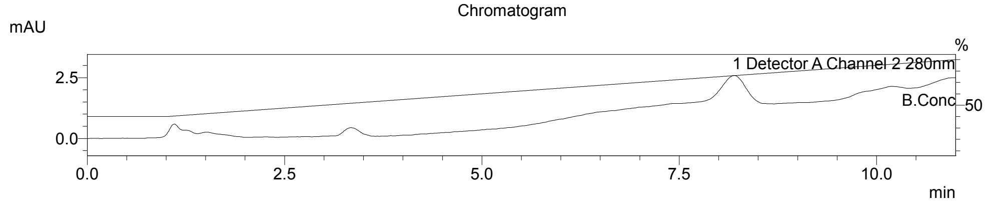

Peak Table

Detector A Channel 1 214nm

Peak#	Ret. Time	Area	Area%
1	8.194	18916843	100.000
Total		18916843	100.000

Peak Table

Detector A Channel 2 280nm

Peak#	Ret. Time	Area	Area%
Total			

14, [D-Phe(m-Me)[4]]CJ-15,208
UHPLC, System 2

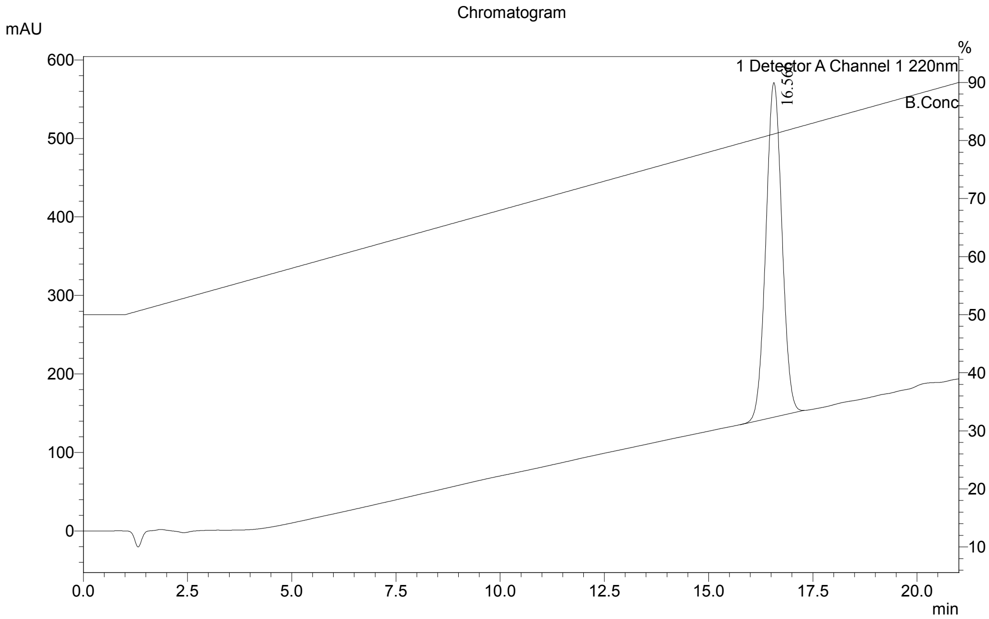

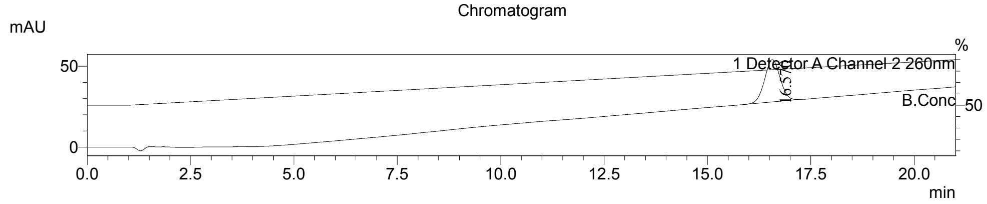

Peak Table
Detector A Channel 1 220nm

Peak#	Ret. Time	Area	Area%
1	16.566	11429855	100.000
Total		11429855	100.000

Peak Table
Detector A Channel 2 260nm

Peak#	Ret. Time	Area	Area%
1	16.570	681944	100.000
Total		681944	100.000

14, [D-Phe(m-Me)[4]]CJ-15,208

Mass spectrum

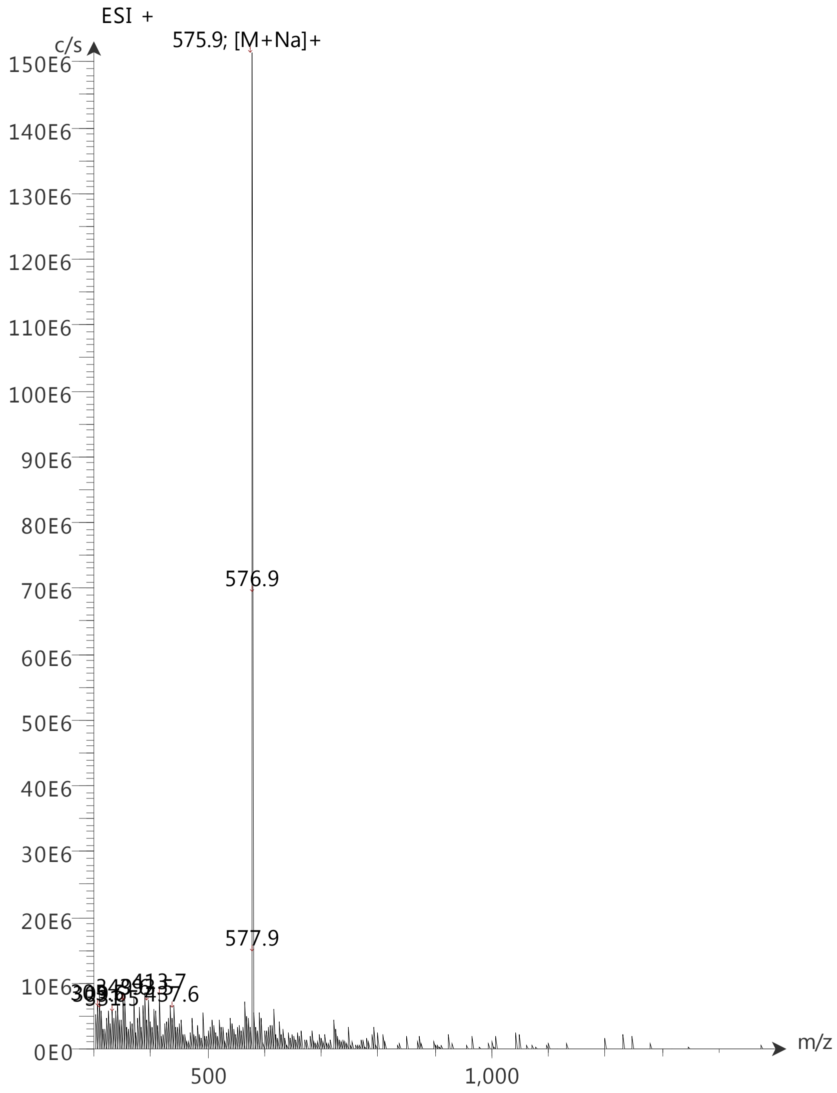

15, [D-Phe(p-Me)[4]]CJ-15,208

UHPLC, System 1

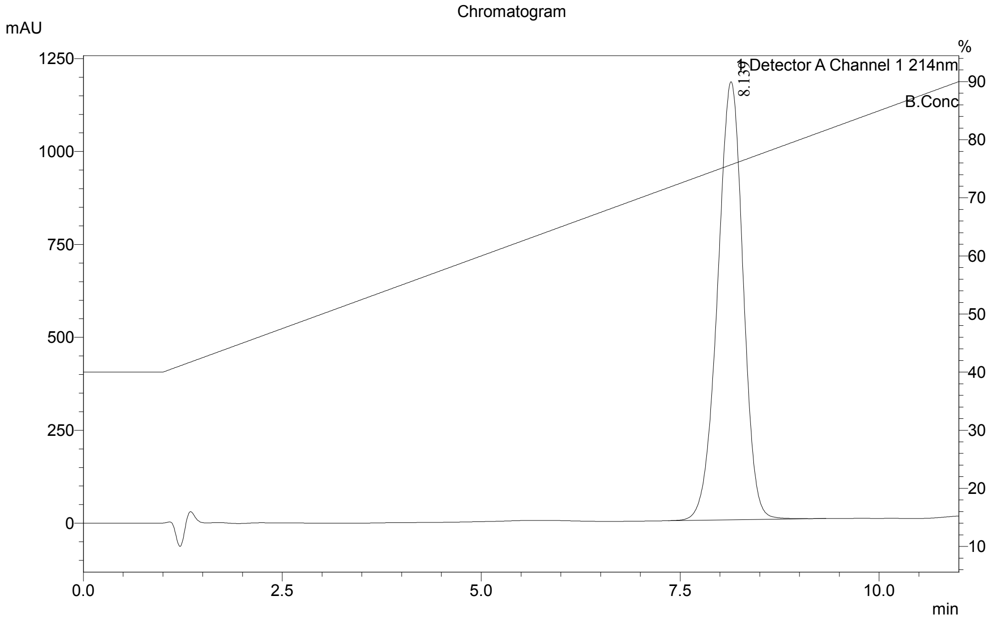

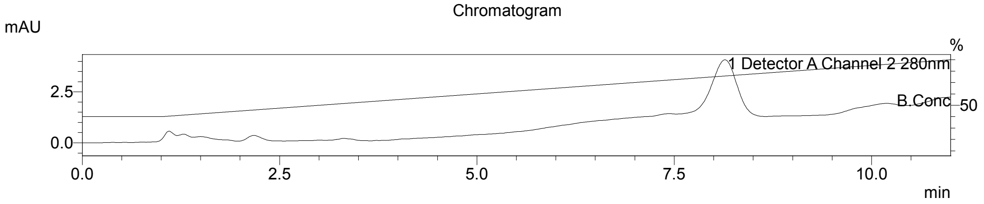

Peak Table

Detector A Channel 1 214nm

Peak#	Ret. Time	Area	Area%
1	8.139	26258734	100.000
Total		26258734	100.000

Peak Table

Detector A Channel 2 280nm

Peak#	Ret. Time	Area	Area%
Total			

15, [D-Phe(p-Me)[4]]CJ-15,208

UHPLC, System 2

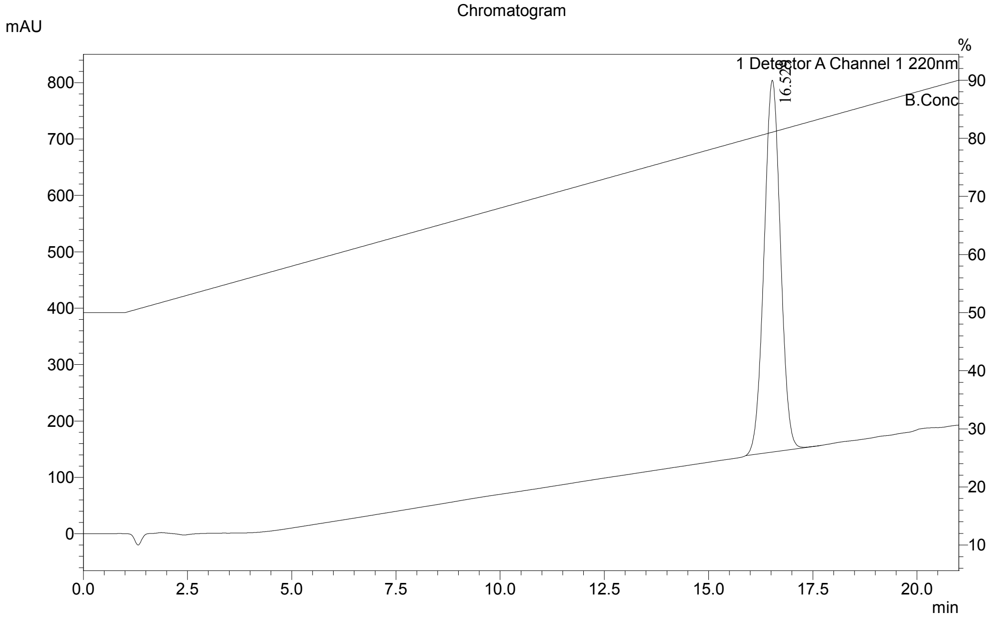

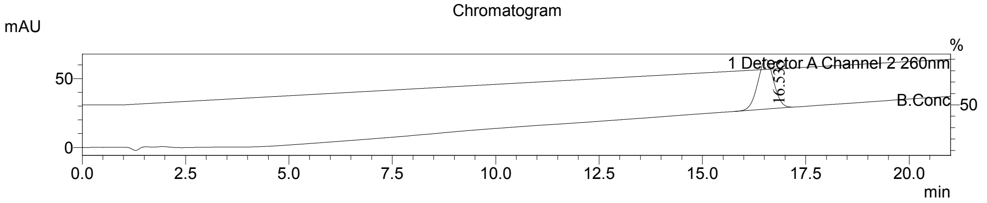

Peak Table

Detector A Channel 1 220nm

Peak#	Ret. Time	Area	Area%
1	16.529	18013462	100.000
Total		18013462	100.000

Peak Table

Detector A Channel 2 260nm

Peak#	Ret. Time	Area	Area%
1	16.533	967345	100.000
Total		967345	100.000

15, [D-Phe(p-Me)[4]]CJ-15,208

Mass spectrum

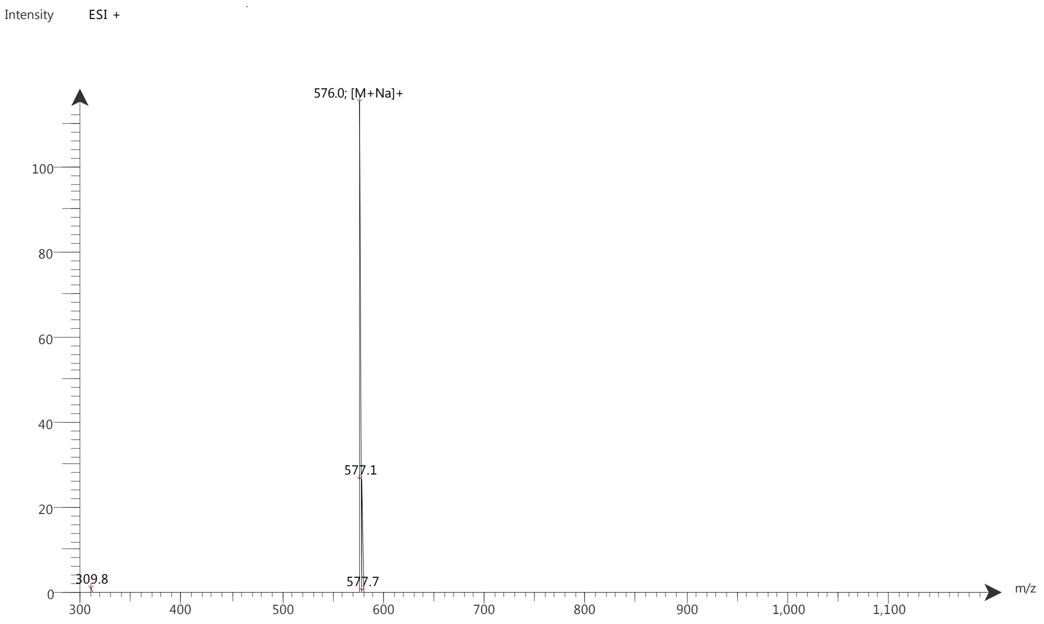

16, [D-Phe(p-NO_2)[4]]CJ-15,208

UHPLC, System 1

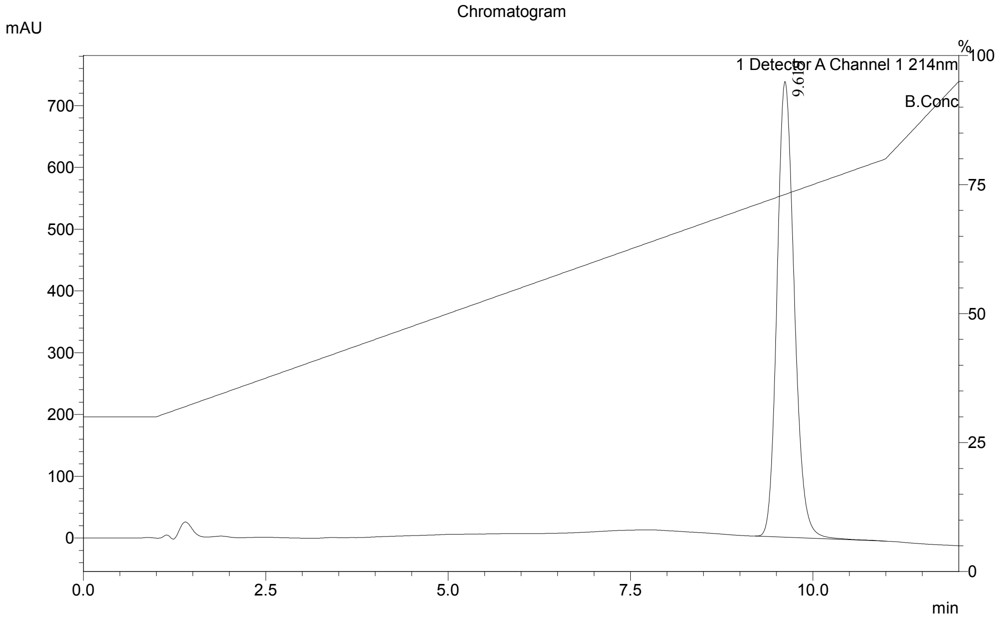

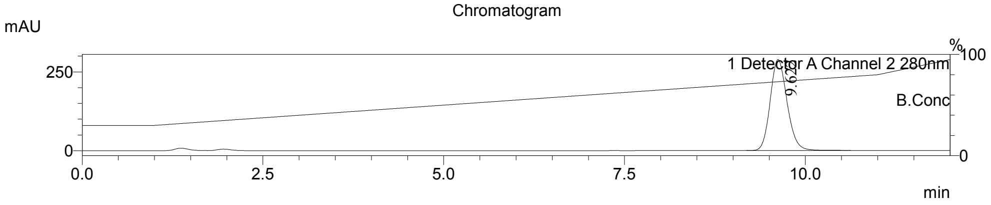

Peak Table

Detector A Channel 1 214nm

Peak#	Ret. Time	Area	Area%
1	9.619	12162935	100.000
Total		12162935	100.000

Peak Table

Detector A Channel 2 280nm

Peak#	Ret. Time	Area	Area%
1	9.622	4687946	100.000
Total		4687946	100.000

16, [D-Phe(p-NO_2)[4]]CJ-15,208

UHPLC, System 2

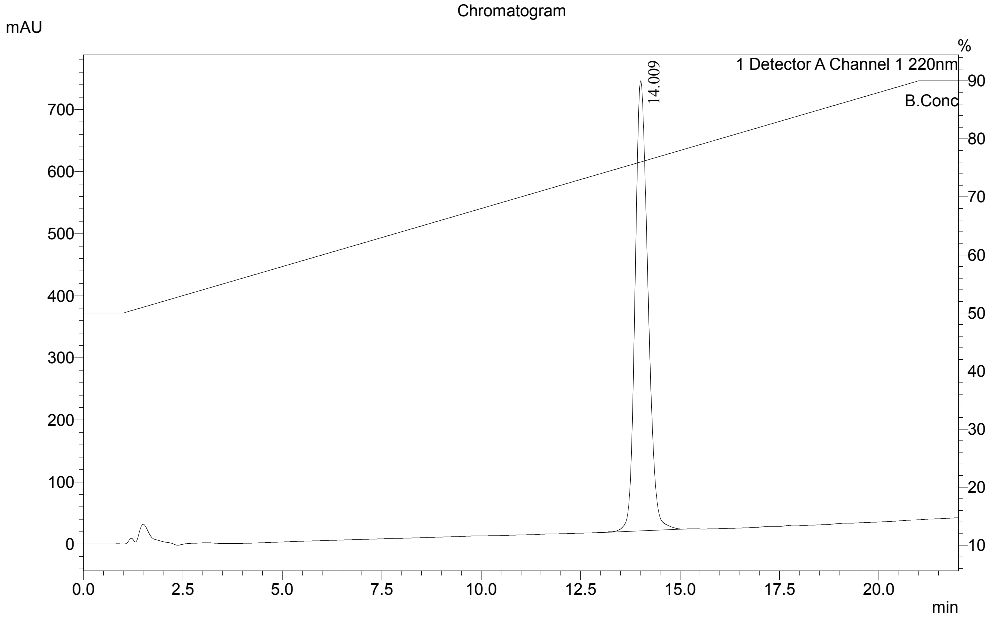

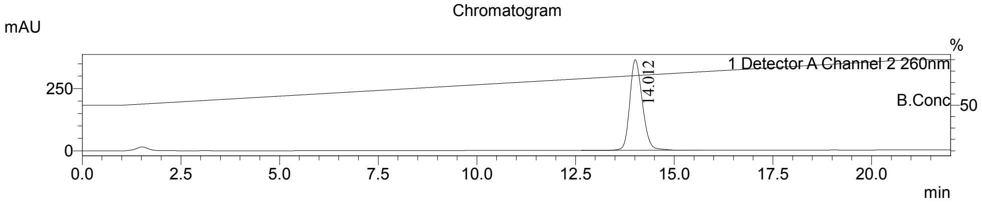

Peak Table

Detector A Channel 1 220nm

Peak#	Ret. Time	Area	Area%
1	14.009	16347681	100.000
Total		16347681	100.000

Peak Table

Detector A Channel 2 260nm

Peak#	Ret. Time	Area	Area%
1	14.012	8077799	100.000
Total		8077799	100.000

16, [D-Phe(p-NO_2)[4]]CJ-15,208

Mass spectrum

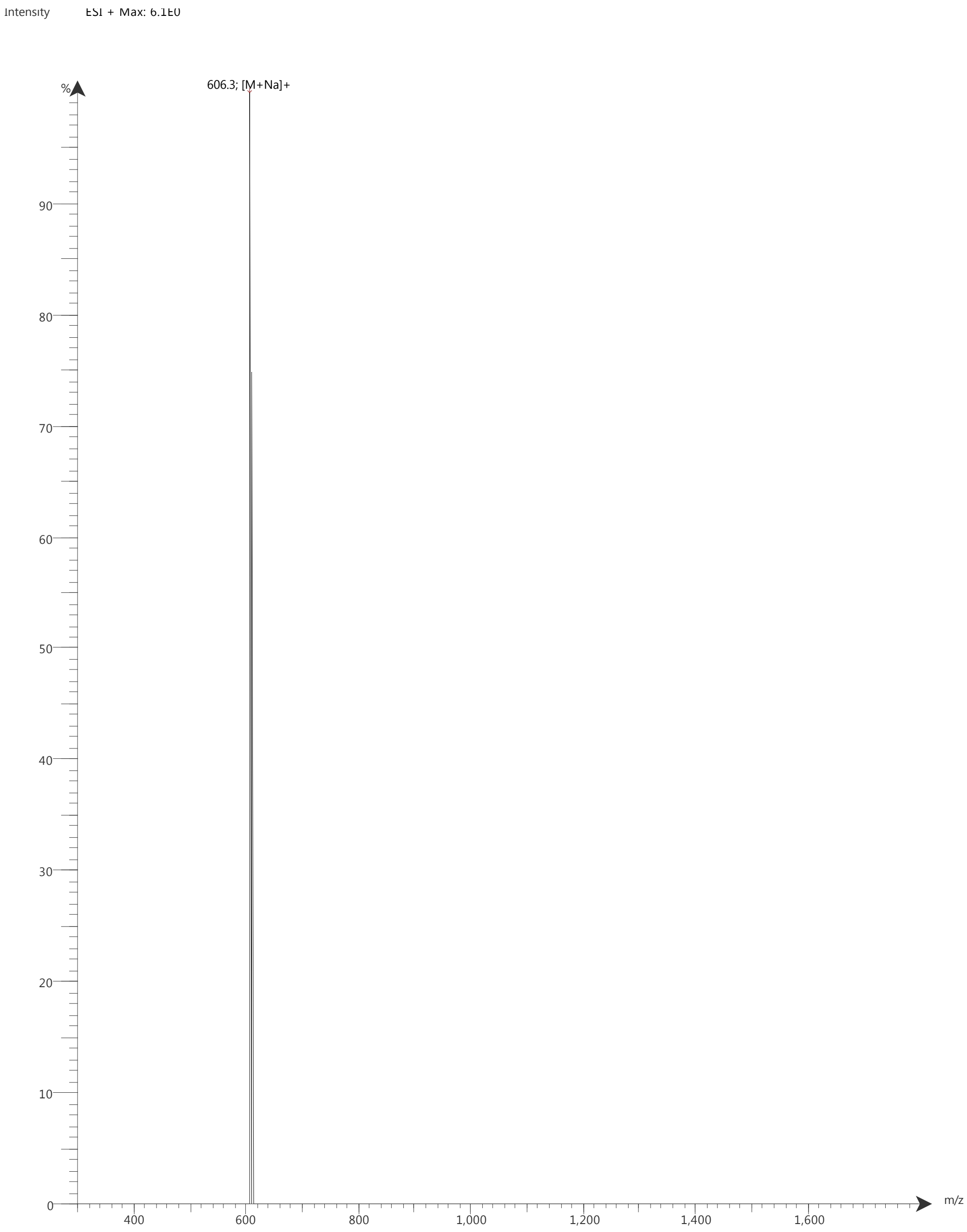

17, [D-Phe(p-NH$_2$)^4]CJ-15,208
UHPLC, System 1

.

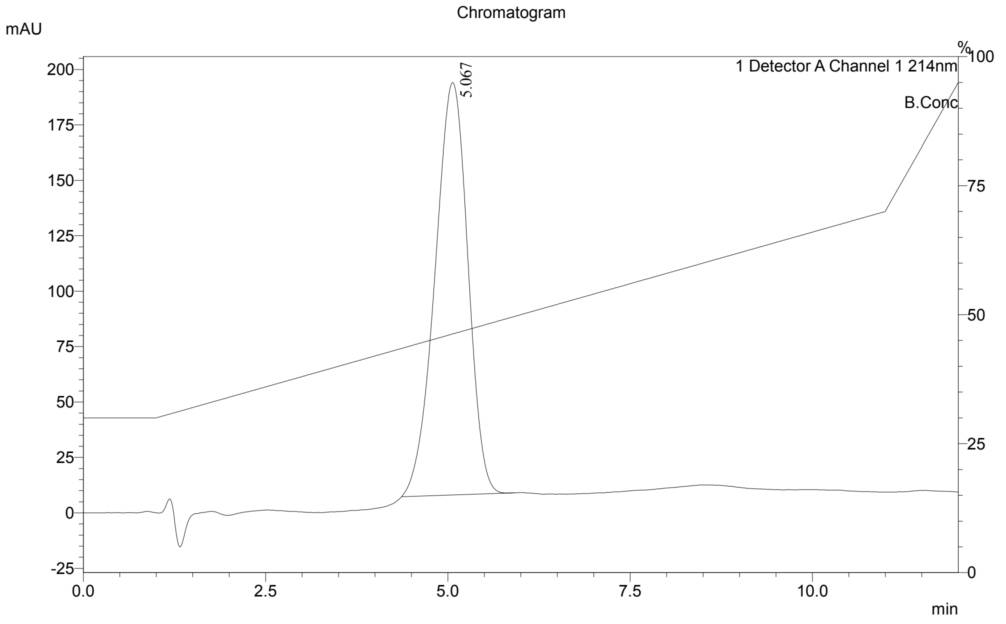

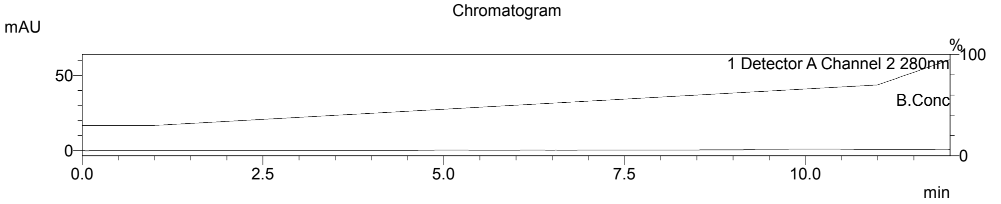

Peak Table
Detector A Channel 1 214nm

Peak#	Ret. Time	Area	Area%
1	5.067	5836560	100.000
Total		5836560	100.000

Peak Table
Detector A Channel 2 280nm

Peak#	Ret. Time	Area	Area%
Total			

17, [D-Phe(p-NH2)4]CJ-15,208

UHPLC, System 2

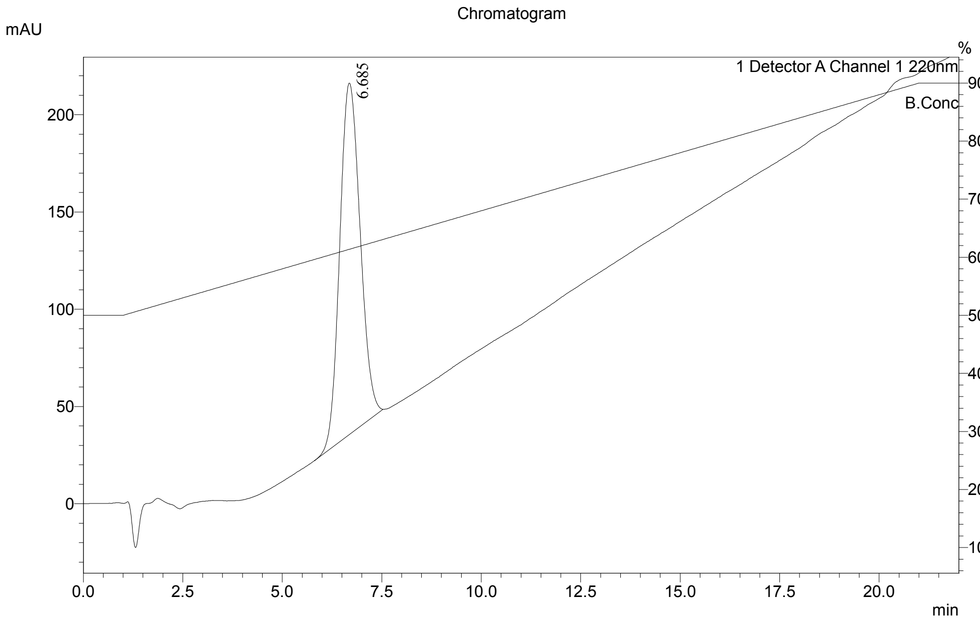

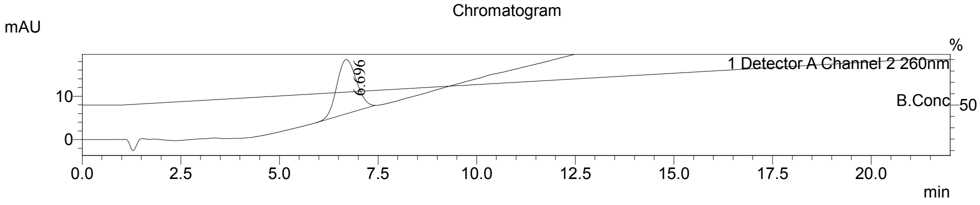

Peak Table

Detector A Channel 1 220nm

Peak#	Ret. Time	Area	Area%
1	6.685	6461427	100.000
Total		6461427	100.000

Peak Table

Detector A Channel 2 260nm

Peak#	Ret. Time	Area	Area%
1	6.696	435653	100.000
Total		435653	100.000

17, [D-Phe(p-NH$_2$)^4]CJ-15,208

Mass spectrum

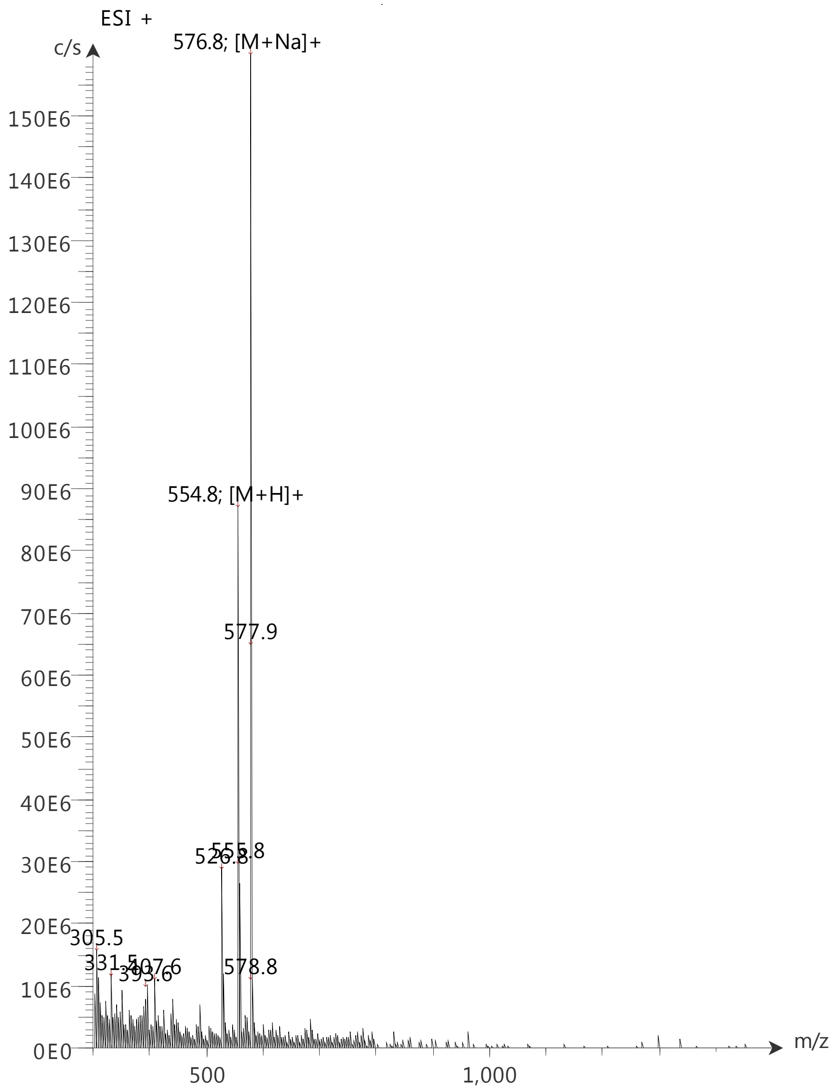

18, [D-Phe(p-OMe)[4]]CJ-15,208

UHPLC, System 1

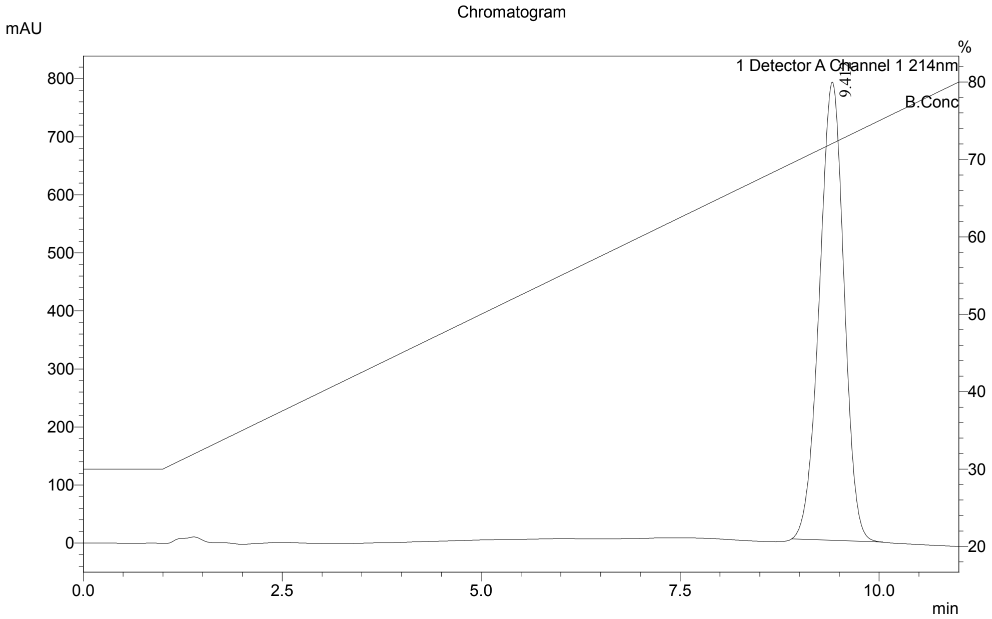

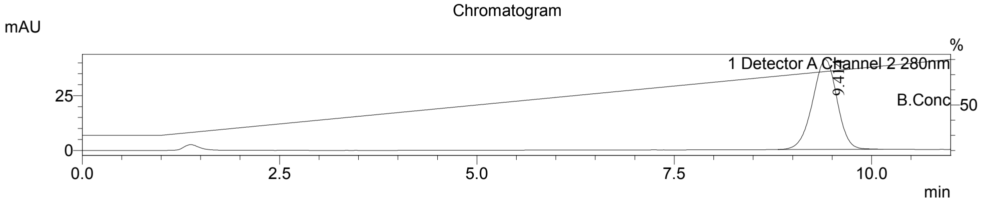

Peak Table

Detector A Channel 1 214nm

Peak#	Ret. Time	Area	Area%
1	9.412	17029802	100.000
Total		17029802	100.000

Peak Table

Detector A Channel 2 280nm

Peak#	Ret. Time	Area	Area%
1	9.414	884391	100.000
Total		884391	100.000

18, [D-Phe(p-OMe)[4]]CJ-15,208
UHPLC, System 2

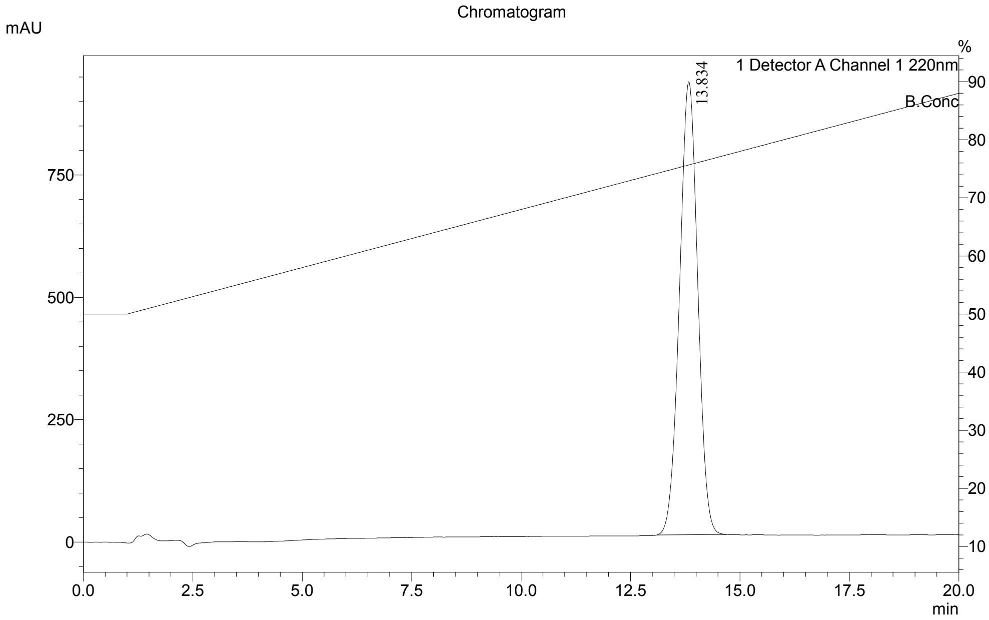

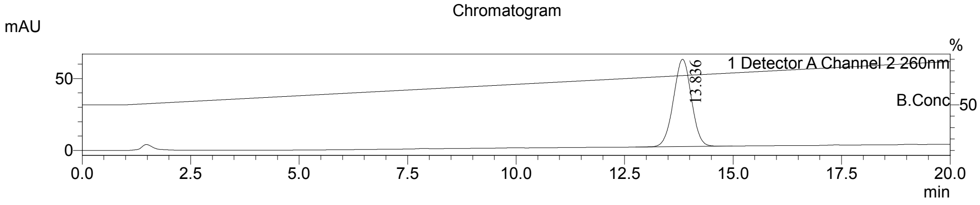

Peak Table
Detector A Channel 1 220nm

Peak#	Ret. Time	Area	Area%
1	13.834	26755964	100.000
Total		26755964	100.000

Peak Table
Detector A Channel 2 260nm

Peak#	Ret. Time	Area	Area%
1	13.836	1750678	100.000
Total		1750678	100.000

18, [D-Phe(p-OMe)[4]]CJ-15,208

Mass spectrum

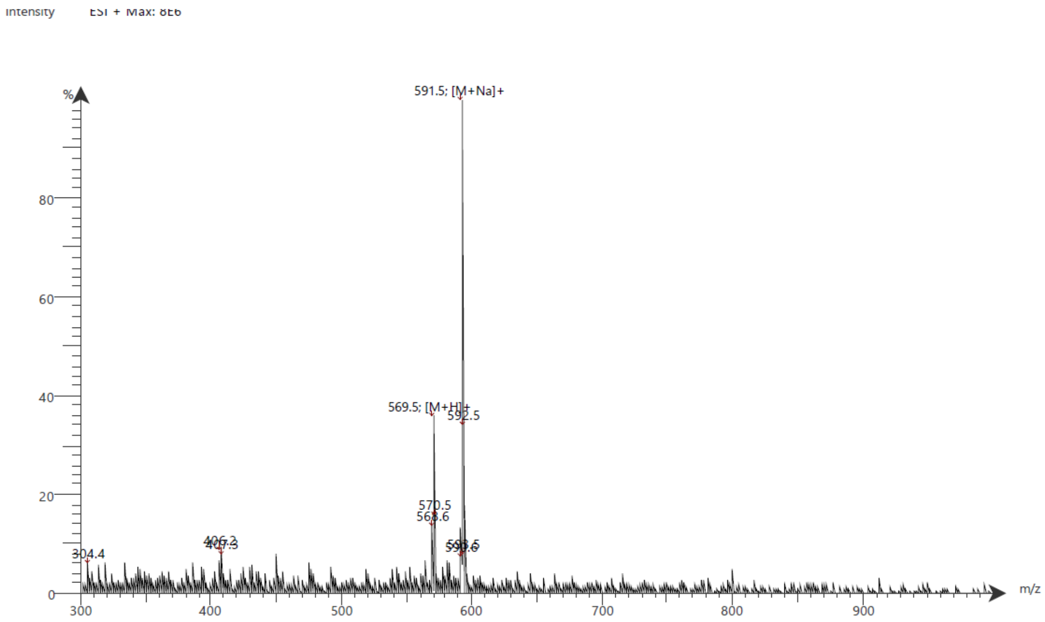

Supplement: Supplementary file 1 [file molecules-30-03993-s001.zip › molecules-3815736-supplementary.pdf]
